# Autophagy is inhibited by ubiquitin ligase activity in the nervous system

**DOI:** 10.1038/s41467-019-12804-3

**Published:** 2019-11-01

**Authors:** Oliver Crawley, Karla J. Opperman, Muriel Desbois, Isabel Adrados, Melissa A. Borgen, Andrew C. Giles, Derek R. Duckett, Brock Grill

**Affiliations:** 1 Department of Neuroscience, The Scripps Research Institute, Jupiter, FL 33458 USA; 2 Department of Molecular Medicine, The Scripps Research Institute, Jupiter, FL 33458 USA; 3 Department of Drug Discovery, Moffitt Cancer Center and Research Institute, Tampa, FL 33612 USA

**Keywords:** Autophagy, Development of the nervous system

## Abstract

Autophagy is an intracellular catabolic process prominent in starvation, aging and disease. Neuronal autophagy is particularly important, as it affects the development and function of the nervous system, and is heavily implicated in neurodegenerative disease. Nonetheless, how autophagy is regulated in neurons remains poorly understood. Using an unbiased proteomics approach, we demonstrate that the primary initiator of autophagy, the UNC-51/ULK kinase, is negatively regulated by the ubiquitin ligase RPM-1. RPM-1 ubiquitin ligase activity restricts UNC-51 and autophagosome formation within specific axonal compartments, and exerts effects broadly across the nervous system. By restraining UNC-51 activity, RPM-1 inhibits autophagosome formation to affect axon termination, synapse maintenance and behavioral habituation. These results demonstrate how UNC-51 and autophagy are regulated subcellularly in axons, and unveils a mechanism for restricting initiation of autophagy across the nervous system. Our findings have important implications beyond nervous system development, given growing links between altered autophagy regulation and neurodegenerative diseases.

## Abstract (web summary)

Despite growing interest in the role of autophagy in neurons, it remains unclear how this process is regulated, in particular, how autophagy is spatially restricted in subcellular compartments in neurons. In this study, the authors use an unbiased proteomic approach to show that the autophagy initiating kinase UNC-51/ULK and autophagosome formation are inhibited by the ubiquitin ligase RPM-1, and demonstrate that this interaction is within specific axonal compartments.

## Introduction

Macroautophagy, here after referred to as autophagy, is a cellular mechanism for degrading long-lived proteins, RNA, and organelles^1,2^. Materials are repurposed via autophagy to maintain homeostasis during nutrient limitation^3^, and declines in autophagy are associated with the key hallmarks of aging^4^. Prominent links between autophagy and human disease are being increasingly documented. For example, abnormal autophagy is implicated in many neurodegenerative diseases, including Alzheimer’s and Parkinson’s disease^5–12^. Genetic studies in rodents showed that impaired autophagy results in increased neuron death and axon degeneration^13–15^. Autophagy is also required in the developing nervous system^6^. Autophagosomes are present in developing axons^16,17^, and autophagy can affect synapse formation and axon growth^18–22^. These broad roles for autophagy in health and disease, within and outside the nervous system, highlight the importance of understanding how autophagy is regulated.

While much progress has been made in deciphering the molecular machinery that executes autophagy, we still know relatively little about how autophagy is regulated. Of particular note is the nervous system, where neurons must manage autophagy differently than other cells for several reasons^6,23^. The nervous system must maintain neuron function during starvation, and as postmitotic cells neurons must manage damaged proteins and organelles over a lifetime. Because of their unique anatomy with extreme distances between axonal compartments and cell bodies, autophagy must be spatially regulated in neurons. Because of these challenges, neurons are likely to deploy additional mechanisms to regulate autophagy. Recent studies identified a small number of players, including Endophilin, Synaptojanin, and Bassoon, that regulate autophagy at presynaptic terminals by acting at downstream steps in the autophagic process^11,12,20^. How initiation of autophagy is regulated in neurons, and if this occurs in specific subcellular compartments, remains unknown.

In nonneuronal cells, a principal mechanism that restrains initiation of autophagy is mTOR inhibition of the UNC-51-like kinase (ULK)^24,25^. However, there is controversy about whether mTOR inhibits autophagy in neurons under normal or neurodegenerative conditions^6,23^. One explanation for this could be that molecular mechanisms other than mTOR restrict initiation of neuronal autophagy. Whether such mechanisms exist has proven challenging to address because ULK has autophagy-independent functions in neurons^24,26,27^. In HeLa cells and muscle, ULK is inhibited by KLHL20, a component of the Cul3 ubiquitin ligase complex^28^. Despite this intriguing precedent, it remains unknown if ubiquitin ligase activity inhibits ULK or autophagy in the nervous system.

Here, we show that RPM-1, an atypical RING E3 ubiquitin ligase in the PAM/Highwire/RPM-1 (PHR) protein family^29–31^, functions via ubiquitin ligase activity to degrade ULK and restrict autophagy broadly in the nervous system of *C. elegans*. Interestingly, RPM-1 inhibits ULK and autophagosome formation in specific axonal compartments. Results from cell-based experiments indicate that the human RPM-1 ortholog, PAM/MYCBP2, also ubiquitinates ULK and affects ULK levels. Thus, PHR proteins could be conserved inhibitors of ULK. To provide a functional context for these observations, we show that inhibition of ULK and autophagy by RPM-1 is required for axon termination, synapse maintenance, and behavioral habituation. These findings reveal how initiation of autophagy is spatially restricted in subcellular compartments during neuron development, and indicate that ubiquitin ligase activity inhibits ULK and autophagy in the nervous system. These findings could have important implications beyond neuronal development as RPM-1, and other PHR proteins, might restrict autophagy in the nervous system during starvation, aging, or neurodegenerative disease. Moreover, inhibitors of autophagy initiation like RPM-1 could be valuable targets for increasing autophagy to treat disease.

## Results

### Proteomics identifies UNC-51 as a putative RPM-1 substrate

**Fig. 1:**
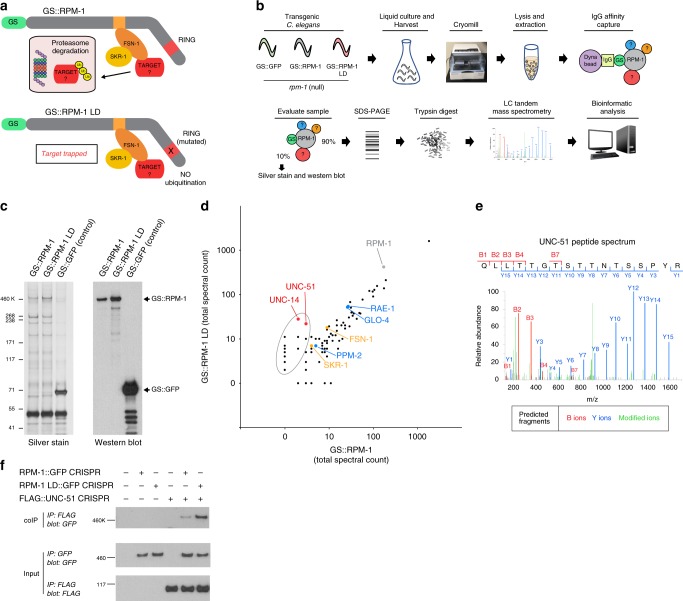
Affinity-purification proteomics from *C. elegans* identifies UNC-51 as a putative RPM-1 ubiquitination substrate. **a** Schematic showing RPM-1 ubiquitin ligase complex, RPM-1 constructs used for proteomics, and RPM-1 LD point mutant that enriches ubiquitination substrates. **b** Experimental workflow for affinity-purification proteomics from *C. elegans*. **c** Example of silver stain (left) and anti-SBP western blot (right) used to evaluate GS::RPM-1, GS::RPM-1 LD, and GS::GFP samples purified from *C. elegans* extracts. **d** Representative example of a single proteomics experiment showing the total spectral counts for individual proteins identified by LC-MS/MS. Shown are the results for GS::RPM-1 LD sample compared with GS::RPM-1 sample with nonspecific hits found in GS::GFP negative control samples above threshold level removed. Highlighted are proteins enriched in RPM-1 LD (gray oval) including UNC-51 and UNC-14 (red), known RPM-1-binding proteins (blue), and RPM-1 ubiquitin ligase complex components (orange). **e** Example of LC-MS/MS peptide spectrum for UNC-51 identified in GS::RPM-1 LD sample. **f** CoIP from *C. elegans* CRISPR strains shows more binding of RPM-1 LD::GFP than RPM-1::GFP to FLAG::UNC-51. Shown is representative from four independent experiments

To uncover putative RPM-1 ubiquitination substrates, we performed a comprehensive series of in vivo affinity-purification proteomics experiments using *C. elegans*. Notably, proteomics remains a highly underutilized approach in *C. elegans*, particularly when studying the nervous system. To identify ubiquitination substrates, we designed a biochemical “trap” using an RPM-1 ligase-dead (RPM-1 LD) site-directed mutant (Fig. 1a). This construct contains mutations within the RING domain (C3535A, H3537A, and H3540A) that impair catalytic activity^32^. Accordingly, RPM-1 LD will bind substrates, but ubiquitination and subsequent downstream proteasomal degradation do not occur^31,33,34^. Thus, we hypothesized that RPM-1 LD would enrich substrates by trapping them in stalled ubiquitination complexes, as well as preventing proteasome-mediated degradation and elevating substrate levels. RPM-1-binding proteins that are not ubiquitination substrates would bind equally to both RPM-1 and RPM-1 LD.

Experiments were designed using animals carrying integrated transgenes expressing either RPM-1, RPM-1 LD, or GFP as a negative control (Fig. 1b). These constructs had a protein-G::streptavidin-binding peptide (GS) tag fused to their N-termini, and were expressed with the native *rpm-1* promoter on an *rpm-1* null background. Transgenic rescue experiments confirmed that GS::RPM-1 is functional, while GS::RPM-1 LD function is impaired (Supplementary Fig. 1). Figure 1b shows a summary of our proteomics workflow in *C. elegans*. Prior to proceeding with in-gel trypsin digestion and mass spectrometry, sample quality was evaluated by silver stain and western blot (Fig. 1c). In total, seven independent affinity-purification proteomics experiments were performed, and positive hits were defined as proteins identified exclusively in RPM-1 or RPM-1 LD samples, or enriched 2x or greater over GFP control samples.

Confirming previous studies^33,35–38^, we identified several known RPM-1-binding proteins, including GLO-4, RAE-1, PPM-2, FSN-1, and SKR-1/SKP1 (Fig. 1d, Table 1; Supplementary Table 1). These positive controls were not enriched in RPM-1 LD samples compared with RPM-1 (Fig. 1d, Table 1; Supplementary Table 1). Our results are consistent with previous work showing these molecules are not RPM-1 ubiquitination substrates. We detected many other putative RPM-1-binding proteins that were present in both RPM-1 and RPM-1 LD samples (Fig. 1d). In contrast, putative ubiquitination substrates were enriched in RPM-1 LD samples compared with RPM-1 (Fig. 1d, gray oval). Detection of much larger numbers of putative RPM-1-binding proteins and putative ubiquitination substrates underscores the heightened scale and sensitivity of proteomics we performed here (see Supplementary Methods), compared with prior proteomics with RPM-1^36–39^.

**Table 1:** Summary of affinity-purification proteomics data from *C. elegans* showing UNC-51 is a putative RPM-1 ubiquitination substrate

					Total peptide spectra			% Sequence coverage		Significance vs GS::GFP (*p*-value)		RPM-1 LD vs RPM-1	
	Protein identified LC-MS/MS	Vertebrate Homolog	MW (kDa)	Total Exp. #	GS:GFP (Control)	GS::RPM-1	GS::RPM-1 LD	GS::RPM-1	GS::RPM-1 LD	GS::RPM-1	GS::RPM-1 LD	Fold increase	Significance (*p*-value)
Purification target	RPM-1	PAM/MYCBP2	418	7	0	1265	2874	51%	62%	—	—	—	—
Known RPM-1-binding protein	RAE-1	RAE1	41	7	0	130	307	61%	74%	3.0 × 10^−4^	1.0 × 10^−6^	1.0×	ns
	GLO-4	RCBTB1/2	153	7	0	116	278	23%	35%	0.016	1.3 × 10^−5^	1.3×	ns
	PPM-2	PP2Cγ	39	5	0	15	23	15%	15%	0.023	2.5 × 10^−3^	0.8×	ns
RPM-1 SCF components	FSN-1	FBXO45	37	7	0	46	147	31%	30%	0.01	4.6 × 10^−4^	1.7×	ns
	SKR-1	SKP1	20	6	0	6	30	31%	55%	ns	3.6 × 10^−3^	1.7×	ns
Putative RPM-1 ubiquitination substrates	**UNC-51**	ULK1	95	7	0	11	123	7%	28%	0.041	**7.0** × **10**^**−6**^	**5.8**×	**4.8** × **10**^**−6**^
	**UNC-14**	RUSC2	74	7	0	8	179	8%	36%	0.041	**8.1** × **10**^**−6**^	**12.3**×	**1.8** × **10**^**−6**^

Results and analysis from seven independent proteomics experiments using *C. elegans* expressing GS::RPM-1, GS::RPM-1 LD, or GS::GFP. UNC-51 and UNC-14 are significantly enriched in GS::RPM-1 LD samples, detected at very low levels in GS::RPM-1 samples, and not present in GS::GFP-negative control samples. Note 5.8× more UNC-51 peptides were detected in GS::RPM-1 LD samples compared with GS::RPM-1 samples. Significance determined using Student’s *t* test

Interestingly, several proteins were consistently enriched in RPM-1 LD compared with RPM-1 samples suggesting our “trap” for RPM-1 ubiquitination substrates was successful (Fig. 1d). Two prominently enriched proteins in RPM-1 LD samples were the autophagy initiating kinase UNC-51 and the RUN domain protein UNC-14 (Fig. 1d, e; Table 1; Supplementary Table 1; Supplementary Fig. 2). UNC-14 is a known UNC-51-binding protein^26^, which indicates we may have identified UNC-51/UNC-14 complexes.

To validate our proteomic results, we performed coimmunoprecipitation (coIP) experiments using CRISPR/Cas9-engineered worms. CRISPR was used to fuse GFP with RPM-1, generate an RPM-1 LD::GFP mutation, and engineer a FLAG tag on UNC-51. CoIP from whole-worm lysates showed RPM-1 LD::GFP displays more binding to FLAG::UNC-51 than RPM-1::GFP (Fig. 1f). Thus, the results from affinity-purification proteomics and coIP biochemistry indicate that UNC-51 is a putative RPM-1 ubiquitination substrate.

### Axon termination requires UNC-51 inhibition by RPM-1

Prior work established that RPM-1 regulates axon termination in *C. elegans* mechanosensory neurons^36,40^. Developmental time course and in vivo live imaging studies showed that RPM-1 is initially localized to the growth cone where it regulates a protracted growth cone collapse process that results in axon termination^41^. Following termination of axon outgrowth, RPM-1 remains localized at the terminated axon tip. RPM-1 function is mediated, in part, by ubiquitination and proteasomal degradation of substrates^29,31,34^. With proteomic and biochemical results suggesting that UNC-51 is a putative RPM-1 ubiquitination substrate (Fig. 1, Table 1), we turned to genetic approaches to test the hypothesis that RPM-1 regulates axon termination by inhibiting UNC-51.

**Fig. 2:**
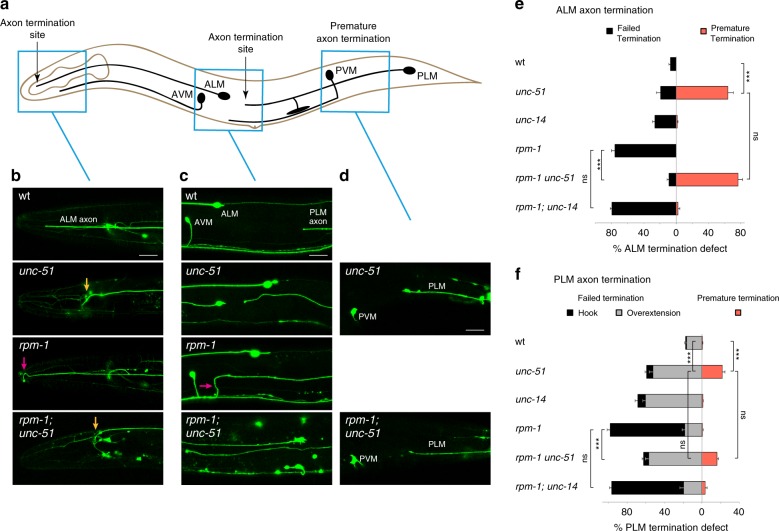
RPM-1 inhibits UNC-51 to regulate axon termination. **a** Schematic of *C. elegans* mechanosensory neurons. Blue boxes highlight locations of ALM and PLM axons imaged in panels below by confocal microscopy. **b** Representative images of ALM axons for indicated genotypes visualized using transgenic GFP. Note failed termination (magenta arrow) in *rpm-1* mutants compared with premature termination (orange arrow) in both *unc-51* single mutants and *rpm-1; unc-51* double mutants. **c** Representative images of PLM axons for indicated genotypes. *rpm-1* mutants display severe failed termination defects (hook, magenta arrow), while *rpm-1; unc-51* double mutants display less severe failed termination defects (overextension). **d** Representative images of premature termination defects observed in PLM neurons of *unc-51* single mutants and *rpm-1; unc-51* double mutants. **e** Quantitation of failed termination and premature termination defects in ALM neurons for indicated genotypes. Failed termination defects are suppressed in *rpm-1; unc-51* double mutants, but not *rpm-1; unc-14* double mutants. **f** Quantitation of failed termination and premature termination defects in PLM neurons for indicated genotypes. Severe hook defects are suppressed in *rpm-1; unc-51* double mutants, but not *rpm-1; unc-14* double mutants. Means are shown from 5 to 8 counts (25–35 animals/count) for each genotype, and error bars represent SEM. Significance determined using Student’s *t* test with Bonferroni correction. ***p < 0.001; ns, not significant. Scale bars 20 μm. Source data are provided as a Source Data file

In *C. elegans*, there are two anterior ALM mechanosensory neurons. Each ALM neuron projects a single axon that terminates its primary process before the nose in wt animals (Fig. 2a, b). Axon termination fails in *rpm-1* mutants resulting in overgrown axons (Fig. 2b, e)^40^. Consistent with prior studies^42^, we observed defective axon guidance in strong loss-of-function (lf) mutants for *unc-51*, but this did not prevent us from evaluating axon termination. ALM axons in *unc-51* mutants frequently displayed premature termination, the opposing phenotype to *rpm-1* mutants (Fig. 2b, e). This result is consistent with prior studies in mice, which showed that impairing autophagy reduces axon outgrowth^21,22^. *rpm-1; unc-51* double mutants showed complete suppression of the failed termination phenotype in *rpm-1* single mutants, and presented similar frequency of premature termination defects as *unc-51* single mutants (Fig. 2b, e). Thus, in *rpm-1; unc-51* double mutants the *unc-51* (lf) phenotype dominates, which suggests RPM-1 functions as an upstream inhibitor of UNC-51.

To further explore this genetic relationship, we evaluated the PLM mechanosensory neurons. Each PLM neuron has a primary axon that terminates in the midbody of wt animals (Fig. 2a, c). *rpm-1* mutants displayed severe, frequent failed termination defects in which PLM axons extend past ALM cell bodies and hook ventrally (Fig. 2c, f)^40,43^. Interestingly, *unc-51* mutants showed two phenotypes: premature termination (Fig. 2d, f), and failed termination in which PLM axons overextend beyond ALM cell bodies but do not hook ventrally (Fig. 2c, f). The distinction between failed termination defects that are weak (overextension) and severe (hook) is well documented^36–38,43^. Similar to the results with ALM neurons, *rpm-1; unc-51* double mutants showed only the *unc-51* single mutant phenotype in PLM neurons, indicating that *rpm-1* (lf) is suppressed by *unc-51* (lf) (Fig. 2c, d, f).

As an independent approach for evaluating the functional relationship between *unc-51* and *rpm-1*, we expressed an UNC-51 dominant negative (UNC-51 DN) construct that is catalytically inactive and inhibits UNC-51 function^44,45^. Mos single copy insertion (MosSCI) was used to express UNC-51 DN in the mechanosensory neurons of *rpm-1* mutants. We observed robust suppression of failed termination defects in *rpm-1* mutants expressing UNC-51 DN (Supplementary Fig. 3). This provided further evidence that RPM-1 inhibits UNC-51. Importantly, expression of UNC-51 DN alone or in *rpm-1* mutants did not affect PLM axon guidance (Supplementary Fig. 4). As a result, the UNC-51 DN was particularly valuable for testing the genetic relationship between *unc-51* and *rpm-1* in axon termination without complications from effects on axon guidance.

UNC-14, a known UNC-51-binding protein that functions with UNC-51 to affect axon guidance^26,27^, was also enriched in RPM-1 LD proteomic samples (Fig. 1d, Table 1; Supplementary Table 1). Therefore, we evaluated how *unc-14* (lf) interacts with *rpm-1* (lf). In contrast to *unc-51*, we observed no premature termination defects in ALM (Fig. 2e) or PLM neurons (Fig. 2f) for *unc-14* (lf) mutants. Quantitation of ALM and PLM phenotypes showed that failed termination defects caused by *rpm-1* (lf) were not suppressed in *rpm-1; unc-14* double mutants (Fig. 2e, f). These genetic results suggest that UNC-14 was enriched in RPM-1 LD proteomic samples because UNC-51 binds UNC-14, but that these interactions do not pertain to RPM-1 signaling in axon termination. Furthermore, this was a second independent result that suggested RPM-1 effects on UNC-51 in axon termination are molecularly distinct from the role UNC-51 plays in axon guidance.

Taken as a whole, our genetic results support several conclusions. (1) UNC-51 is required to prevent premature termination. (2) UNC-51 is negatively regulated by RPM-1. (3) RPM-1 inhibition of UNC-51 is required for proper axon termination, but does not pertain to UNC-51/UNC-14 effects on axon guidance. These genetic findings are consistent with our proteomic and biochemical results that suggest UNC-51 is a putative RPM-1 ubiquitination substrate.

### RPM-1 inhibits UNC-51 to promote synapse maintenance and habituation

**Fig. 3:**
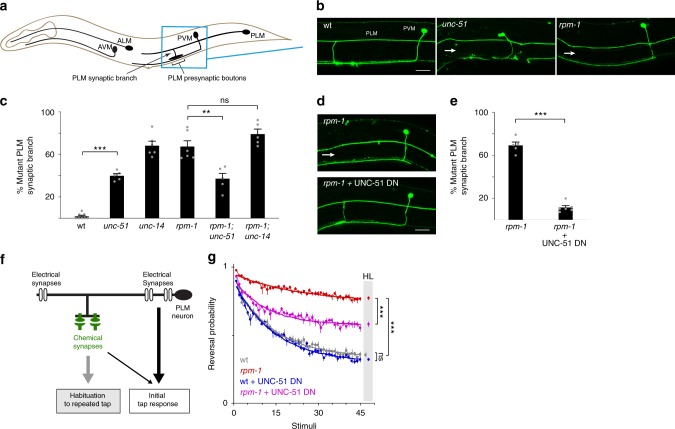
*unc-51* (lf) and UNC-51 DN expression suppress synapse maintenance defects in *rpm-1* mutants. **a** Schematic of *C. elegans* mechanosensory neurons. Blue box highlights collateral synaptic branch and presynaptic boutons of PLM neurons imaged using confocal microscopy. PVM neuron is not PLM synaptic target, but is an anatomical marker for branch and bouton position. **b** Representative images of synaptic branch and presynaptic boutons for PLM neurons of indicated genotypes. Arrow indicates synapse maintenance defects, in which synaptic branch is retracted in *rpm-1* mutants^46^. Absence of synaptic branch also occurs in *unc-51* mutants. **c** Quantitation of synapse maintenance defects for indicated genotypes. Frequency of defects in *rpm-1; unc-51* double mutants is suppressed to levels in *unc-51* single mutants. Suppression does not occur in *rpm-1; unc-14* double mutants. **d** Representative images showing synapse maintenance defects (arrow) in *rpm-1* mutants are suppressed when UNC-51 DN is expressed using MosSCI. **e** Quantitation shows synapse maintenance defects in *rpm-1* mutants are suppressed by UNC-51 DN. **f** Summary of chemical and electrical synapses in PLM neurons that contribute to tap response and behavioral habituation. Loss of chemical synapses primarily affects habituation to repeated tap. **g** Multi-worm tracker quantitation of responses to repeated tap stimuli for indicated genotypes. Habituation defects in *rpm-1* mutants are suppressed by MosSCI expression of UNC-51 DN in mechanosensory neurons. For synapse maintenance, means are shown from 5 to 8 counts (25–35 animals/count) for each genotype, and error bars represent SEM. For habituation, means are from 16 plates (~50 animals per plate) for each genotype, error bars represent SEM, curves are best fit exponentials, and habituation level (HL) is fit at final stimulus. Significance determined using Student’s *t* test with Bonferroni correction. **p < 0.01; ***p < 0.001; ns, not significant. Scale bars 20 μm. Source data are provided as a Source Data file

Another established function of RPM-1 in mechanosensory neurons is promoting synapse formation^36,40^. Developmental time-course studies demonstrated this is due to RPM-1 effects on synapse maintenance^46^. PLM mechanosensory neurons form chemical synapses with interneurons via a collateral branch that extends ventrally from the primary axon (Fig. 3a, b). In *rpm-1* mutants, failed synapse maintenance results in loss of presynaptic boutons and synaptic branch retraction (Fig. 3b, c)^46^. We also observed synaptic branch loss in *unc-51* mutants (Fig. 3b). However, quantitation showed that the frequency of synaptic branch defects was higher in *rpm-1* mutants, which allowed room to evaluate suppression (Fig. 3c). We observed significant suppression of synaptic branch defects in *rpm-1; unc-51* double mutants down to frequencies observed in *unc-51* single mutants (Fig. 3c). *unc-14* mutants showed higher frequency defects than *unc-51* single mutants, and suppression was not observed in *rpm-1; unc-14* double mutants (Fig. 3c).

Because synaptic branch defects were observed in *unc-51* (lf) mutants, we turned to a second, independent loss-of-function approach, in which UNC-51 DN was expressed in mechanosensory neurons using MosSCI. Expression of UNC-51 DN in *rpm-1* mutants resulted in much stronger suppression of synaptic branch defects than what was observed in *rpm-1; unc-51* double mutants (Fig. 3d, e). This provided more evidence that impairing *unc-51* function suppresses synapse maintenance defects caused by *rpm-1* (lf).

To further examine how expressing UNC-51 DN affects *rpm-1* (lf), we evaluated habituation to repeated tap stimulus. Glutamatergic chemical synapses formed by PLM neurons are an important connection within the neural circuit that mediates habituation to repeated tap (Fig. 3f)^47,48^. As a result, synapse maintenance defects in the mechanosensory neurons of *rpm-1* mutants result in pronounced habituation defects (Fig. 3g)^46,49,50^. Consistent with this, mutations that suppress defective synapse maintenance in *rpm-1* mutants also suppress habituation defects^49,50^. Because electrical synapses (which primarily mediate the initial response to tap, Fig. 3f) are not impaired in *rpm-1* mutants, these animals sense initial tap normally^41^. We found that expression of UNC-51 DN in the mechanosensory neurons of wild-type animals did not affect habituation to repeated tap (Fig. 3g). However, UNC-51 DN strongly suppressed habituation defects in *rpm-1* mutants (Fig. 3g).

Thus, findings with *unc-51* (lf) mutants and an UNC-51 DN construct indicate that RPM-1 inhibits UNC-51 to affect synapse maintenance and habituation. This is unlikely to be a secondary consequence of UNC-51 effects on axon guidance for two reasons. First, expression of UNC-51 DN (which does not affect axon guidance when expressed at single copy, Supplementary Fig. 4) strongly suppresses synapse maintenance and habituation defects in *rpm-1* mutants. Second, our findings indicate that UNC-51 and UNC-14 have different functional relationships with RPM-1 during synapse maintenance. This separates RPM-1 effects on UNC-51 in synapse maintenance from molecular mechanisms that operate with UNC-51 to regulate axon guidance.

### UNC-51 kinase activity functions in mechanosensory neurons

With genetic outcomes suggesting that RPM-1 inhibits UNC-51, we wanted to test two questions. Does *unc-51* kinase activity function in mechanosensory neurons to regulate axon termination and synapse maintenance? Does UNC-51 overexpression affect neuron development?

**Fig. 4:**
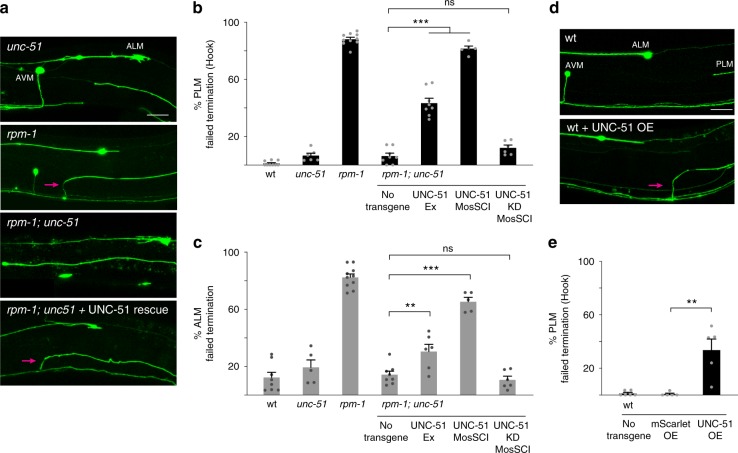
UNC-51 kinase activity functions cell autonomously to negatively regulate axon termination. **a** Representative images of PLM axons for indicated genotypes. Severe failed termination defects (magenta arrow) in *rpm-1* mutants are suppressed in *rpm-1; unc-51* double mutants and rescued by expressing UNC-51 in mechanosensory neurons. **b** Quantitation of failed termination defects in PLM neurons for indicated genotypes (Ex, extrachromosomal array, KD, kinase dead). **c** Quantitation of failed termination defects in ALM neurons for indicated genotypes. **d** Representative images of PLM neurons for indicated genotypes. Transgenic overexpression of UNC-51 in mechanosensory neurons causes severe failed termination defects (magenta arrow, hook). **e** Quantitation of severe failed termination defects (hook) in PLM neurons for indicated genotypes. Means are shown from 5 to 8 counts (20–35 animals/count) for each genotype, and error bars represent SEM. Significance determined using Student’s *t* -test with Bonferroni correction. **p < 0.01; ***p < 0.001; ns, not significant. Scale bars 20 μm. Source data are provided as a Source Data file

To address the first question, we generated animals with transgenic extrachromosomal arrays that used a mechanosensory neuron promoter to express UNC-51. Suppression of failed termination defects in PLM and ALM neurons of *rpm-1; unc-51* double mutants was rescued by expression of UNC-51 (Fig. 4a–c). Likewise, suppression of synapse maintenance defects in *rpm-1; unc-51* mutants was rescued by transgenic UNC-51 (Supplementary Fig. 5a, b). Because rescues were partial with extrachromosomal arrays for axon termination (Fig. 4b, c), we expressed UNC-51 in mechanosensory neurons using MosSCI. This approach also provided an ideal setting to test whether loss of UNC-51 kinase activity suppresses *rpm-1*. MosSCI expression of wt UNC-51 resulted in robust rescue of suppression in *rpm-1; unc-51* double mutants (Fig. 4b, c; Supplementary Fig. 5a, b). Rescue was not observed with an UNC-51 kinase-dead (KD) point mutant (Fig. 4b, c; Supplementary Fig. 5a, b). Thus, UNC-51 kinase activity functions cell autonomously in mechanosensory neurons to regulate axon termination and synapse maintenance. Similar findings on cell autonomy were made previously for RPM-1^40,51^.

Our findings demonstrate that *unc-51* (lf) suppresses *rpm-1* (lf) (Fig, 2, 3), and that UNC-51 kinase activity is required to rescue suppression (Fig. 4b, c; Supplementary Fig. 5b). This suggested that *rpm-1* mutants might have excess UNC-51 kinase activity, which contributes to defects in axon termination and synapse maintenance. To test this, we transgenically overexpressed UNC-51 specifically in the mechanosensory neurons of wt animals. Indeed, PLM neurons overexpressing UNC-51 showed failed termination defects, reminiscent of *rpm-1* (lf) mutants, in which PLM axons hooked ventrally (Fig. 4d, e). Overexpression of UNC-51 in wt animals also resulted in synaptic branch defects similar to *rpm-1* (lf) mutants (Supplementary Fig. 5c, d). Thus, transgenic rescue and overexpression results indicate that UNC-51 kinase activity functions cell autonomously in mechanosensory neurons to inhibit axon termination and synapse maintenance.

### RPM-1 restricts UNC-51 in subcellular compartments and across the nervous system

Proteomic and biochemical results suggested that UNC-51 is an RPM-1 ubiquitination substrate (Fig. 1). Consistent with this, RPM-1 inhibits UNC-51 to regulate axon termination and synapse maintenance in mechanosensory neurons (Figs. 2–4). One model supported by these results is that RPM-1 ubiquitination of UNC-51 leads to degradation of UNC-51 by the proteasome. To further test this, we evaluated how RPM-1 affects UNC-51 protein levels in mechanosensory neurons and across the nervous system of *C. elegans*.

**Fig. 5:**
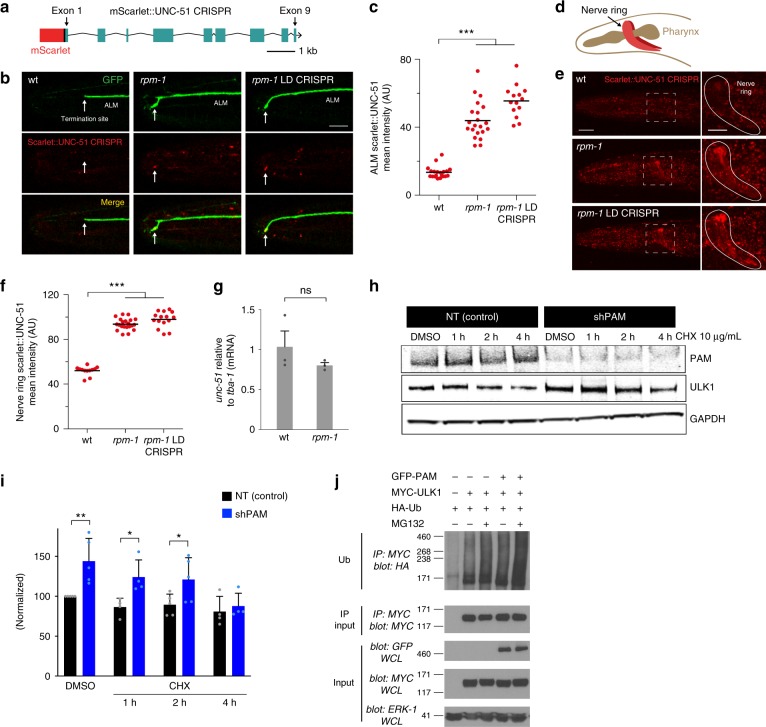
RPM-1 restricts UNC-51 levels in axonal compartments, broadly across the nervous system, and has conserved effects on human ULK1. **a** Diagram showing CRISPR engineering of mScarlet into *C. elegans unc-51* gene. **b** Representative confocal images showing mScarlet::UNC-51 CRISPR in axons of ALM neurons for indicated genotypes. Transgenic GFP is used to visualize axon termination site (white arrow). In *rpm-1* (lf) mutants and *rpm-1* LD CRISPR animals, mScarlet::UNC-51 accumulates in puncta at the tips of axons with failed termination (white arrows). Scale bar 10 μm. **c** Quantitation indicates mScarlet::UNC-51 is significantly increased at tips of axons in *rpm-1* (lf) mutants and *rpm-1* LD CRISPR mutants compared with wt animals. **d** Schematic showing axon bundle in the nerve ring of *C. elegans*. **e** Representative confocal images showing mScarlet::UNC-51 CRISPR in nerve ring for indicated genotypes. mScarlet::UNC-51 is increased in nerve ring of *rpm-1* (lf) mutants and *rpm-1* LD CRISPR animals. Scale bars 20 μm (zoom-out, left), 10 μm (zoom-in, right). **f** Quantitation showing mScarlet::UNC-51 is significantly increased in nerve ring of *rpm-1* (lf) mutants and *rpm-1* LD CRISPR mutants compared to wt animals. **g** Quantitation indicates *unc-51* mRNA levels are unchanged in *rpm-1* mutants compared to wt. **h** Representative western blots from human SH-SY5Y neuroblastoma cells showing endogenous ULK1 is increased when cells are infected with lentivirus expressing shRNA against human *PAM*. ULK1 increases caused by *PAM* knockdown are also observed when translation is inhibited with cycloheximide (CHX). **i** Quantitation shows ULK1 levels are significantly elevated when PAM is knocked down in SH-SY5Y. Shown are means with SEM from four independent experiments. **j** GFP-PAM polyubiquitinates MYC-ULK1 in HEK 293 cells. PAM polyubiquitination of ULK1 is increased when the proteasome is inhibited with MG132. Shown is a representative of three independent experiments. For **c** and **f**, means are shown from 12 or more animals imaged during 3–4 independent experiments for each genotype, and significance was determined using Student’s *t* test with Bonferroni correction. For **i**, significance was tested using two-way ANOVA with post hoc Student’s *t* test. *p < 0.05, **p < 0.01, ***p < 0.001. Source data are provided as a Source Data file

To evaluate endogenous UNC-51, we used CRISPR/Cas9 to engineer a fusion of mScarlet with UNC-51 (Fig. 5a). Two observations suggested the fluorescent tag did not interfere with UNC-51 function. First, transgenic rescue experiments showed UNC-51 is functional when fused with mCherry, another large tag (Supplementary Fig. 6). Second, we observed no premature termination defects or axon guidance defects in the mechanosensory neurons of Scarlet::UNC-51 CRISPR animals (Fig. 5b, not shown). We combined Scarlet::UNC-51 CRISPR with transgenic cell-specific GFP to evaluate levels and localization of UNC-51 in mechanosensory neurons. As shown in Fig. 5b, we observed very little Scarlet::UNC-51 at axon termination sites of ALM neurons in wt animals. Similarly, axon termination sites in PLM neurons had extremely low levels of Scarlet::UNC-51 CRISPR (Supplementary Fig. 7a). In contrast, we observed Scarlet::UNC-51 puncta accumulating strongly at tips of ALM and PLM axons that failed to terminate in *rpm-1* (lf) mutants (Fig. 5b; Supplementary Fig. 7a). Quantitation showed significant increases in UNC-51 levels at axon tips of *rpm-1* mutants compared with wt animals (Fig. 5c; Supplementary Fig. 7b).

To test whether RPM-1 ubiquitin ligase activity restricts UNC-51 accumulation at axon termination sites, we CRISPR edited point mutations into the *rpm-1* locus to generate RPM-1 LD, which lacks ligase activity (Fig. 1a). Compared with wt animals, Scarlet::UNC-51 CRISPR was significantly increased at sites of failed axon termination in ALM neurons of *rpm-1* LD CRISPR animals, similar to what occurred in *rpm-1* (lf) mutants (Fig. 5b, c). Similar outcomes occurred at failed axon termination sites of PLM neurons in *rpm-1* LD CRISPR animals (Supplementary Fig. 7a). These results demonstrate that RPM-1 functions via ubiquitin ligase activity to restrict UNC-51 specifically at axon termination sites.

Next, we set out to test another question: does RPM-1 ubiquitin ligase activity broadly restrict UNC-51 across the nervous system? To address this, we assessed Scarlet::UNC-51 CRISPR in the nerve ring (Fig. 5d). The nerve ring is the largest bundle of axons in *C. elegans* containing processes from over half the animal’s neurons. Therefore, the nerve ring serves as a proxy for evaluating broad effects across the nervous system. Similar to findings in mechanosensory neurons, wt animals showed very little expression of Scarlet::UNC-51 CRISPR in the nerve ring (Fig. 5e). In fact, UNC-51 was more evident in the head of wt animals outside the nerve ring, likely representing nonneuronal UNC-51. UNC-51 levels in the nerve ring were markedly increased in *rpm-1* (lf) mutants and *rpm-1* LD CRISPR animals (Fig. 5e, f). *rpm-1* (lf) mutants and *rpm-1* LD CRISPR animals also had increased UNC-51 puncta at the tip of the nose and around the edge of the head where large numbers of axons project (Fig. 5e).

To determine if RPM-1 effects on UNC-51 protein levels were due to changes in transcription, we performed qPCR. *unc-51* mRNA levels were unchanged relative to several stable mRNA species (e.g. tubulin *tba-1*) in *rpm-1* mutants compared with wt animals (Fig. 5g; Supplementary Fig. 8). Thus, effects of RPM-1 on UNC-51 are not due to changes in *unc-51* transcription.

Overall, these results support several conclusions. (1) RPM-1 functions via ubiquitin ligase activity to regulate endogenous UNC-51 levels in neurons in vivo. (2) RPM-1 spatially restricts UNC-51 at terminated axon tips of mechanosensory neurons. (3) RPM-1 broadly restricts UNC-51 across the nervous system.

### Human PAM ubiquitinates ULK1 and regulates ULK1 levels

Having shown that RPM-1 ubiquitin ligase activity restricts UNC-51 in the *C. elegans* nervous system in vivo, we wanted to explore whether RPM-1 effects on UNC-51 are conserved. In humans, there is a sole RPM-1 ortholog called PAM or MYCBP2^29^. UNC-51 has two human orthologs that initiate autophagy, ULK1 and ULK2^24^. To determine if PAM affects the levels of ULK1, we infected SH-SY5Y human neuroblastoma cells with lentivirus expressing an shRNA against *PAM*. As shown in Fig. 5h, *PAM* shRNA reduced PAM levels compared with expression of a non-targeting shRNA control. Quantitation showed ULK1 levels were significantly increased in *PAM* knockdown cells (Fig. 5i). This effect could also be observed when translation was inhibited with cycloheximide (Fig. 5h, i). Cycloheximide was applied to facilitate turnover of ULK1 and possibly accentuate effects of PAM knockdown on ULK1 levels, but this did not occur. This suggests that ULK1 is a relatively stable protein, and is notably different from the PAM substrate NMNAT2, which turns over rapidly^31^. Nonetheless, these results are consistent with PAM restricting ULK1 levels in human neuroblastoma cells.

To determine why ULK1 levels are elevated in PAM knockdown cells, we tested if ULK1 is ubiquitinated and if PAM is sufficient for ULK1 ubiquitination. For these experiments, we turned to HEK 293T cells because they are easily transfected with multiple constructs at high efficiency, and PAM ubiquitination of NMNAT2 was previously detected in these cells^31^. 293 cells were co-transfected with HA-Ubiquitin (HA-Ub) and MYC-ULK1 with or without GFP-PAM. ULK1-MYC was immunoprecipitated from cell lysates and immunoblotted for HA-Ub. We observed low levels of ULK1 polyubiquitination (size > 171 kDa), which was increased by the proteasome inhibitor MG132 (Fig. 5j). ULK1 ubiquitination was also increased with co-transfection of GFP-PAM (Fig. 5j). The most pronounced increase in ULK1 polyubiquitination occurred when both GFP-PAM and MG132 were present (Fig. 5j, last lane). These results show that PAM is sufficient to trigger polyubiquitination of ULK1, and that PAM ubiquitination of ULK1 is affected by the proteasome. These cell-based results are consistent with our in vivo proteomic, biochemical, and genetic findings from *C. elegans*, and suggest that PHR proteins could be evolutionarily conserved inhibitors of ULK.

### UNC-51 and RPM-1 colocalize at axon termination sites

**Fig. 6:**
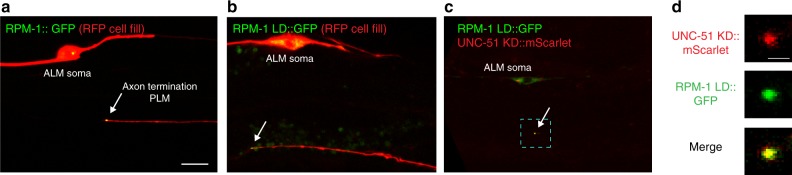
RPM-1 colocalizes with UNC-51 at axon termination sites. Shown are representative confocal images of axons from PLM neurons of adult wt animals. **a** RPM-1::GFP and (**b**) RPM-1 LD::GFP (which lacks ubiquitin ligase activity) accumulate in puncta at axon tips (arrows). Scale bar 10 μm (**c**) UNC-51 KD::mScarlet colocalizes with RPM-1 LD::GFP at axon tips (arrow). **d** Zoom-in on region highlighted in **c** (dashed box) showing UNC-51 KD::mScarlet and RPM-1 LD::GFP colocalize at axon tips. Scale bar 2.5 μm

We observed that mScarlet::UNC-51 CRISPR is increased at tips of ALM and PLM axons that fail to terminate properly (Fig. 5b, c; Supplementary Fig. 7). Such precise subcellular localization of UNC-51 is reminiscent of transgenic RPM-1::GFP at normal axon termination sites (Fig. 6a)^41,51^. Like transgenic RPM-1::GFP, we found that RPM-1::GFP CRISPR also localized to both ALM and PLM axon termination sites (Supplementary Fig. 9). Therefore, we tested if RPM-1 and UNC-51 colocalize in similar axonal compartments.

We favored a transgenic approach to these experiments for several reasons. Importantly, this ensured both RPM-1 and UNC-51 were only expressed in mechanosensory neurons. Second, mScarlet::UNC-51 CRISPR was present at extremely low levels at axon termination sites in wt animals (Fig. 5b), which was not sufficient for our experiments. Finally, because proteomic and biochemical experiments showed that RPM-1 LD interacts better with UNC-51 (Fig. 1d, f), we utilized RPM-1 LD for analysis of colocalization with UNC-51. Similar to RPM-1::GFP, RPM-1 LD::GFP accumulated in puncta at axon termination sites of PLM mechanosensory neurons, and was largely absent from axon shafts (Fig. 6b).

Because of overexpression phenotypes (Fig. 4d, e; Supplementary Fig. 5c, d) and effects on axon guidance (not shown), it was challenging to consistently visualize mScarlet::UNC-51 at normal axon termination sites in mechanosensory neurons. Therefore, we expressed UNC-51 KD. mScarlet::UNC-51 KD specifically localized to puncta at the terminated axon tips of PLM neurons (Fig. 6c). At this subcellular location, we observed colocalization between mScarlet::UNC-51 KD and RPM-1 LD::GFP (Fig. 6c, d). This result is consistent with RPM-1 regulating UNC-51 locally in specific axonal compartments, and RPM-1 inhibiting UNC-51 to affect axon termination.

### Impairing autophagy suppresses defects caused by *rpm-1* loss of function

*C. elegans* UNC-51 and its mammalian orthologs ULK1 and ULK2 are conserved kinases that are primary initiators of autophagy, but also have autophagy-independent functions in axon guidance^24^. In *C. elegans*, UNC-51 regulates axon guidance by binding to the UNC-14 RUN domain protein to affect Netrin receptor trafficking^26,27^. Thus, there are two likely models for how RPM-1 inhibition of UNC-51 might affect axon termination and synapse maintenance: (1) inhibition of autophagy, or (2) inhibition of axon-guidance mechanisms.

**Fig. 7:**
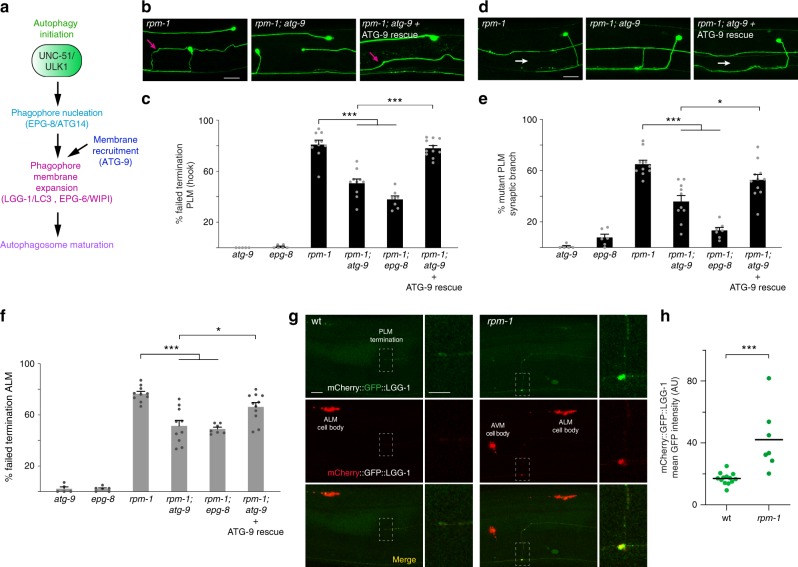
RPM-1 regulates axon termination and synapse maintenance by inhibiting autophagosome formation in axonal compartments. **a** Summary of steps in autophagy. **b** Representative images of PLM axons for indicated genotypes visualized using transgenic GFP. Severe failed termination defects (magenta arrow) in *rpm-1* mutants are suppressed in *rpm-1; atg-9* animals and rescued by ATG-9 expression in mechanosensory neurons. Scale bar 20 μm. **c** Quantitation of failed termination defects (hook) in PLM neurons for indicated genotypes. PLM termination defects are suppressed in *rpm-1; atg-9* and *rpm-1; epg-8*. **d** Representative images of PLM synaptic branch and presynaptic boutons for indicated genotypes. Synapse maintenance defects (synaptic branch retraction, white arrows) that occur in *rpm-1* mutants are suppressed in *rpm-1; atg-9* animals and rescued by expressing ATG-9 in mechanosensory neurons. Scale bar 20 μm. **e** Quantitation of synapse maintenance defects for indicated genotypes. Synapse maintenance defects are suppressed in *rpm-1; atg-9* and *rpm-1; epg-8*. **f** Quantitation of failed termination defects in ALM neurons for indicated genotypes. ALM termination defects are suppressed in *rpm-1; atg-9* and *rpm-1; epg-8*. **g** Representative images of mCherry::GFP::LGG-1/LC3 expressed in mechanosensory neurons using MosSCI. At right, zoomed-in images for highlighted regions (hatched boxes). GFP signal marks autophagosomes, and is lost in low pH autolysosomes where only mCherry signal occurs (ALM and AVM cell bodies). Low GFP signal at axon termination sites in wt animals. GFP-positive puncta accumulate at tips of axons with failed termination in *rpm-1* mutants. Scale bar 10 μm (zoom-out, left) and 5 μm (zoom-in, right). **h** Quantitation indicates GFP signal from mCherry::GFP::LGG-1 is increased at tips of axons with failed termination in *rpm-1* mutants compared with axon termination sites in wt animals. For **c**, **e**, and **f**, means are shown from 5 to 11 counts (25–35 animals/count) for each genotype. Significance determined using Student’s *t* -test with Bonferroni correction. For **h**, means are shown from seven or more animals imaged during three independent experiments for each genotype. Significance determined using Student’s *t* -test. *p < 0.05; ***p < 0.001. Source data are provided as a Source Data file

To further test the autophagy model, we evaluated whether axon termination and synapse maintenance defects in *rpm-1* mutants are affected by loss of function in other autophagy proteins (Fig. 7a). We began with *atg-9* which is required for phagophore membrane recruitment. Failed termination defects in PLM neurons (Fig. 7b, c), synapse maintenance defects in PLM neurons (Fig. 7d, e), and failed termination defects in ALM neurons (Fig. 7f) were all suppressed in *rpm-1; atg-9* double mutants. Suppression in *rpm-1; atg-9* double mutants were rescued by MosSCI expression of ATG-9 in mechanosensory neurons (Fig. 7b–f). Next, we tested *epg-8/Atg-14* which affects phagophore nucleation (Fig. 7a). *rpm-1; epg-8* double mutants showed suppression for all PLM and ALM phenotypes evaluated (Fig. 7c, e, f). A second mutant affecting phagophore nucleation, *bec-1*, was also tested (Fig. 7a). In *rpm-1; bec-1* double mutants, we observed suppression of PLM synapse maintenance defects (Supplementary Fig. 10b) and failed termination defects in ALM neurons (Supplementary Fig. 10c). However, PLM termination defects were not suppressed in *rpm-1; bec-1* animals (Supplementary Fig. 10a). Finally, we tested *epg-6/Wipi*, which affects phagophore expansion (Fig. 7a). Similar to *rpm-1; bec-1* animals, *rpm-1; epg-6* double mutants showed suppression of PLM synapse maintenance defects (Supplementary Fig. 10b) and ALM termination defects (Supplementary Fig. 10c), but not PLM termination defects (Supplementary Fig. 10a).

Several considerations emerge from these observations. Our results showing suppression occurs in *rpm-1* double mutants with numerous autophagy mutants, including *epg-8/Atg14*, *bec-1*, *atg-9* and *epg-6/Wipi* (Fig. 7b–f; Supplementary Fig. 10) are similar to findings with *rpm-1; unc-51* double mutants (Figs. 2, 3). Suppression of *rpm-1* (lf) by *epg-8*, *bec-1*, *atg-9*, and *epg-6* (lf) provides genetic evidence consistent with RPM-1 inhibition of UNC-51 influencing autophagy. All defects caused by *rpm-1* (lf) were not suppressed as strongly in *rpm-1* double mutants with *epg-8*, *bec-1*, *atg-9* and *epg-6* as *rpm-1; unc-51* double mutants. This could be because UNC-51 is the primary initiator of autophagy, and impairing initiation of autophagy gives stronger suppression of *rpm-1* than impairing downstream steps in autophagy. Alternatively, the alleles we used for *atg-9*, *epg-8*, and *epg-6* are potentially hypomorphic, and may result in weaker loss of function than the *unc-51* allele used. While the *bec-1* allele was a deletion and likely null, viability issues meant it could only be evaluated in the F1 generation when maternal rescue occurs. This could limit *bec-1* loss-of-function effects. Similar reasoning could explain why suppression of all phenotypes occurred in *rpm-1; atg-9* and *rpm-1; epg-8* double mutants, while *rpm-1; bec-1* and *rpm-1; epg-6* double mutants suppressed only two of three phenotypes evaluated. Alternatively, differences observed could be because some phenotypes are less sensitive to certain autophagy mutants.

All our results, thus far, support the model that RPM-1 inhibits UNC-51 to influence autophagy. Nonetheless, we thoroughly tested the alternative model that inhibition of UNC-51 affects axon guidance mechanisms, such as UNC-14 and Netrin signaling. As shown earlier (Figs. 2e, f, 3c), axon termination and synapse maintenance defects were not suppressed in *rpm-1; unc-14* double mutants compared with *rpm-1* single mutants. Further, MosSCI expression of UNC-51 DN suppresses *rpm-1* (lf) (Fig. 3d–g; Supplementary Fig. 3) without affecting axon guidance (Supplementary Fig. 4). To further test this second model, we evaluated how axon termination and synapse maintenance defects caused by *rpm-1* (lf) are affected in double mutants of *rpm-1* with the Netrin receptors *unc-5* and *unc-40*. Similar to outcomes with *unc-14*, axon termination and synapse maintenance defects were not suppressed in *rpm-1; unc-5* or *rpm-1; unc-40* double mutants (Supplementary Fig. 11a–c). To the contrary, *rpm-1; unc-40* double mutants displayed an increased frequency of synapse maintenance defects in PLM neurons and axon termination defects in ALM neurons (Supplementary Fig. 11b, c). These results indicate that RPM-1 function does not inhibit Netrin receptor signaling, but rather can function in parallel with Netrin signaling to regulate axon termination and synapse formation in some cellular contexts.

Overall, we now provide further genetic outcomes suggesting that RPM-1 inhibition of UNC-51 affects axon termination and synapse maintenance by restricting autophagy in mechanosensory neurons. Genetic results with *unc-5* and *unc-40* Netrin receptor mutants (Supplementary Fig. 11) combined with earlier findings using UNC-51 DN (Fig. 3d–g; Supplementary Fig. 3) and *unc-14* mutants (Figs. 2, 3) provide multiple independent observations that are not consistent with RPM-1 inhibition of UNC-51 affecting axon guidance in mechanosensory neurons.

### Autophagosome formation is restricted by RPM-1

Our results indicated that RPM-1 inhibits autophagy by restricting UNC-51 in a specific subcellular compartment, the axon termination site. To directly assess if RPM-1 affects autophagosomes, we used a pH-sensitive autophagosome marker, mCherry::GFP::LGG-1/LC3. This construct accurately differentiates between autophagosomes and autolysosomes in a range of tissues, including neurons, in *C. elegans*^52,53^. When LGG-1 is associated with autophagosomes, both GFP and mCherry fluorescent LGG-1 signals are detected. In contrast, when LGG-1 is associated with low pH autolysosomes only mCherry LGG-1 fluorescence is detected because GFP does not fluoresce at low pH.

We used MosSCI to generate animals with integrated single copy transgenes that express mCherry::GFP::LGG-1/LC3 in mechanosensory neurons. In wt animals, we observed low levels of both GFP and mCherry LGG-1 signal in puncta at axon termination sites in PLM neurons (Fig. 7g). Thus, low levels of autophagosomes are present in this subcellular compartment in adult animals. Consistent with autolysosomes accumulating in cell bodies, we observed many puncta with only mCherry LGG-1 signal in the cell bodies of ALM (Fig. 7g) and PLM neurons (not shown). These results show that there are very few autophagosomes at axon termination sites, and many LGG-1/LC3-positive autolysosomes in the cell bodies of mechanosensory neurons in adult wt animals. Next, we evaluated mCherry::GFP::LGG-1 in PLM neurons of *rpm-1* mutants (Fig. 7g). We observed a significant increase in LGG-1 puncta displaying both GFP and mCherry signal at the tip of axons that failed to terminate properly (Fig. 7g, h). This indicates that RPM-1 spatially restricts autophagosome formation at axon termination sites.

**Fig. 8:**
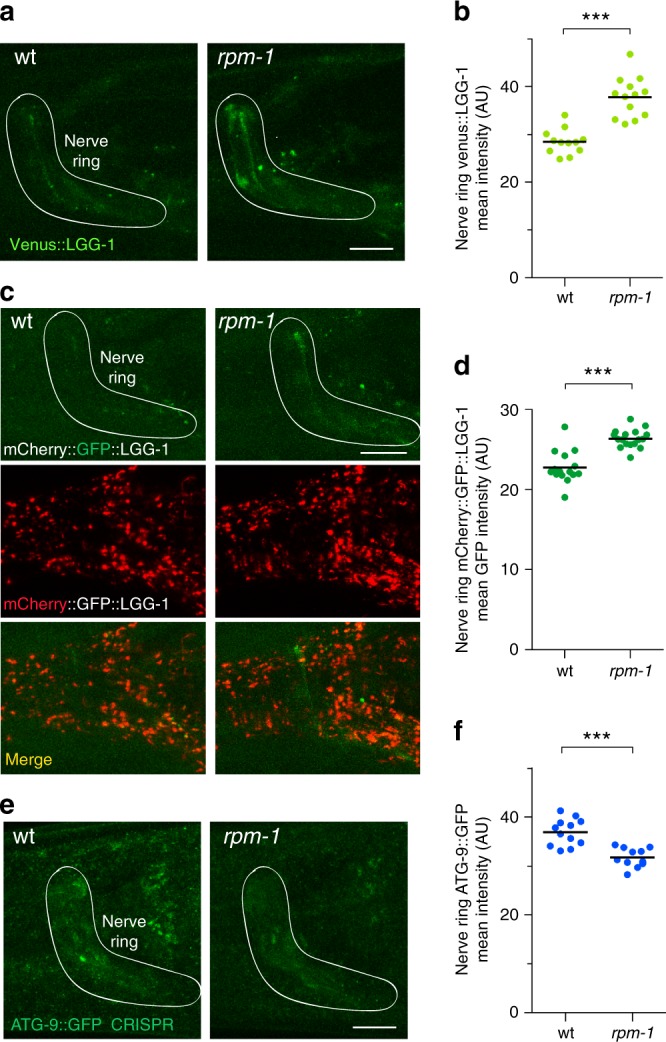
RPM-1 inhibits autophagosome formation in the nerve ring. **a** Representative images of Venus::LGG-1/LC3 expressed pan-neuronally using MosSCI. LGG-1 labels autophagosomes in wt and *rpm-1* mutants. The nerve ring is highlighted. **b** Quantitation shows Venus::LGG-1 is increased in the nerve ring of *rpm-1* mutants. **c** Representative images of GFP::mCherry::LGG-1/LC3 expressed pan-neuronally using MosSCI. GFP signal marks autophagosomes, but is lost in low pH of autolysosomes where only mCherry signal is present. **d** Quantitation indicates GFP signal from GFP::mCherry::LGG-1 in autophagosomes is increased in the nerve ring of *rpm-1* mutants. **e** Representative images of ATG-9::GFP CRISPR. ATG-9 facilitates membrane recruitment into autophagosomes. Consistent with increased autophagosome formation, there is less ATG-9::GFP in the nerve ring of *rpm-1* mutants. **f** Quantitation shows ATG-9::GFP CRISPR is reduced in the nerve ring of *rpm-1* mutants. For **b, d** and **f**, means are shown from 12 or more animals imaged during three independent experiments for each genotype. Significance determined using Student’s *t* test with Bonferroni correction. ***p < 0.001. Scale bars 10 μM. Source data are provided as a Source Data file

Because RPM-1 restricted UNC-51 broadly across the nervous system (Fig. 5e, f), we deployed a series of reagents to examine autophagosomes in the nerve ring. We evaluated a previously generated MosSCI transgene that uses a pan-neuronal promoter to express Venus::LGG-1/LC3^54^. We generated a MosSCI transgene that pan-neuronally expresses mCherry::GFP::LGG-1/LC3. Finally, we evaluated a strain in which GFP was CRISPR engineered into the *atg-9* locus^19^. We observed significant increases in Venus::LGG-1 levels in nerve rings of *rpm-1* mutants compared with wt animals (Fig. 8a, b). Likewise, GFP signal derived from mCherry::GFP::LGG-1 was significantly increased in nerve rings of *rpm-1* mutants (Fig. 8c, d). ATG-9::GFP levels were lower in the nerve ring of *rpm-1* mutants compared with wt animals (Fig. 8e, f). While a reduction in ATG-9 might seem counterintuitive, previous studies showed that reduced ULK function in mammalian cells and yeast caused increased ATG9 accumulation^55,56^. Similarly, *unc-51* mutants display strong ATG-9::GFP accumulation in embryos^57^. Consistent with these prior findings, we observed the inverse result with reduced ATG-9 accumulation in the nerve ring (Fig. 8e, f) when UNC-51 is increased in *rpm-1* mutants (Fig. 5e, f).

Collectively, these results support two findings. First, RPM-1 inhibits formation of autophagosomes in subcellular axonal compartments corresponding to locations where it restricts UNC-51. Second, RPM-1 inhibits autophagosome formation broadly across the nervous system, similar to its widespread effect on UNC-51.

## Discussion

**Fig. 9:**
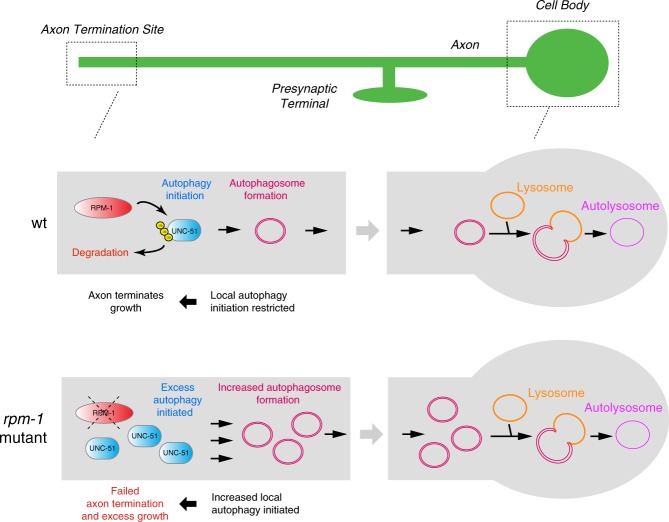
RPM-1 ubiquitin ligase activity spatially restricts UNC-51 to inhibit initiation of autophagy in neurons. Summary showing RPM-1 ubiquitin ligase activity restricts the autophagy initiating kinase UNC-51 and autophagosome formation at axon termination sites in vivo. Autophagosomes are transported back to the cell body where they form autolysosomes. In neurons lacking *rpm-1*, UNC-51 levels are elevated in specific axonal compartments. This leads to excess autophagy initiation, increased autophagosome formation, and failed axon termination

Autophagy is a degradative process that influences cellular health and disease. Neurons are postmitotic cells with high-energy demands, have particular cellular anatomy, and need to maintain axon architecture and synapses over a lifetime. As a result, neurons face unique challenges in managing autophagy. Abnormal autophagy is also a hallmark of many neurodegenerative diseases. Thus, understanding the underpinnings of how autophagy is regulated in the nervous system is of the utmost importance. We now show that the ubiquitin ligase RPM-1 restricts the autophagy initiating kinase UNC-51/ULK in neurons to promote axon termination and synapse maintenance (Fig. 9). By inhibiting UNC-51, RPM-1 restrains initiation of autophagy and autophagosome formation in subcellular axonal compartments. Effects of RPM-1 on UNC-51 and autophagy occur across the nervous system, suggesting RPM-1 is a broad, prominent mechanism for inhibiting initiation of autophagy in neurons. Cell-based results indicate that the human RPM-1 ortholog, PAM, has conserved effects on ULK1 ubiquitination and proteins levels. Our findings provide the first evidence that ubiquitin ligase activity inhibits UNC-51/ULK and autophagy initiation in the nervous system.

The ULK kinase acts as a primary, conserved initiator of autophagy^24^. Studies in nonneuronal cells have shown ULK is restricted by mTOR phosphorylation^58–60^. Whether mTOR inhibits ULK in neurons during development or neurodegenerative disease remains controversial^6,23^. This is likely because other inhibitors of ULK in the nervous system remain to be identified. Our results show that the ubiquitin ligase RPM-1 negatively regulates the *C. elegans* ULK ortholog UNC-51 to influence axon termination, synapse maintenance, and behavioral habituation (Fig. 9). Unbiased, affinity-purification proteomics and biochemistry from *C. elegans* demonstrated that an RPM-1 ubiquitin ligase “trap”, expressed exclusively in the nervous system, enriches UNC-51 (Fig. 1). Multiple, independent genetic loss-of-function approaches, rescue experiments, and transgenic overexpression studies indicated that RPM-1 inhibits UNC-51 in the mechanosensory neurons of *C. elegans* (Figs. 2–4). Finally, mScarlet::UNC-51 CRISPR levels were increased in subcellular axonal compartments and across the nervous system of *rpm-1* (lf) mutants, or when RPM-1 ubiquitin ligase activity was eliminated using CRISPR gene editing (Fig. 5). qPCR results ruled out effects of RPM-1 on *unc-51* mRNA transcription (Fig. 5g; Supplementary Fig. 8). These findings demonstrate that RPM-1 inhibits UNC-51 via its ubiquitin ligase activity, spatially regulates the stability of UNC-51 in axons, and affects UNC-51 stability broadly across the nervous system.

Our results are consistent with prior studies that showed RPM-1, and orthologous PHR ubiquitin ligases in flies and mammals, trigger substrate degradation^29^. In the case of axon and synapse development, *C. elegans* RPM-1, Drosophila Highwire, and mouse Phr1 regulate stability of the MAP3K DLK-1^34,61,62^ and the tuberous sclerosis complex^63–65^. Postdevelopmentally, Highwire and mouse Phr1 degrade NMNAT to trigger axon degeneration^31,66,67^. Here, we show RPM-1 ubiquitin ligase activity spatially restricts UNC-51 to facilitate axon termination and synapse maintenance (Figs 2–5). Cell-based results demonstrate that the human PHR protein PAM restricts ULK1 protein levels (Fig. 5h, i), and is sufficient to ubiquitinate ULK1 in a proteasome-dependent manner (Fig. 5j). This suggests that RPM-1 could be a conserved mechanism that inhibits UNC-51. Whether PHR proteins restrict ULK to impact axon development, synapse maintenance or axon degeneration in other organisms remains an intriguing question.

Previous work in nonneuronal cells has revealed two examples of ubiquitin ligase activity influencing ULK. TRAF6 monoubiquitinates and positively regulates ULK1^68^, while KLHL20 can degrade ULK1 in HeLa cells and muscles^28^. Our findings now provide the first example of a ubiquitin ligase that restricts UNC-51/ULK in a subcellular compartment, spatially restricts ULK in neurons, and inhibits ULK in the nervous system. Future experiments will be needed to test whether PHR proteins function alone, or in a ubiquitin ligase network with TRAF6 and KLHL20 to regulate ULK in the nervous system.

Cells utilize two major processes for protein degradation. Generalized turnover of cellular components by autophagy, and ubiquitin ligases that target specific proteins for degradation by the proteasome. It remains unknown if ubiquitin ligase activity affects autophagy in neurons or in the nervous system in vivo. Moreover, how initiation of autophagy is inhibited in the nervous system remains poorly understood. Here, we reveal an important link between ubiquitin ligase activity and neuronal autophagy by showing that the RPM-1 ubiquitin ligase restricts the autophagy initiating kinase UNC-51/ULK to inhibit autophagy (Fig. 9). Importantly, we provide several independent pieces of evidence that show RPM-1 inhibits autophagy, aside from effects on UNC-51. We observed suppression of defects in axon termination and synapse maintenance caused by *rpm-1* (lf) with multiple autophagy mutants, including *atg-9*, *epg-8/Atg14*, *bec-1*, and *epg-6/Wipi* (Fig. 7b–f; Supplementary Fig. 10). Autophagosome markers were increased in subcellular axonal compartments in *rpm-1* mutants (Fig. 7g, h). Furthermore, *rpm-1* mutants showed significant changes in multiple autophagosome markers in the nerve ring, a proxy for effects across the nervous system (Fig. 8). Thus, outcomes with multiple autophagy markers, different functional contexts and numerous autophagy mutants demonstrate that RPM-1 restricts autophagy. These findings, taken together with effects of RPM-1 on UNC-51, indicate that RPM-1 inhibits autophagy initiation in neurons, spatially restricts autophagy in axonal compartments, and influences autophagy across the nervous system (Fig. 9).

While our findings in *C. elegans* demonstrate that RPM-1 inhibits autophagy, previous work on the Drosophila neuromuscular junction identified a different link between an RPM-1 ortholog and autophagy^18,69^. These studies showed that autophagy can degrade the fly PHR protein Highwire, but did not test whether *Unc-51/Ulk* suppresses *Hiw* or examine autophagy in *Hiw* mutants. Nonetheless, a simple model merges our new findings with this prior work: if PHR proteins inhibit initiation of autophagy (as our findings indicate), it is plausible feedback occurs via autophagic degradation of PHR proteins. This might create a developmental switch between low PHR function and high autophagy when growth is happening, and elevated PHR activity and reduced autophagy when axon growth terminates.

While we focus on how RPM-1 inhibits UNC-51 and autophagy to influence axon termination and synapse maintenance, it is worth considering the broader implications of our findings. For instance, both ULK^24^ and the ubiquitin ligase complex formed by PAM/MYCBP2^70–72^ are associated with some cancers. This suggests PAM inhibition of ULK and autophagy could influence oncogenesis. Autophagy also plays a prominent role in many neurodegenerative diseases, including Parkinson’s and Alzheimer’s disease^5,6,23^. It has been proposed that increased autophagy could be protective for neurodegenerative disease. If this is the case, our results suggest the human PHR protein PAM might be a possible molecular target for increasing autophagy and slowing the onset or progression of disease.

## Methods

### Genetics and strains

*C. elegans* N2 isolate was used for all experiments, and worms were maintained using standard procedures. The following mutant alleles were used: *rpm-1* (*ju44*), *unc-51* (*e369*), *unc-14* (*e57*), *atg-9* (*bp564*), *epg-6* (*bp242*), *epg-8 (bp251)*, *bec-1(ok691), unc-5* (*e53*), *unc-40* (*e271*), and *glo-1* (*zu391*). *bec-1(ok691)* was maintained with the balancer nt1[qIs51] (IV;V). Homozygous F1s were analyzed, because lethality was problematic in the F2 generation. The following integrated transgenes were used: *muIs32* [P_*mec-7*_GFP]; *jsIs973* [P_*mec-7*_mRFP]; *bggIs9* [P_*rpm-1*_GS::RPM-1]; *bggIs19* [P_*rpm-1*_GS::RPM-1 LD]; *bggIs23* [P_*rpm-1*_GS::GFP]; *bggIs34* [P_*mec-3*_RPM-1::GFP]; *bggIs44* [P_*mec-3*_RPM-1 LD::GFP]. The following MosSCI transgenes were used: *uwaSi6* [P_*rab-3*_ oxCerulean::oxVenus::LGG-1]; *bggSi4* [P_*mec-7*_UNC-51 *K39I*]; *bggSi9* [P_*mec-7*_ATG-9]*; bggSi10* [P_*mec-7*_ATG-9]; *bggSi14* [P_*mec-7*_mCherry::GFP::LGG-1]; *bggSi16* [P_*rab-3*_ mCherry::GFP::LGG-1]; and *bggSi20* [P_*mec-7*_UNC-51]. All MosSCI transgenes were inserted into *ttTi5605* on LG II. The following CRISPR alleles were used: *ola274* [ATG-9::GFP CRISPR]; *bgg6* [RPM-1::GFP CRISPR]; *bgg6 bgg39* [RPM-1 LD::GFP CRISPR]; *bgg6 bgg40* [RPM-1 LD::GFP CRISPR]; *bgg23* [3xFLAG::UNC-51 CRISPR]; and *bgg18* [mScarlet::UNC-51 CRISPR]. Transgenes and CRISPR alleles used for specific experiments are described in Supplementary Table 2. All mutants and transgenic lines were outcrossed four or more times prior to experiments.

Strains were grown at 23 °C for all analysis with the exception of *bggIs34* (P_*mec-3*_RPM-1) and *bggIs44* (P_*mec-3*_RPM-1 LD), which were grown at 20 °C to improve expression from the *mec-3* promoter.

### Molecular biology

The *unc-51* open reading frame was cloned from N2 genomic DNA with HpaI and XbaI sites flanking the start and stop codons, respectively (pBG-GY355). Point mutagenesis was used to create UNC-51 K39I (pBG-GY869), which fully impairs UNC-51 kinase activity^44,45^. This is referred to as UNC-51 DN when used for suppression experiments (i.e. *rpm-1* mutant and wt at the *unc-51* locus, Fig. 3d–g; Supplementary Fig. 3) and UNC-51 KD when used for rescue experiments (i.e. *rpm-1; unc-51* background; Fig. 4a–c; Supplementary Fig. 5a, b). For colocalization experiments (Fig. 6), a second KD version of UNC-51 was created with a K39R substitution, which has milder reductions in kinase activity^45^.

To create P_*rpm-1*_GS::RPM-1 (pBG-186), we initially generated P_*rpm-1*_RPM-1 (pBG-182) by PCR amplifying the *rpm-1* promoter with SbfI and XmaI/SacII sites from pCZ160 and subcloning into pSAM-13 (P_*mec-3*_RPM-1). The N-terminal GS tag [2xProtein G::TEV::5x streptavidin-binding peptide (SBP)] was inserted into pBG-182 at the XmaI site to generate pBG-186. P_*rpm-1*_GS::RPM-1 LD (pBG-255) was made by introducing three point mutations (C3535A, H3537A, and H3540A) into pBG-186. Site-directed mutagenesis was performed with QuikChange II XL Mutagenesis Kit (Agilent Techologies). All point mutations were confirmed by sequencing, and all mutated constructs were confirmed to be free of other mutations.

*atg-9* cDNA (pBG-GY932) was cloned from an N2 cDNA pool prepared using random hexamers and SuperScript^TM^ IV First-Strand Synthesis System (Invitrogen).

Worm codon-optimized mScarlet-I was subcloned from pMS050 (Addgene #91826). *lgg-1* genomic sequence was cloned from P_*rab-3*_ oxCerulean::oxVenus::LGG-1 (Addgene #68045).

### Transgenics

All integrated transgenes and extrachromosomal arrays used in this study were generated using standard *C. elegans* transgenic procedures. All transgene construction details and injection conditions are detailed in Supplementary Table 3. Integrated transgenic lines used for RPM-1 proteomics and colocalization experiments were generated by TMP/UV integration of extrachromosomal arrays (see Supplementary Tables 2, 3 for details). MosSCI insertion was carried out using previously described methods^73^. The Mos-targeting vector, pCFJ350, was modified to contain P_*rps-27*_NeoR from pCFJ1202 (Addgene #44484) and removed *unc-119*(+). MosSCI selection was carried out on neomycin plates (NGM supplemented with G418 (Gold Biotechnology) at 0.5 mg/ml). All MosSCI strain construction details are listed in Supplementary Table 2, and injection conditions are listed in Supplementary Table 3.

### CRISPR-Cas9 genome editing

CRISPR alleles were generated by direct injection of ribonucleoprotein complexes using co-CRISPR^74^. Injection mixes containing tracrRNA (Dharmacon), crRNA (Dharmacon), repair template (PCR or ssODN (Extremers, Eurofins), and Cas9 protein were heated at 37 °C for 15 min prior to injection. *dpy-10* or *pha-1* co-CRISPR strategies were used. All gene edits were confirmed by sequencing and outcrossed at least four times to N2. See Supplementary Table 3 for injection conditions, and Supplementary Table 4 for crRNA and repair template sequences.

### *C. elegans* proteomics

Mixed-stage animals were cultured in liquid (S complete media with HB101, shaking 185 RPM) for 2–3 days. Worms were harvested by centrifugation, separated on 30% sucrose flotation, and washed three times with 0.1 M NaCl. Packed worms were frozen as pellets in liquid N_2_. Pellets were cryomilled (Retsch) with EDTA-free protease inhibitor tablets (Roche), and lysed in four times volume lysis buffer (50 mM Tris HCl (pH 7.5) or 50 mM HEPES (pH 7.5), 150 mM NaCl, 1.5 mM MgCl_2_, 10% glycerol, detergent [0.1% NP-40, 0.3% NP40, or 0.1% CHAPS], 1 mM DTT, 1 mM PMSF, 1x Halt Protease Inhibitor Cocktail (Thermo Scientific), 1 mM sodium orthovanadate, 5 mM sodium flouride, 1 mM sodium molybdate). Protein lysates were stirred for 2 min then rotated for 5 min at 4 °C, and cleared by centrifugation at 20,000×*g* for 10 min.

Volume of lysate containing 80 mg of total protein was incubated with 500 μL of Dynabeads (M280 anti-rabbit IgG, Invitrogen) for 4 h at 4 °C. Following affinity capture, beads were washed five times with lysis buffer. Purification quality was assessed by running samples on 3–8% Tris-acetate gels (Invitrogen) and western blotting (1% sample, anti-SBP antibody (Sigma-Aldrich)) or silver staining (9% sample, Thermo Scientific). The remaining sample (90%) was run on Tris-Glycine SDS-PAGE at 120 V for 10 min. Gel was Coomassie stained for 1 h at room temperature with shaking, followed by de-staining in water overnight. Gel bands were excised and treated with 10 mM DTT followed by 50 mM iodoacetamide and subjected to in-gel trypsin digestion. Prior to mass spectrometry analysis, peptide pools were acidified, desalted through a Zip-Tip C18 column, and dried down. Samples were then resuspended in 100 μl of 0.1% formic acid, and 13 μl were loaded into mass spectrometer. Samples were analyzed using an Orbitrap Fusion™ Tribrid™ Mass Spectrometer (ThermoFisher Scientific) coupled to an EASY-nLC 1000 system. Peptides were on-line eluted on an analytical RP column (0.075 × 250 mm Acclaim PepMap RLSC nano Viper, ThermoFisher Scientific), operating at 300 nL/min using the following gradient: 5–25% B for 40 min, 25–44% B for 20 min, 44–80% B in 10 s, 80% B for 5 min, 80–5% in 10 s, and 5% B for 20 min [solvent A: 0.1% formic acid (v/v); solvent B: 0.1% formic acid (v/v), 80% CH3CN (v/v) (Fisher Scientific)].

The Orbitrap Fusion was operated in a data-dependent MS/MS mode using the ten most intense precursors detected in a survey scan from 380 to 1400 m/z performed at 120 K resolution. Tandem MS was performed by HCD fragmentation with normalized collision energy (NCE) of 30.0%. Protein identification was carried out using Mascot and Sequest algorithms, allowing Oxidation (M), and Deamination (N and Q) as variable modifications. Other settings included Carbamidomethylation of Cys as a fixed modification, three missed cleavages, and mass tolerance of 10 and 20 ppm for precursor and fragment ions, respectively. MS/MS raw files were searched against a *C. elegans* database along with human keratins and porcine trypsin.

Scaffold (Proteome Software) was used to validate MS/MS-based peptide and protein identifications. Peptide identifications were accepted if they could be established at >5.0% probability to achieve a false discovery rate (FDR) <1.0% by the Scaffold Local FDR algorithm. Protein identifications were accepted if they could be established at >98.0% probability to achieve an FDR <1.0% and contained at least two identified peptides. Protein probabilities were assigned by the Protein Prophet algorithm.

RPM-1-binding proteins were assessed using the following criteria: (1) The protein was detected in a minimum of two experiments from seven total proteomic experiments. (2) The protein had 2x or more total spectra in the GS::RPM-1 or GS::RPM-1 LD samples compared with negative control (GS::GFP). (3) Ribosomal and vitellogenin proteins were removed because they are widely regarded as proteomic contaminants.

### *C. elegans* biochemistry

For *C. elegans* biochemistry with CRISPR strains, animals were grown, processed, cryomilled, and lysed following the same parameters as discussed above for proteomics. Lysates (2.5 mg of total protein) were incubated with 3.0 μg anti-FLAG (M2 mouse monoclonal, Sigma) or 0.6 μg anti-GFP (3E6, Initrogen) antibodies for 30 min then incubated for 4 h with 10 μL of protein-G agarose (Roche) at 4 °C. Precipitated complexes were heated to 70 °C for 10 min in LDS sample buffer (Invitrogen) and run on 3–8% Tris-Acetate gels (Invitrogen). Gels were transferred overnight at 4 °C to the PVDF membranes (Millipore) and immunoblotted with either anti-FLAG (1:1000 dilution, rabbit polyclonal, Cell Signaling) or anti-GFP (1:1000 dilution mouse monoclonal, Roche) antibodies. Proteins were visualized using HRP-conjugated secondary antibodies (1:20,000 dilution; GE Healthcare Life Sciences, Fisher Scientific) and ECL (Supersignal West Femto, Thermo Scientific). Blots were imaged with X-ray film.

### Analysis of axon and synapse development

ALM axon termination, PLM axon termination, and PLM synaptic branch and presynaptic boutons were analyzed and quantified using the transgene *muIs32* (P_*mec-7*_GFP) similar to prior studies^36,43^. For imaging, young adult animals were anesthetized in 5 µM levamisole in M9 buffer on a 2% agar pad on glass slides. Coverslips were applied, and slides were mounted and visualized with a Leica DM5000 B (CTR5000) epifluorescent microscope (×40 or ×63 oil-immersion objectives), or a Leica SP8 confocal microscope (×25 or ×40 objectives). For confocal microscopy, coverslips were sealed with BIOTIUM CoverGrip Coverslip Sealant.

For ALM neurons, failed termination was defined as axons that grew beyond the normal termination point just prior to the animal’s nose, and had a short or large hook. Premature termination was defined as axons that stopped before the normal termination site. For PLM neurons, failed termination was scored as “hook” (severe axon termination phenotype) or “overextension” (moderate axon termination phenotype). Premature termination of PLM neurons, which occurred in *unc-51* and *rpm-1; unc-51* double mutants, was defined as stopping at or before the vulva in accordance with prior work^41^.

Synaptic branches were scored as a proxy for synapse formation in the PLM neurons as demonstrated previously^36,43^. Prior work showed synaptic branch retract in *rpm-1* mutants is due to failed synapse maintenance^46^. Synaptic branches with presynaptic varicosities were scored as wild-type. The absence of terminal varicosities, incomplete branches, or total absence of branches was scored as mutant. In the case of *unc-51* mutants, we at times observed multiple collateral PLM branches. If at least one complete branch reached the VNC to form presynaptic varicosities the neuron was not considered mutant. If the branch occurred significantly prior to the PVM neuron, it was scored as mutant.

### Tap habituation

Experiments were performed as described previously with minor modifications^49^. Briefly, age-synchronized animals (~50–100) were cultivated from egg until gravid adult (72–80 h) at 23 °C on 5 cm NGM plates with 100 μl of *E. coli* (OP50) spread evenly across surface. All strains contained the *muIs32* transgene. Using Multi-Worm Tracker, animal behavior was recorded for 550 s on cultivation plates. After the first 100 s, 45 tap stimuli were given with a 10 s interstimulus interval. Response to tap was measured by reversal probability (the fraction of animals that reversed their locomotion within 2 s of the tap). For each plate, exponential curves were fit to the responses across stimuli, and habituation level was measured as the value of the fit at the final stimulus.

### Image analysis

For quantitative analysis of fluorescent protein intensity, images were collected using identical confocal microscopy settings across genotypes and experiments. Leica Application Suite (LAS) software was used to define Z-stacks that were collected at 0.9–1.5 μm intervals. Leica image files were processed and analyzed using Fiji/Image J software. Any adjustments to overall image brightness were consistent across genotypes for all experiments.

For ALM and PLM analysis with mScarlet::UNC-51 CRISPR and mCherry::GFP::LGG-1 (only GFP signal analyzed), a region of interest (ROI) from a single confocal slice was defined as a 6–9 µm^2^ region at the axon termination site for a given genotype. Only axons where the termination site could be confidently visualized were used for analysis. This was particularly important for *rpm-1* and *rpm-1* LD CRISPR animals that have failed termination defects.

For nerve ring analysis of mScarlet::UNC-51, Venus::LGG-1, mCherry::GFP::LGG-1 (GFP signal) and ATG-9::GFP, maximum intensity projections were made. ROIs were defined manually by anatomical location within the head. If available for a given strain, the anatomy of the ALM mechanosensory neuron, imaged in a separate channel, was used to help define the ROI. ROI area was 300–400 µm^2^. Average areas for all genotypes fell within a small range of 320–345 µm^2^.

### qPCR

RNA was isolated from young adult animals. Worms were homogenized, and RNA was extracted with Trizol reagent (Invitrogen) using repeated cycles of vortexing (4 °C) with 500- µm glass beads (Sigma) and freeze-thawing in liquid N_2_. RNA was isolated with chloroform (Sigma) and acid phenol:chloroform:Isoamyl alcohol (125:24:1), pH 4.5 (Ambion). Contaminating DNA was removed by subjecting 10 µg of RNA to DNA-free Kit (Ambion). cDNA was generated from 1 µg of RNA with Superscript IV Vilo (Invitrogen). Quantitative PCR was carried out using Power-Up Sybr Green Master Mix in the ABI 7900 cycler (Applied Biosystems). All qPCR amplifications were performed in triplicate using cDNA and no RT negative controls. Quantitation of *unc-51* was normalized to *tba-1*, *pmp-3*, and *Y45F10D.4* reference genes^75^ using the comparative C(T) method for analysis^76^. All primers used for qPCR are listed in Table S5.

### SH-SY5Y cell culture, lentiviral infection, and biochemistry

SH-SY5Y neuroblastoma cells and HEK 293T cells (purchased from American Type Culture Collection) were maintained in the EMEM and DMEM, respectively. Media was supplemented with 10% FBS and penicillin/streptomycin. Cells were incubated at 37 °C, 5% CO_2_, and 95% humidity. The *PAM* shRNA oligonucleotide sequence was: CCGGATTATGCAAGGATGGATTATACTCGAGTATAATCCATCCTTGCATAATTTTTTG (Sigma, catalog number TRCN0000330582). Lentiviruses were generated with 293 cells using the MISSION Packaging System (Sigma). SH-SY5Y were infected for 8 h with lentivirus expressing *PAM* shRNA or nucleotide control sequence. Infected cells were allowed to recover overnight in new media and then selected for 5–7 days in media containing puromycin (1 μg/ml). Following puromycin selection, cells were treated with DMSO, or cycloheximide for 1–4 h and harvested for biochemistry.

Cells were lysed at 4 °C in RIPA buffer (25 mM Tris HCl pH 7.6, 150 mM NaCl, 1% NP-40, 1% sodium deoxycholate, 0.1% SDS, Thermoscientific) containing protease and phosphatase inhibitors (Roche) and centrifuged at 14,000×*g* for 15 min at 4 °C. Protein concentrations were determined using BCA protein assay (Pierce - Thermo Scientific). For all samples, 25 μg of the total protein was run on NuPAGE Novex 4 to 12% Bis-Tris polyacrylamide gels (Life Technologies) and transferred to nitrocellulose membranes (0.45 -μm pore size) using semidry transfer and Trans-Blot transfer medium (Bio-Rad, Hercules, CA). Membranes were blocked in Odyssey blocking buffer (LI-COR Biosciences) and incubated overnight at 4 °C with primary antibodies. After repeated washes with TBS-T [20 mM tris (pH 7.6), 140 mM NaCl, and 0.1% Tween 20], blots were incubated with the appropriate IRDye-conjugated secondary antibody (LI-COR Biosciences) and imaged using the LI-COR Odyssey. For blotting PAM, which is extremely high-molecular-weight, samples were run on NuPAGE 3–8% Tris-Acetate gels and wet transfer was done overnight (25 V for 16 h) in TAE transfer buffer at 4 °C. The following primary antibodies were used: anti-GAPDH (Millipore), anti-PAM (Bethyl Laboratories), anti-ULK1 (Cell Signaling Technologies).

### ULK1 ubiquitination

Ubiquitination assays were performed as described previously using HEK 293T cells^31^. 293T cells were transfected with 0.5 μg of HA-Ubiquitin (pBG-325), 1–4 μg of MYC-ULK1 (pBG-394), or MYC control plasmid, and 1.5–4 μg of GFP-PAM (pBG-GY765) or GFP control plasmid. Twenty-two to twenty-four hours post transfection, cells were treated with DMSO or MG132 (50 μM) for 4 h. Cells were then lysed using 1% Triton-RIPA buffer (50 mM Tris, pH 7.5, 150 mM NaCl, 1 mM EDTA, 1% Triton X-100, 1% sodium deoxycholate, 0.1% SDS, 5 mM N-ethylmaleimide, phosphatase inhibitors (Halt, Pierce), and protease inhibitors (Halt, Pierce)). Anti-MYC immunoprecipitations (9E10 mouse monoclonal) were performed using 0.5 mg of the total protein lysate. In all, 75% of immunoprecipitates were run on a 3–8% Tris-Acetate gradient gel (Invitrogen) and immunoblotted for HA-ubiquitin. In total, 25% of the IPs were run to assess MYC-ULK1 IP inputs. Expression of MYC-ULK1 and GFP-PAM were observed by immunoblotting 12.5 μg of whole-cell lysate (WCL). Immunoblotting for ERK was used as a nontransfected loading control. The following primary antibodies were diluted 1:1000 in 5% nonfat milk in TBST and applied to blots overnight at 4 °C. (1) Mouse monoclonal anti-GFP antibodies (Roche Applied Science); (2) mouse monoclonal 9B11 anti-MYC (Sigma); (3) mouse monoclonal 6E2 anti-HA antibody (Cell Signaling); (4) rabbit polyclonal K23 anti-ERK-1 (Santa Cruz Biotechnology). Blots were visualized with HRP-conjugated anti-mouse (GE Healthcare), or anti-rabbit (ThermoFisher Scientific) secondary antibodies. Secondaries were detected using enhanced chemiluminescent reagent (1:2 or 1:5 dilution of Supersignal Femto (ThermoFisher Scientific) in TBS), and visualized with X-ray film.

### Statistical analysis

*Proteomics*: For identification of proteins in RPM-1 and RPM-1 LD samples versus GFP control samples, as well as RPM-1 versus RPM-1 LD samples, an unpaired, two-tailed Student’s *t* test was used to assess significance using the total spectral counts from seven independent proteomic experiments. Each experiment was considered an individual *n* for analysis. The total peptide spectra were normalized to molecular weight to avoid bias caused by protein size. All samples were blinded for genotype during mass spectrometry.

*Axon and synapse analysis*: For analysis of ALM axon termination, PLM axon termination and PLM synapse maintenance, statistical comparisons were done with an unpaired, two-tailed Student’s *t* test and Bonferroni correction. Error bars are SEM. Significance was defined as *p* < 0.05. Bar graphs represent averages from 4 to 10 counts (25–35 neurons/count) for each genotype from three or more independent experiments. Dots in plots represent averages from single-counting sessions.

*Tap habituation*: Reversal probabilities and habituation level were measured for 16 plates (~50–100 animals per plate) for all genotypes (*n* = 16). The data were collected on four independent days (four plates per genotype per day). The data presented represent mean ± SEM. Exponential fits in graph are for the combined data. Habituation-level differences were assessed using Student’s *t* tests with Bonferroni correction.

*mScarlet::UNC-51 and autophagy marker analysis*: For analysis of fluorescence intensity for mScarlet::UNC-51 (Fig. 5c, f), means were calculated from 12 or more animals imaged from 3 to 4 independent experiments for each genotype. For autophagy marker in Fig. 7h, means were calculated from seven or more animals imaged from 3 to 4 independent experiments for each genotype. For all autophagy markers in Fig. 8, means were calculated from 12 or more animals imaged from three independent experiments for each genotype. Significance was determined by using an unpaired, two-tailed Student’s *t* test and Bonferroni correction where applicable.

*Western blot quantitation*: For analysis of ULK1 levels, we used LI-COR quantitative imaging of western blots. Mean intensity was determined from four or five independent experiments. Statistical comparison was performed using an unpaired two-way ANOVA with a post hoc Student’s *t* test.

### Reporting summary

Further information on research design is available in the Nature Research Reporting Summary linked to this article.

## Supplementary information

Supplementary Information

Peer Review

Reporting Summary

Source Data

## Data Availability

All data and reagents are freely available and will be shared upon request. Further details about raw data for all quantitative experiments can be found in the Reporting Summary and in the Source Data File that accompanies this study (see Supplementary Materials).
