# Supplementary material for: Autophagy is inhibited by ubiquitin ligase activity in the nervous system
Source: Nat Commun. 2019 Nov 1;10:5017. doi: 10.1038/s41467-019-12804-3 (PMC6825199; doi:10.1038/s41467-019-12804-3)
Supplement: Supplementary file 1 — Supplementary Information [file 41467_2019_12804_MOESM1_ESM.pdf]

Autophagy is Inhibited by Ubiquitin Ligase Activity in the Nervous System

Wang *et al.*

SUPPLEMENTARY FIGURES AND TABLES

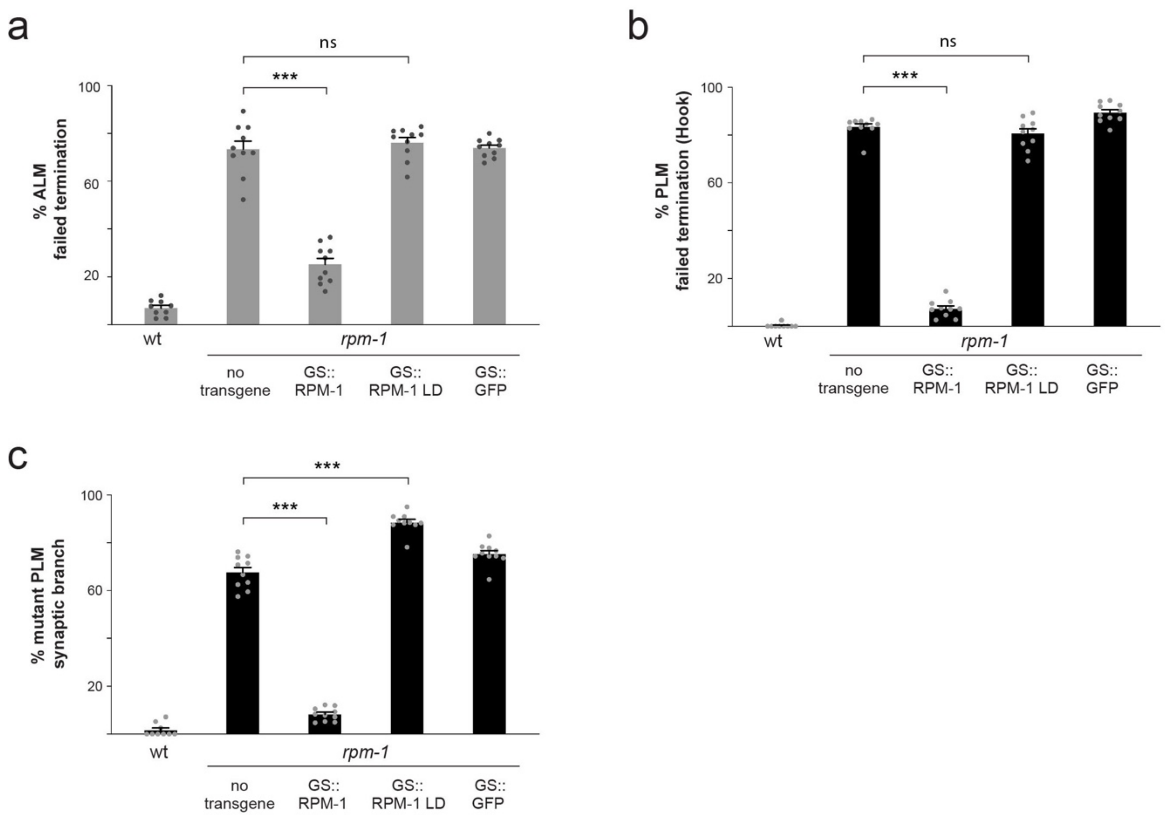

Supplementary Figure 1. Functional validation of RPM-1 proteomic reagents. Integrated transgenic lines that use native *rpm-1* promoter to express GS::RPM-1, GS::RPM-1 LD or GS::GFP (negative control) were evaluated for rescue of *rpm-1* mutant phenotypes. (**a** and **b**) Quantitation of failed termination defects in (**a**) ALM and (**b**) PLM mechanosensory neurons. (**c**) Quantitation of synapse maintenance defects in PLM neurons for indicated genotypes. Note GS::RPM-1 rescues *rpm-1* (lf), but GS::RPM-1 LD which lacks ubiquitin ligase activity fails to rescue. Means are shown from 9 or more counts (25-35 animals/count) and error bars represent SEM. Significance determined using student's *t*-test with Bonferroni correction. *** $p < 0.001$ and ns = not significant. Source data are provided as a Source Data file.

```
MEQFDGFEYSKRDLLGHGAFAIVYRGRYVDRTDVPVAIKAIAKKNISKSKNLLTKEIKILKELSSLKHENLVGLLKCTETPTHVYLVMEFCN
GGDLADYLQQKTTLNEDTIQHFVVQIAHALEAINKKGIVHRDLKPQNILLCNNSRTQNPHFTDIVIKLADFGFARFLNDGVMAATLCGSPMY
MAPEVIMSMQYDAKADLWSIGTILFQCLTGKAPFVAQTPPQLKAYYEKTRELRPNIPEWCSPNLRDLLLRLLKRNAKDRISFEDFFNHPFLT
SPLLPSPSKRILESARSPLLANRRIITPQSSLPVPKRAGSTKLDSPTPVRRIGESPRVQRRVITPGMPSPVPGAPMQESTDFTFLPPRQESS
PVKQVQVHTNVSPSLTTCKPVPVPSQRLTYQKMEERLAAARKTAVPSSSSPTGSAVSAQHQHQHQQQQEPASSPVVQRIERPDQLPRRTTLQ
DPNAHDIERMTMPNPTFVVCGSSTKPSPNNANRVRRSTITSPADTQDMVAADQMLSNLDPTTTTTTIPKSATTANIQGIPRGARDRSVTSPP
QPTIHENEPLDNAKYQQTDVNNSPTAPTEPFIIKNQTTCSTSSTSSSVVEEEEAMSLPFASGSHLAAGFKKTPAEVPMDHGALPPALDQEIV
LGEEHKQILAKLRFVAELVDTLIHVAEQKDNPLASAMASRRQLLTTGTSTTNTSSPYRRAEQLVVYVRALHMLSSALLAQTNVANRVLHPS
VAVQQVLNQLNDKYHQCLVRSQELASLGLPGQDPAMAVISAERIMYRHAIELCQAAALDELFGNPQLCSQRYQTAYMMLHTLAEQVNCDQDK
TVLTRYKVAVEKRLRILERQGFVAAVNT
```

Supplementary Figure 2. UNC-51 peptides identified from proteomics with GS::RPM-1 LD. Shown is UNC-51 protein sequence (856 aa) and UNC-51 peptides (red and orange) identified from affinity purification proteomic experiments using GS::RPM-1 LD. Shown is cumulative UNC-51 peptide coverage from 7 independent proteomics experiments, which identified 28% of total UNC-51 sequence.

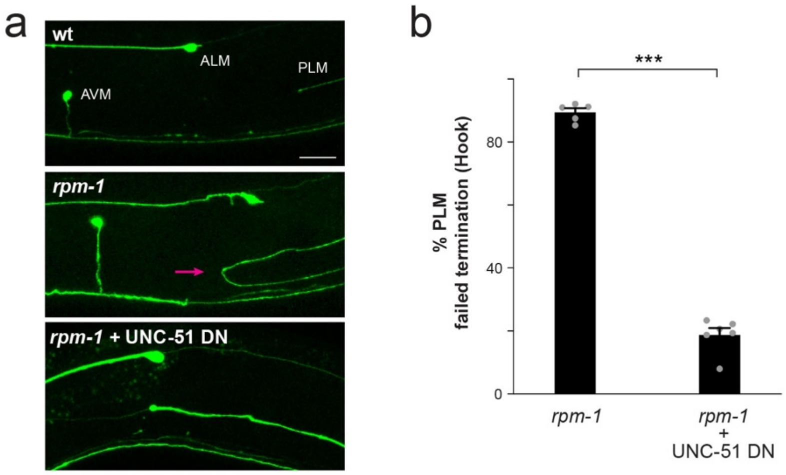

Supplementary Figure 3. UNC-51 DN suppresses failed termination defects in PLM neurons of *rpm-1* mutants. (**a**) Representative images of PLM axons for indicated genotypes. Failed termination defects (magenta arrow) in *rpm-1* mutants are suppressed when UNC-51 DN is expressed in mechanosensory neurons using MosSCI. (**b**) Quantitation of failed termination defects in PLM neurons for indicated genotypes. Means are shown from 5-8 counts (25-35 animals/count) and error bars represent SEM. Significance determined using student's *t*-test with Bonferroni correction. *** $p < 0.001$

Source data are provided as a Source Data file.

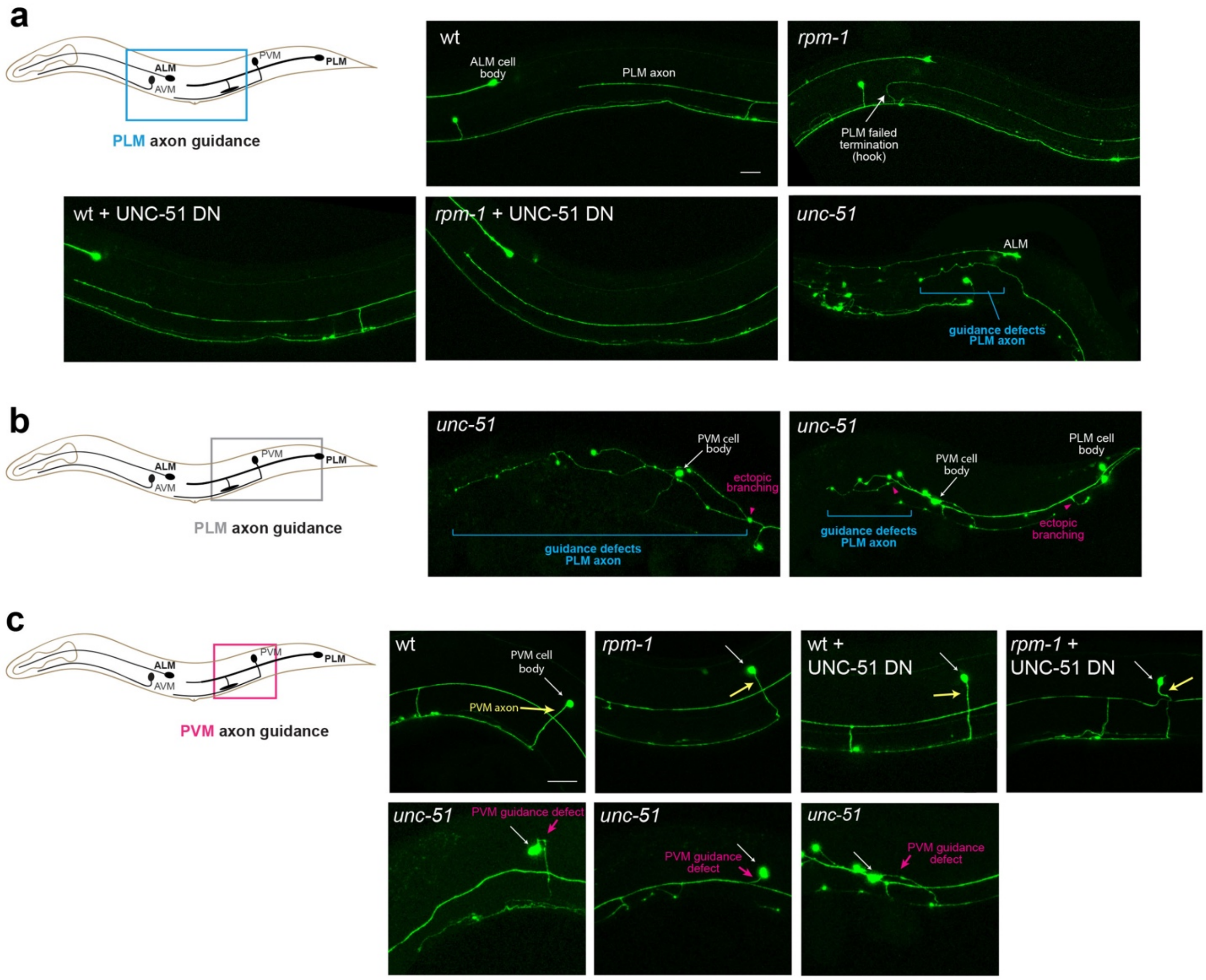

Supplementary Figure 4. UNC-51 DN does not impair axon guidance in PLM or PVM neurons. (**a**) Schematic highlights region imaged (blue box). Shown are representative images of PLM axons for indicated genotypes. Axon guidance defects were not observed in wt animals, *rpm-1* mutants, wt animals expressing UNC-51 DN (MosSCI transgene), or *rpm-1* mutants expressing UNC-51 DN. In contrast, PLM axon guidance was impaired in *unc-51* mutants (blue bracket). (**b**) Schematic highlights more posterior imaging region for PLM axons (grey box). Shown are representative images of further examples of abnormal axon guidance in PLM neurons of *unc-51* mutants. All other genotypes (shown in **a**) showed normal PLM axon guidance in this region. (**c**) Schematic highlights region imaged (magenta box). Shown are representative images of PVM axon guidance for indicated genotypes. Normal PVM axon guidance (in which the axon grows ventrally from the cell body), occurs in wt, *rpm-1*, wt + UNC-51 DN, and *rpm-1* + UNC-51 DN animals. Several examples of abnormal PVM axon guidance are shown for *unc-51* mutants: PVM axon extends dorsally then grows ventrally (bottom, left). PVM axon fails to fully extend ventrally and joins PLM axon tract (bottom, middle). PVM axon extends posteriorly before descending ventrally (bottom, right). Scale bars 20μm.

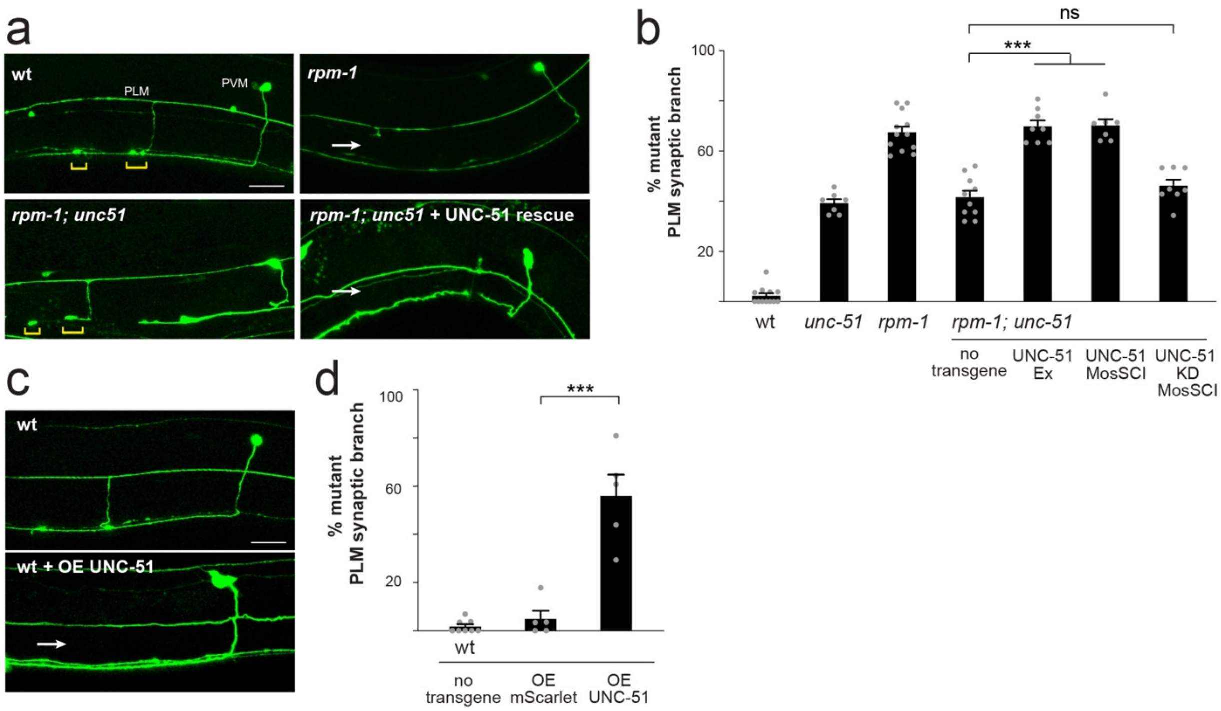

Supplementary Figure 5. UNC-51 kinase activity functions cell autonomously to inhibit synapse maintenance in PLM mechanosensory neurons. (**a**) Representative images of PLM synaptic branch and presynaptic boutons for indicated genotypes. Arrows indicate synapse maintenance defects in which synaptic branch is retracted in *rpm-1* mutants (Borgen *et al*, 2019). Suppression occurs in *rpm-1; unc-51* double mutants (brackets highlight PLM presynaptic boutons). PLM presynaptic boutons are not near PVM axon because of PVM premature termination and/or guidance defects. Suppression in *rpm-1; unc-51* double mutant is rescued by transgenic expression of UNC-51 in mechanosensory neurons. Note in wt animals and *rpm-1; unc-51* mutants presynaptic terminals from both PLMR and PLML are present but only one PLM branch is in focal plane. (**b**) Quantitation of synapse maintenance defects in PLM neurons for indicated genotypes. (**c**) Representative images showing transgenic overexpression of UNC-51 in mechanosensory neurons of wt animals causes loss of PLM synaptic branch (arrow) similar to synapse maintenance defects in *rpm-1* (lf) mutants. (**d**) Quantitation of synapse maintenance defects caused by UNC-51 overexpression. Means are shown from 5-8 counts (25-35 animals/count) and error bars represent SEM. Significance was determined using student's *t*-test with Bonferroni correction. *** $p < 0.001$, ns = not significant. Scale bar 20μm.

Source data are provided as a Source Data file.

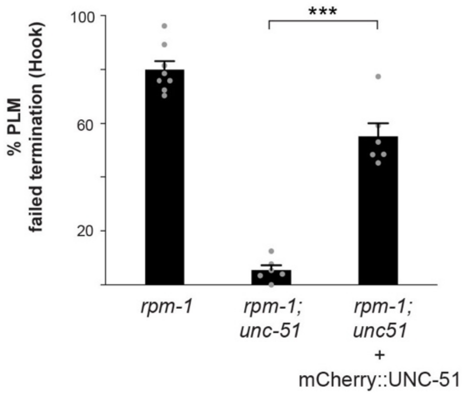

Supplementary Figure 6. N-terminally tagged UNC-51 is functional. Quantitation shows transgenic mCherry::UNC-51 rescues suppression of failed termination defects in *rpm-1; unc-51* mutants indicating this construct is functional. Means are shown from 6-8 counts (25-35 animals/count) and error bars represent SEM. Significance was determined using student's *t*-test with Bonferroni correction. *** $p < 0.001$

Source data are provided as a Source Data file.

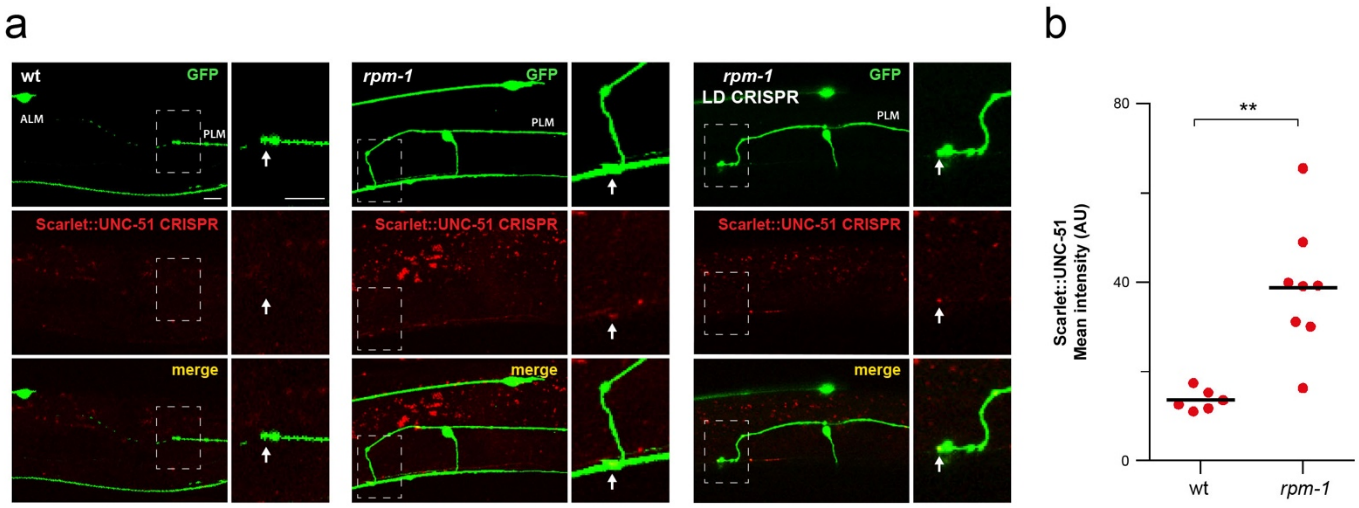

Supplementary Figure 7. RPM-1 ubiquitin ligase activity restricts UNC-51 protein levels at axon termination sites in PLM neurons. (**a**) Representative confocal images showing mScarlet::UNC-51 CRISPR in axons of PLM neurons for indicated genotypes. Transgenic GFP is used to visualize axon morphology. In *rpm-1* (lf) mutants and *rpm-1* LD CRISPR animals, mScarlet::UNC-51 accumulates in puncta at the tips of axons with failed termination (white arrows). (**b**) Quantitation indicates mScarlet::UNC-51 is significantly increased at tips of PLM axons in *rpm-1* (lf) mutants compared to wt animals. Means are shown from 6 or more animals imaged during 2-3 independent experiments for each genotype. Significance was determined using student's *t*-test with Bonferroni correction and error bars represent SEM. ** $p < 0.01$. Scale bars 10μm.

Source data are provided as a Source Data file.

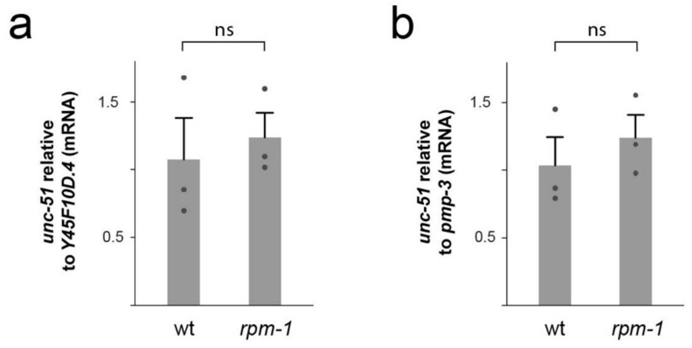

Supplementary Figure 8. *unc-51* mRNA levels are not altered in *rpm-1* mutants. Quantitation of *unc-51* mRNA levels relative to two highly stable mRNAs (**a**) *Y45F10D.4* and (**b**) *pmp-3*. There are no differences in *unc-51* mRNA levels relative to *Y45F10D.4* or *pmp-3* in *rpm-1* mutants compared to wt animals. Results are representative of 3 independent experiments. Means are shown from 3 replicates for each genotype and error bars represent SEM. Significance was determined using Student's *t*-test. ns = not significant

Source data are provided as a Source Data file.

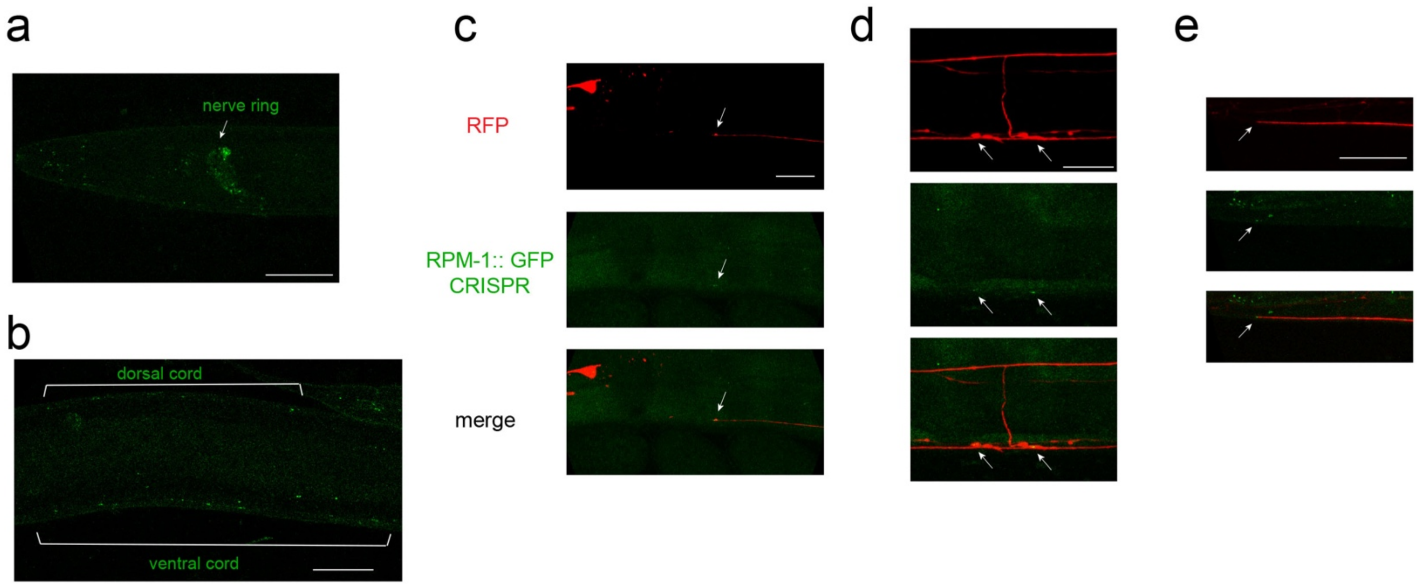

Supplementary Figure 9. RPM-1::GFP CRISPR is present broadly across the nerve system and localizes to axon termination sites and presynaptic terminals. Representative confocal z-stacks showing RPM-1::GFP CRISPR expression in young adults. Shown are (**a**) nerve ring and head neurons, and (**b**) dorsal and ventral motor cords. For (**c-e**) transgenic RFP labels mechanosensory neurons. (**c**) RPM-1::GFP CRISPR localizes to axon termination sites (arrow) of PLM neurons. (**d**) RPM-1::GFP CRISPR localizes to presynaptic boutons (arrows) of PLM neurons. (**e**) RPM-1::GFP CRISPR localizes to axon termination sites of ALM neurons (arrow). Scale bars 20μm

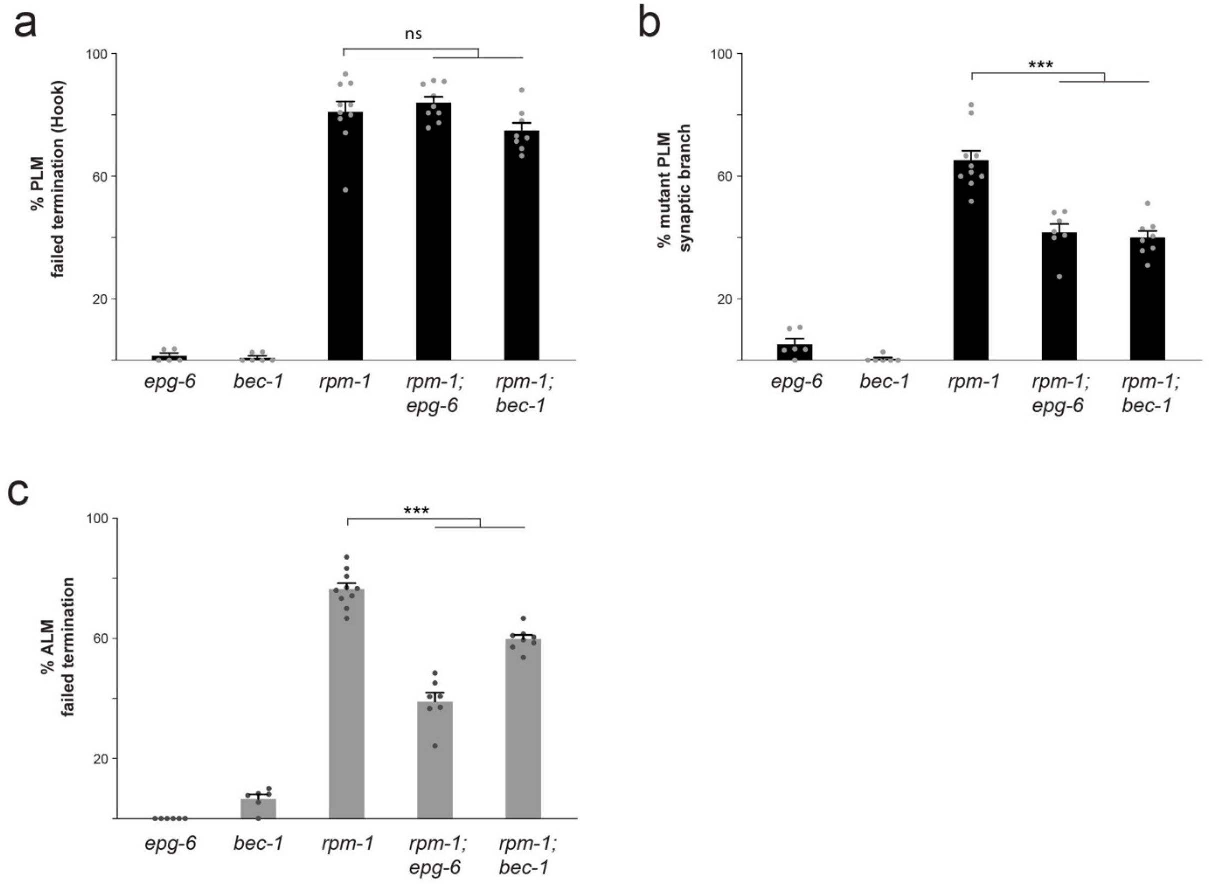

Supplementary Figure 10. *epg-6* and *bec-1* autophagy mutants suppress PLM synapse maintenance defects and ALM axon termination defects caused by *rpm-1* (lf). (**a**) Quantitation of failed termination defects (hook) in PLM neurons for indicated genotypes. PLM termination defects are not suppressed in *rpm-1; epg-6* and *rpm-1; bec-1* double mutants. (**b**) Quantitation of synapse maintenance defects in PLM neurons for indicated genotypes. Synapse maintenance defects are suppressed in *rpm-1; epg-6* and *rpm-1; bec-1* double mutants. (**c**) Quantitation of failed termination defects in ALM neurons for indicated genotypes. ALM termination defects are suppressed in *rpm-1; epg-6* and *rpm-1; bec-1* double mutants. Means are shown from 5-11 counts (25-35 animals/count) for each genotype. Significance determined using student's *t*-test with Bonferroni correction. Source data are provided as a Source Data file.

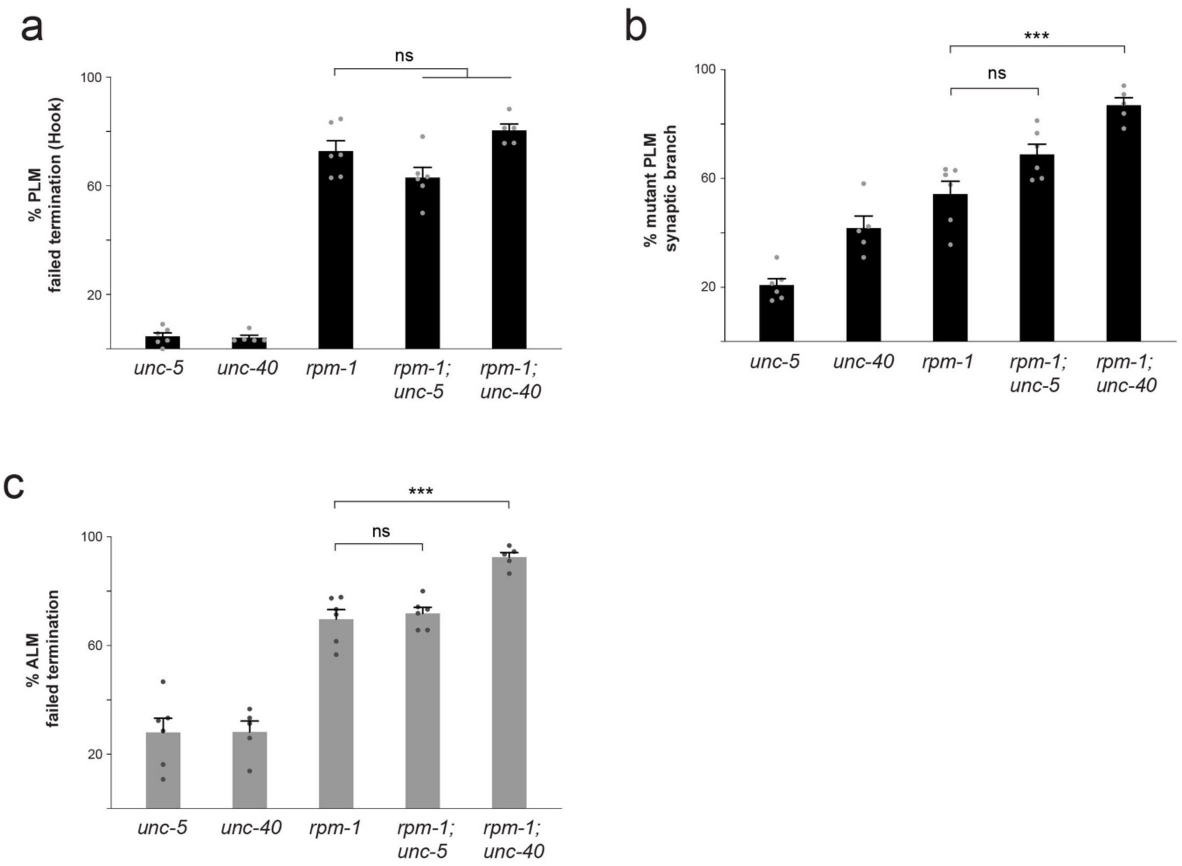

Supplementary Figure 11. Netrin receptor mutants *unc-5* and *unc-40* do not suppress axon termination or synapse maintenance defects in *rpm-1* mutants. Quantitation of (**a**) failed termination defects in PLM neurons, (**b**) synapse maintenance defects in PLM neurons, and (**c**) failed termination defects in ALM neurons for the indicated genotypes. In all cases, no suppression occurs in *rpm-1; unc-5* or *rpm; unc-40* double mutants compared to *rpm-1* single mutants. Increased frequency of defects is observed in *rpm-1; unc-40* double mutants compared to single mutants for (**b**) PLM synapse maintenance defects and (**c**) ALM axon termination defects. Means are shown from 5-8 counts (25-35 animals/count) and error bars represent SEM. Significance was determined using student's *t*-test with Bonferroni correction. *** p< 0.001; ns = not significant.

Source data are provided as a Source Data file.

Protein identified LC-MS/MS	MW (kDa)	Detergent	Total peptide spectra: GS::GFP (control)	Total peptide spectra: GS::RPM-1	Total peptide spectra: GS::RPM-1 LD	Unique peptide spectra: GS::GFP (control)	Unique peptide spectra: GS::RPM-1	Unique peptide spectra: GS::RPM-1 LD
**RPM-1**	418	Tris 0.1% NP-40	0	263	439	0	109	138
		Tris 0.1% NP-40	0	297	525	0	125	154
		Tris 0.3% NP-40	0	206	449	0	97	149
		Tris 0.3% NP-40	0	171	421	0	81	142
		Tris 0.1% CHAPS	0	112	398	0	61	131
		Tris 0.1% CHAPS	0	101	355	0	56	125
		Hepes 0.1% NP-40	0	115	287	0	62	119
**RAE-1**	41	Tris 0.1% NP-40	0	22	46	0	7	16
		Tris 0.1% NP-40	0	28	60	0	14	18
		Tris 0.3% NP-40	0	28	47	0	12	15
		Tris 0.3% NP-40	0	27	54	0	10	18
		Tris 0.1% CHAPS	0	9	43	0	4	15
		Tris 0.1% CHAPS	0	12	35	0	6	15
		Hepes 0.1% NP-40	0	4	22	0	3	10
**GLO-4**	153	Tris 0.1% NP-40	0	35	49	0	18	23
		Tris 0.1% NP-40	0	32	59	0	20	30
		Tris 0.3% NP-40	0	23	40	0	13	20
		Tris 0.3% NP-40	0	26	52	0	14	26
		Tris 0.1% CHAPS	0	0	31	0	0	19
		Tris 0.1% CHAPS	0	0	28	0	0	14
		Hepes 0.1% NP-40	0	0	19	0	0	11
**PPM-2**	39	Tris 0.1% NP-40	0	4	4	0	3	2
		Tris 0.1% NP-40	0	4	4	0	2	2
		Tris 0.3% NP-40	0	5	7	0	3	4
		Tris 0.3% NP-40	0	2	3	0	2	2
		Tris 0.1% CHAPS	0	0	5	0	0	3
		Tris 0.1% CHAPS	0	0	0	0	0	0
		Hepes 0.1% NP-40	0	0	0	0	0	0
**FSN-1**	37	Tris 0.1% NP-40	0	10	44	0	5	7
		Tris 0.1% NP-40	0	11	20	0	7	6
		Tris 0.3% NP-40	0	14	21	0	7	7
		Tris 0.3% NP-40	0	9	18	0	4	6
		Tris 0.1% CHAPS	0	0	17	0	0	7
		Tris 0.1% CHAPS	0	2	13	0	2	6
		Hepes 0.1% NP-40	0	0	14	0	0	7
**SKR-1**	20	Tris 0.1% NP-40	0	0	5	0	0	2
		Tris 0.1% NP-40	0	0	10	0	0	6
		Tris 0.3% NP-40	0	4	7	0	2	4
		Tris 0.3% NP-40	0	0	5	0	0	3
		Tris 0.1% CHAPS	0	2	0	0	2	0
		Tris 0.1% CHAPS	0	0	3	0	0	2
		Hepes 0.1% NP-40	0	0	0	0	0	0
**UNC-51**	95	Tris 0.1% NP-40	0	0	22	0	0	11
		Tris 0.1% NP-40	0	4	24	0	2	9
		Tris 0.3% NP-40	0	4	20	0	3	12
		Tris 0.3% NP-40	0	3	22	0	2	9
		Tris 0.1% CHAPS	0	0	11	0	0	7
		Tris 0.1% CHAPS	0	0	14	0	0	6
		Hepes 0.1% NP-40	0	0	10	0	0	5
**UNC-14**	74	Tris 0.1% NP-40	0	0	34	0	0	16
		Tris 0.1% NP-40	0	4	36	0	3	18
		Tris 0.3% NP-40	0	2	29	0	2	12
		Tris 0.3% NP-40	0	2	28	0	2	12
		Tris 0.1% CHAPS	0	0	23	0	0	9
		Tris 0.1% CHAPS	0	0	13	0	0	6
		Hepes 0.1% NP-40	0	0	16	0	0	6

Supplementary Table 1. Results from 7 independent proteomic experiments with GS::RPM-1, GS::RPM-1 LD and GS::GFP (negative control). Shown are detergent conditions used for extraction and number of peptides identified for a given protein in individual proteomic experiments.

Supplementary Table 2: Transgenic and CRISPR Strains

Figure	Strain Name	Genotype
1A, 1C, S1	XMN829	*muIs32* II; *rpm-1(ju44) bggIs9* [P$_{rpm-1}$ GS::RPM-1; P$_{myo-2}$ mCherry, *pha-1(+)*] V
1A, 1C, S1	XMN830	*muIs32 bggIs19* [P$_{rpm-1}$ GS::RPM-1 LD; P$_{myo-2}$::mCherry, *pha-1(+)*] II; *rpm-1(ju44)* V
1C, S1	XMN831	*muIs32* II; *rpm-1(ju44)* V; *bggIs23* [P$_{rpm-1}$ GS::GFP; P$_{myo-2}$ mCherry, *pha-1(+)*]
1F	XMN841	*rpm-1(bgg6 [rpm-1::GFP CRISPR])* V
1F	XMN948	*rpm-1(bgg6 bgg40 [rpm-1 LD::GFP CRISPR])* V.
1F	XMN941	*unc-51(bgg23 [3xFLAG::unc-51 CRISPR])* V.
1F	XMN983	*rpm-1(bgg6 [rpm-1::GFP CRISPR]) unc-51(bgg23 [3xFLAG::unc-51 CRISPR)* V.
1F	XMN986	*rpm-1(bgg6 bgg40 [rpm-1 LD::GFP CRISPR]) unc-51(bgg23 [3xFLAG::unc-51 CRISPR)* V.
3G, S4	XMN1084	*bggSi4* [P*mec-7* UNC-51 DN (K39I); P*rps-27* NeoR] *muIs32* II;
3D, 3E, 3G S3, S4	XMN970	*bggSi4* [P*mec-7* UNC-51 DN (K39I); P*rps-27* NeoR] *muIs32* II; *rpm-1(ju44)* V
4B, 4C, S5A, S5B	XMN1001	*bggSi20* [P$_{mec-7}$ UNC-51; P$_{rps-27}$ NeoR] *muIs32* II; rpm-1*(ju44) unc-51(e369)* V
4B, 4C, S5B	XMN971	*bggSi4* [P$_{mec-7}$ UNC-51 KD (K39I); P$_{rps-27}$ NeoR] *muIs32* II; *rpm-1(ju44) unc-51(e369)* V
5A	XMN895	*unc-51(bgg18 [mScarlet::unc-51 CRISPR])* V
5B, 5C, 5E, 5F, 5G, S7, S8	XMN920	*muIs32* II; *unc-51(bgg18 [mScarlet::unc-51 CRISPR]*) V
5B, 5C, 5E, 5F, 5G, S7, S8	XMN921	*muIs32* II; *rpm-1(ju44) unc-51(bgg18 [mScarlet::unc-51 CRISPR])* V
5B, 5C, 5E, 5F, S7	XMN965	*muIs32* II; *rpm-1(bgg6 bgg39 [rpm-1 LD::GFP CRISPR]) unc-51(bgg18 [mScarlet::unc-51 CRISPR])* V
6A	XMN845	*jsIs973* III; *rpm-1(ju44)* V; *glo-1(zu391)* X; *bggIs34* [P$_{mec-3}$ RPM-1::GFP; P$_{myo-2}$ mCherry]
6B	XMN874	*jsIs973* III; *glo-1(zu391)* X; *bggIs44* [P$_{mec-3}$ RPM-1 LD::GFP; P$_{myo-2}$ mCherry]

Continued on next page

Supplementary Table 2: Continued

Figure	Strain Name	Genotype
7B, 7C, 7D, 7E, 7F	XMN968	*bggSi9* [P$_{mec-7}$ ATG-9; P$_{rps-27}$ NeoR] *muIs32* II; *atg-9(bp564) rpm-1(ju44)* V
7B, 7C, 7D, 7E, 7F	XMN969	*bggSi10* [P$_{mec-7}$ ATG-9; P$_{rps-27}$ NeoR] *muIs32* II; *atg-9(bp564) rpm-1(ju44)* V
7G, 7H	XMN972:	*bggSi14* [P$_{mec-7}$ mCherry::GFP::LGG-1*;* P$_{rps-27}$::NeoR] II
7G, 7H	XMN973	*bggSi14* [P$_{mec-7}$ mCherry::GFP::LGG-1*;* P$_{rps-27}$::NeoR] II; *rpm-1(ju44)* V
8A, 8B	DLM12	*uwaSi6* [P$_{rab-3}$ oxCerulean::oxVenus::LGG-1] II
8A, 8B	XMN998	*uwaSi6* [P$_{rab-3}$ oxCerulean::oxVenus::LGG-1] II; *rpm-1* (*ju44*) V
8E, 8F	XMN938	*jsIs973* III; *atg-9(ola274 [atg-9::GFP CRISPR])* V
8E, 8F	XMN939	*jsIs973* III; *atg-9(ola274 [atg-9::GFP CRISPR]) rpm-1(ju44)* V
8C, 8D	XMN961	*bggSi16* [P$_{rab-3}$ mCherry::GFP::LGG-1*;* P$_{rps-27}$ NeoR] II
8C, 8D	XMN999	*bggSi16* [P$_{rab-3}$ mCherry::GFP::LGG-1*;* P$_{rps-27}$ NeoR] II; *rpm-1* (*ju44*) V
S9	XMN1000	*jsIs973* III*; rpm-1(bgg6 [rpm-1::GFP CRISPR])* V*; glo-1(zu391)* X

Supplementary Table 3: Injection Conditions

Figure	Transgene/ CRISPR allele	Injected Strain	Injection Mix
4A-C, S5A, S5B	P_{mec-7} UNC-51 Ex rescue (multiple lines)	*rpm-1*(*ju44*) *unc-51*(*e369*); *muIs32*	10 ng/µl pBG-GY858 (P_{mec-7} UNC-51)<br>1 ng/µl pCFJ90 (P_{myo-2} mCherry)<br>89 ng/µl pBlueScript
4D, 4E, S5C, S5D	P_{mec-7} UNC-51 Ex overexpression (multiple lines)	*muIs32*	10 ng/µl pBG-GY858 (P_{mec-7} UNC-51)<br>1 ng/µl pCFJ90 (P_{myo-2} mCherry)<br>89 ng/µl pBlueScript
4E, S5D	P_{mec-7} mScarlet Ex (multiple lines)	*muIs32*	10 ng/µl pBG-GY861 (P_{mec-7} mScarlet)<br>1 ng/µl pCFJ90 (P_{myo-2} mCherry)<br>89 ng/µl pBlueScript
S6	P_{mec-7} mCherry::UNC-51 Ex (multiple lines)	*rpm-1*(*ju44*) *unc-51*(*e369*); *muIs32*	10 ng/µl pBG-GY860 (P_{mec-7} mCherry::UNC-51)<br>1 ng/µl pCFJ90 (P_{myo-2} mCherry)<br>89 ng/µl pBlueScript
6C, 6D	*bggEx142* [P_{mec-7} mScarlet:: UNC-51 KD(K39R) + P_{mec-7} FSN-1]	*glo-1(zu391); bggIs44*	10 ng/µl pBG-GY879 (P_{mec-7} mScarlet::UNC-51 K39R)<br>80 ng/µl pBG-GY877 (P_{mec-7} FSN-1)<br>1 ng/µl pBG-264 (P_{rps-27} NeoR)<br>49 ng/µl pBlueScript
1A, 1C, S1	*bggIs9* [P_{rpm-1} GS::RPM-1]	*rpm-1(ju44); pha-1(e2123); muIs32*	50 ng/µl pBG186 (P_{rpm-1} GS::RPM-1)<br>1 ng/µl pCFJ90 (P_{myo-2} mCherry)<br>25 ng/µl pBX (*pha-1(+)*)<br>25 ng/µl pBlueScript
1A, 1C, S1	*bggIs19* [P_{rpm-1} GS::RPM-1 LD]	*rpm-1(ju44); pha-1(e2123); muIs32*	50 ng/µl pBG-255 (P_{rpm-1} GS::RPM-1 LD)<br>1 ng/µl pCFJ90 (P_{myo-2} mCherry)<br>25 ng/µl pBX (*pha-1(+)*)<br>25 ng/µl pBlueScript
1C, S1	*bggIs23* [P_{rpm-1} GS::GFP]	*rpm-1(ju44); pha-1(e2123); muIs32*	18.5 ng/µl pBG-254 (P_{rpm-1} GS::GFP)<br>1 ng/µl pCFJ90 (P_{myo-2} mCherry)<br>25 ng/µl pBX (*pha-1(+)*)<br>25 ng/µl pBlueScript
6A	*bggIs34* [P_{mec-3} RPM-1 GFP]	*rpm-1(ju44); glo-1(zu391); jsIs973*	20 ng/µl pBG-46 (P_{mec-3} RPM-1 GFP)<br>1 ng/µl pCFJ90 (P_{myo-2} mCherry)<br>79 ng/µl pBlueScript
6B, 6C, 6D	*bggIs44* [P_{mec-3} RPM-1 LD::GFP]	*rpm-1(ju44); glo-1(zu391); jsIs973*	60 ng/µl pBG-348 (P_{mec-3} RPM-1 LD::GFP)<br>1 ng/µl pCFJ90 (P_{myo-2} mCherry)<br>39 ng/µl pBlueScript
3D, 3E, 3G, 4B, 4C, S3, S4	*bggSi4* [P_{mec-7} UNC-51 DN/KD (K39I)]	*ttTi5605*	22.5 ng/µl pBG-GY911 (ttTi5605 MosSci [P_{mec-7} UNC-51 K39I; P_{rps-27} NeoR])]<br>50 ng/µl pCFJ601 (P_{eft-3} Mos1 transposase)<br>10 ng/µl pMA122 ($P_{hsp-16.41}$ PEEL-1)<br>10 ng/µl pGH8 (P_{rab-3} mCherry)<br>5 ng/µl pCFJ104 (P_{myo-3} mCherry)<br>2.5 ng/µl pCFJ90 (P_{myo-2} mCherry)

Continued on next page

Supplementary Table 3: Continued

Figure	Transgene/ CRISPR Allele	Injected Strain	Injection Mix
7B-F	*bggSi9; bggSi10* [P_{mec-7} ATG-9]	*ttTi5605*	22.5 ng/µl pBG-GY933 (*ttTi5605* MosSCI [P_{mec-7} ATG-9; P_{rps-27} NeoR])
			50 ng/µl pCFJ601 (P_{eft-3} Mos1 transposase)
			10 ng/µl pMA122 ($P_{hsp-16.41}$ PEEL-1)
			10 ng/µl pGH8 (P_{rab-3} mCherry)
			5 ng/µl pCFJ104 (P_{myo-3} mCherry)
			2.5 ng/µl pCFJ90 (P_{myo-2} mCherry)
7G, 7H	*bggSi14* [P_{mec-7} mCherry:: GFP::LGG-1]	*ttTi5605*	22.5 ng/µl pBG-GY924 (*ttTi5605* MosSCI [P_{mec-7} mCherry::GFP::LGG-1; P_{rps-27} NeoR])
			50 ng/µl pCFJ601 (P_{eft-3} Mos1 transposase)
			10 ng/µl pMA122 ($P_{hsp-16.41}$ PEEL-1)
			10 ng/µl pGH8 (P_{rab-3} mCherry)
			5 ng/µl pCFJ104 (P_{myo-3} mCherry)
			2.5 ng/µl pCFJ90 (P_{myo-2} mCherry)
8C, 8D	*bggSi16* [P_{rab-3} mCherry:: GFP::LGG-1]	*ttTi5605*	22.5 ng/µl pBG-GY926 (*ttTi5605* MosSCI [P_{rab-3} mCherry::GFP::LGG-1; P_{rps-27} NeoR])
			50 ng/µl pCFJ601 (P_{eft-3} Mos1 transposase)
			10 ng/µl pMA122 ($P_{hsp-16.41}$ PEEL-1)
			10 ng/µl pGH8 (P_{rab-3} mCherry)
			5 ng/µl pCFJ104 (P_{myo-3} mCherry)
			2.5 ng/µl pCFJ90 (P_{myo-2} mCherry)
4B, 4C, S5B	*bggSi20* [P_{mec-7} UNC-51]	*ttTi5605*	20 ng/µl pBG-GY910 (*ttTi5605* MosSCI [P_{mec-7} UNC-51; P_{rps-27} NeoR]
			50 ng/µl pCFJ601 (P_{eft-3} Mos1 transposase)
			10 ng/µl pMA122 ($P_{hsp-16.41}$ PEEL-1)
			10 ng/µl pGH8 (P_{rab-3} mCherry)
			5 ng/µl pCFJ104 (P_{myo-3} mCherry)
			2.5 ng/µl pCFJ90 (P_{myo-2} mCherry)
1F, S9	*bgg6* CRISPR [RPM-1::GFP]	*pha-1(e2123)*	5 µL tracrRNA (4 ug/µl)
			0.4 µL *pha-1* crRNA (8 ug/µl)
			0.55 µL *pha-1* ssODN (500 ng/µl)
			1.0 µL RPM-1::GFP crRNA (8 ug/µl)
			6.8 µL RPM-1::GFP PCR repair template (500 ng/µl)
			0.5 µL KCl (1M)
			0.75 µL Hepes pH7.4 (200mM)
			5 µL Cas9 (10 ug/µl)

Continued on next page

Supplementary Table 3: Continued

Figure	Transgene/ CRISPR Allele	Injected Strain	Injection Mix
1F, 5B, 5C, 5E, 5F, S7	*bgg39; bgg40* CRISPR [RPM-1 LD::GFP]	*rpm-1(bgg6)*	5 µL tracrRNA (4 ug/µl)
			0.4 µL *dpy-10* crRNA (8 ug/µl)
			0.55 µL *dpy-10* ssODN (500 ng/µl)
			1.0 µL RPM-1 LD crRNA (8 ug/µl)
			2.2 µL RPM-1 LD ssODN repair template (1 ug/µl)
			0.5 µL KCl (1M)
			0.75 µL Hepes pH7.4 (200mM)
			4.6µL dH_2O
			5 µL Cas9 (10 ug/µl)
5A-C, 5E-G, S7, S8	*bgg18* CRISPR [mScarlet::UNC-51]	*pha-1(e2123)*	5 µL tracrRNA (4 ug/µl)
			0.4 µL *pha-1* crRNA (8 ug/µl)
			0.55 µL *pha-1* ssODN repair template (500 ng/µl)
			1.0 µL *unc-51* crRNA (8 ug/µl)
			6.8 µL mScarlet::UNC-51 PCR repair template (500 ng/µl)
			0.5 µL KCl (1M)
			0.75 µL Hepes pH7.4 (200mM)
			5 µL Cas9 (10 ug/µl)
1F	*bgg23* CRISPR [3xFLAG::UNC-51]	*pha-1(e2123)*	5 µL tracrRNA (4 ug/µl)
			0.4 µL *pha-1* crRNA (8 ug/µl)
			0.55 µL *pha-1* repair ssODN (500 ng/µl)
			1.0 µL *unc-51* crRNA (8 ug/µl)
			2.2 µL 3xFLAG::UNC-51 ssODN (1 ug/µl)
			0.5 µL KCl (1M)
			0.75 µL Hepes pH7.4 (200mM)
			4.6µL dH_2O
			5 µL Cas9 (10 ug/µl)

Supplementary Table 4: CRISPR Targeting Sequences and Repair Templates

Gene	crRNA	crRNA target sequence	Repair Template	Repair Template Sequence
rpm-1	RPM-1::GFP	GCAGGAGCATTAGTACACGA	RPM-1::GFP PCR	5'ATCTGGCAGTCCTGTGAAATT**CCG**TCATGCACAAATGCACCTGCAGCGCTTAAGTTGGGAATTCAAGTACCGGTAGAAAAA ---GFP---- AGCCCACAAGCTTTCACGCGTCCTGGTACCGCTGCAGCTCTCCAACCAATAGTGAAAATTTGGAGAGAAATATG
rpm-1	RPM-1 LD	CCATGTATTCGCCTCGGATG	RPM-1 LD ssODN	5'AAAGACTTGGAGCAGCACCATGTATTAGGCTGGGCGC**AGG**TGCAATGTTCGCATTCCATTGTGTTCGAATGATTTTGGAAAGAAGATG
unc-51	UNC-51	AGCTGAATGGAGCAGTTTGA	mScarlet::UNC-51 PCR	5'CCCGCCAAAACCTATATAGTCACACAATAATCACCCAACCCAGCTGA ----mScarlet---- CCTAGGGTCGATCAAACAAGTTTGTACAAAAAAGCAGGCTCCGAATTCGCCCTTGCCGGCATGGAACAGTTCGA**CGG**CTTCGAGTACAGCAAACGGGACCTTTTAGGTCATGGAGCATTTGCAATTG
unc-51	UNC-51	AGCTGAATGGAGCAGTTTGA	3xFLAG::UNC-51 ssODN	5'CCTATATAGTCACACAATAATCACCCAACCCAGCTGA -- 3xFLAG -- GGAGGAGGAGGATCCGGAGGAGGAGGATCCGGAGGAGGAGGATCCATGGAACAGTTCGA**CGG**CTTCGAGTACGCAAACGGGACCTTTTAGGCTTTTAGG
pha-1	*pha-1* repair	ATGAATAACTTGATGAACAT	*pha-1* ssODN	CAAAATACGAATCGAAGACTCAAAAAGAGTATGCTGTATGATTA**CAG**ATGTTCATCAAGTTATTCATAAATCATTGATAG
dpy-10	*dpy-10* repair	GCTACCATAGGCACCACGAG	*dpy-10* ssODN	5'CACTTGAACTTCAATACGGCAAGATGAGAATGACTGGAAACCGTA**CCG**CATGCGGTGCCTATGGTAGCGGAGCTTCACATGGCTTCAGACCAACAGCCTAT

lowercase (non-coding sequence); uppercase (coding sequence); underline (crRNA targeting sequence) Bold (Pam sequence), Red (silent mutations in repair to prevent Cas9 re-cutting), Blue (Insertion or gene edit), Orange (linker)

Supplementary Table 5: qPCR oligonucleotides

Oligo	
unc-51 5’	5’ CTACTCAAATGCACGGAGACC
unc-51 3’	5’ TCAAATCACGATGTACGATGC
tba-1 5’	5’ TCAACACTGCCATCGCCGCC
tba-1 3’	5’ TCCAAGCGAGACCAGGCTTCAG
pmp-3 5’	5’ TGGCCGGATGATGGTGTCGC
pmp-3 3’	5’ ACGAACAATGCCAAAGGCCAGC
Y45F10D.4 5’	5’ CGGTTGCCAGGGAAGATGAGGC
Y45F10D.4 3’	5’ TGGCCGGATGATGGTGTCGC
