# Supplementary material for: Autophagy is inhibited by ubiquitin ligase activity in the nervous system
Source: Nat Commun. 2019 Nov 1;10:5017. doi: 10.1038/s41467-019-12804-3 (PMC6825199; doi:10.1038/s41467-019-12804-3)
Supplement: Supplementary file 2 — Peer Review [file 41467_2019_12804_MOESM2_ESM.pdf]

Reviewers' comments:

Reviewer #1 (Remarks to the Author):

Crawley et al. address the question of “how is autophagy regulated in neurons”? They perform a proteomics screen combined with genetic analysis in C. elegans to identify that the RPM-1 ubiquitin ligase negatively regulates the autophagy initiator UNC-51/ULK kinase to control axonal and synaptic development. The model generated is that RPM-1 targets UNC-51 for proteasomal degradation specifically within axon terminals to locally downregulate autophagy and allow for proper termination of axons in the developing nervous system. These findings are very intriguing and will provide significant insights for the neuronal autophagy field. Further, the authors uncover a new mechanism that defines crosstalk between the UPS and autophagy to coordinate cellular degradation in neurons. The data are of high quality and presented well. The following points, however, need to be addressed before the paper can be acceptable for publication:

Major points:
1. Can the authors clarify the temporal sequence of events in their model? The model indicates that low autophagy supports termination and high autophagy is correlated with axon overgrowth. Therefore, in WT worms, is RPM-1 enriched at the axon terminus only at the time of termination? Have the authors checked earlier time points in development to examine whether RPM-1 levels are low in growth cones during the elongation phase, upstream of termination, to allow for higher autophagy to promote axon growth? In other words, are autophagy levels in the growth cone suddenly decreased upon arrival at the target? Perhaps higher levels of autophagy are needed during axon extension, but then levels need to decrease for termination to occur. Discriminating between these points would illuminate different roles for autophagy in promoting axon growth but inhibiting termination. How do these results reconcile with previous literature that loss of autophagy proteins decreases axon outgrowth in the CNS (Dragich et al., eLife 2016, and others)?

2. The failed termination phenotype with the rpm-1 mutant is not completely suppressed by mutations in atg-9 (or epg-6). These results indicate that either (1) alternative mechanisms are also at play, independent of autophagy, or (2) the atg-9 mutant does not completely block autophagy. Can the authors reconcile these points? Recent literature in mammalian cells indicates that loss of atg-9 from the TGN does not block basal autophagy, but only stress-induced autophagy (De Pace et al. PLOS Genetics 2018). Further, Atg9 may have roles in axon outgrowth independent of autophagy (Yamaguchi et al. Autophagy 2017). Thus, mutants of Atg9 may confound interpretations. Can the authors use atg5 or atg7 mutants to block autophagy or are there lethality concerns?

3. To clarify the identity of the structures in Fig. 7G, higher resolution images are needed.

4. There is no direct evidence for direct proteasomal degradation in Fig. 5. Can the authors use MG132 in the SH-SY5Y model? Alternatively, can they exclude altered localization of UNC-51/Ulk1 in the absence of RPM-1 in the worm model?

Minor points:
1. Figure 7: mCherry signal in the axon terminus is barely visible in WT images. Can the authors correct this?
2. Figure 1F: Authors should make a table listing the identity of proteins isolated in different detergent conditions.
3. Figure 7e: typo in y-axis.
4. Figure 9: Model on the right side is unclear. Are levels of autolysosomes in the soma the same in WT vs rpm-1 mutant (as it is depicted in the model), even though more autophagosomes are generated? Is this model based on a quantitation of autolysosome density in the soma?

Reviewer #2 (Remarks to the Author):

The manuscript entitled “Autophagy is Inhibited by Ubiquitin Ligase Activity in the Nervous System” by Crawley et al. highlights a previously undescribed relationship between the E3 ubiquitin ligase RPM-1 and the serine-threonine kinase UNC51 that affects axon growth. Although the data are intriguing, a few critical controls are missing and the data regarding the role of autophagy in the phenotype is preliminary. Major concerns are listed below:

1. Figure 1 nicely shows the trapping of UNC-51 by ligase-defective RPM-1 using an unbiased proteomics approach. The authors should provide biochemical evidence that UNC-51 is indeed a substrate whose turnover is regulated in an RPM-1 dependent manner. For example, based on the model, one would predict that proteasome-mediated inhibition should result in the accumulation of ubiquitinated forms of FLAG::UNC-51, and that rpm1 mutant worms would show elevated steady state levels of FLAG::UNC-51 with loss of the proteasome inhibitor effect. Biochemical data would help to strengthen the conclusions drawn from the imaging based data presented in figure a-f. [Comment: the authors may consider presenting the data in Figure 5 and 6 after Figure 1 to better set up the rationale for the genetic experiments and improve the flow of the manuscript].
2. The data in Figure 5h shows an increase in steady state levels of ULK1 upon shRNA-mediated silencing of PAM, but as the difference is eliminated after CHX treatment the results suggest that the difference stems from increased translation, not a defect in turnover. This experiment needs to be performed in a more rigorous manner – ideally with calculation of ULK1 half-life in NT control and shPAM cells. As presented, the data do not support the conclusions.
3. The results of the genetic experiments presented in Figures 2-5 are intriguing, but in most cases the phenotype of rpm1 mutants reverts to that of the unc-51 mutant. Can the authors exclude the possibility that problems with axon growth and guidance in the unc-51 mutants are upstream of the defects caused by loss of rpm-1 and prevent the manifestation of the defects caused by loss of rpm-1 (and are unrelated to a failure to degrade UNC-51)? Perhaps crossing unc-51 mutants with another genetic mutant that causes failed termination or hooking (similar to rpm-1 mutants) to see if those phenotypes persist in double mutants would help to resolve the issue.
4. What happens to the premature termination of ALM/PLM neurons, overextension of PLM neurons and PLM branching defects observed in rpm-1, unc-51 double mutants upon overexpression of WT UNC-51 or KD UNC-51? This is an important control.
5. The authors demonstrate that overexpression of UNC-51 causes failed termination in PLM axons and branching defects – does it cause any overextension or premature termination? What is the effect of UNC-51 overexpression on ALM axons?
6. The authors claim that excessive autophagy in rpm-1 mutants accounts for the defects in axon termination. This is based on the reversal of the phenotype in atg9 mutants and the observation of increased LGG-GFP fluorescence in neurons. I have several concerns regarding the conclusion. First, a recent publication demonstrates that disrupting Atg9a in mice causes axon guidance defects that are not observed in other canonical autophagy knockouts (i.e. atg7-KO, atg16l1 KO), but are similar to those caused by ULK1/2-deficiency (Yamagichi et al. Autophagy 14:764-777, 2018). Therefore, the observation that disrupting atg9 but not epg6 reverses the phenotype in rpm-1 could suggest that the phenotype is unrelated to autophagy. Moreover, just because autophagy (or at least LGG-GFP) appears to be increased in rpm-1 mutant worms does not mean that this increase is responsible for the phenotype. Thus, the conclusion that the axon termination defects in rpm-1 mutants are caused by excessive UNC-51 dependent autophagy is premature. Additional studies would have to be performed (e.g. examining the effect of disrupting other autophagy genes on the rpm-1 mutant phenotype) to convince readers of the connection (or lack thereof) with autophagy. If other autophagy genes do not rescue, then the authors should tone down their conclusions regarding the regulation of autophagy by rpm-1 and instead focus on its regulation of UNC51 function (whether autophagy-related or unrelated), using the results of atg9 and other autophagy mutants to help delineate the pathway.

Reviewer #3 (Remarks to the Author):

The manuscript by Crawley et al reports the identification of C. elegans ATG kinase UNC-51 as an in vivo substrate of the RPM-1 ubiquitin E3 ligase. Previous studies from the same lab have characterized a number of proteins that bind RPM-1, from an early proteomics analysis of wild type RPM-1. Here, the authors designed a ligase-dead RPM-1 to enrich candidate ubiquitination substrates. Together, with the use of a protein-G::streptavidin binding peptide purification tag, they obtained many more RPM-1 binding proteins. UNC-51 kinase is a top hit among those showing enrichment for ligase-dead RPM-1. They addressed the in vivo relevance of UNC-51 in neuronal development regulated by RPM-1, employing a set of well-established genetic assays on the axon termination and synapse pattern of the mechanosensory neurons. Most of the data support their conclusion that RPM-1 acts upstream of UNC-51 and inhibits UNC-51 abundance in axon ends. They also show that in human neuroblastoma cells ULK1 protein levels are regulated by the human protein PAM, the homolog of RPM-1. Given the role of ATG kinase in autophagy, they addressed whether UNC-51's role involved autophagy process. Through quite extensive genetic interaction and cellular marker analyses, they conclude that RPM-1 restricts autophagosome formation at axon termination site.

The strength of the paper includes the improved proteomics analyses that reveal new binding partners of RPM-1, the functional dissection of UNC-51 in RPM-1 mediated neuronal development and maintenance, and the implication of autophagy being downstream of UNC-51 and RPM-1 interaction. The amount of data is overwhelming, and the technical range is impressive. An obvious weakness is the lack of direct evidence that UNC-51 is ubiquitinated by RPM-1. The effects of RPM-1 on autophagosomes are subtle. Additionally, there are some over-stated conclusions throughout the manuscript, and some statements need further clarification.

Specific comments:

-Fig 1d , Table 1 and the text on pg 4-5: the authors state 'More importantly, our LC-MS/MS analysis identified a striking number of novel RPM-1 interactors, and putative ubiquitination targets that were enriched in RPM-1 LD samples compared to wt RPM-1 (Fig. 1d).'

However, the complete list of proteins in Fig 1d. is not included in the manuscript. This data omission may be an oversight, and certainly reduces the level of enthusiasm for their claimed success of their proteomics.

-pg 6-7: the authors drew a strong conclusion'Thus, in rpm-1; unc-51 double mutants the unc-51 (lf) phenotype fully dominates, which indicates RPM-1 functions as an upstream inhibitor of UNC-51.'.

Given the known role of unc-51 in axon guidance, presumably early development, a caveat to this interpretation is that the two genes may function in different phases of axon development and maintenance. This might be a difficult issue to resolve solely based on snap-shot observations on axon morphology.

- pg 8: 'In unc-51 mutants, we observed synaptic branch loss (Fig. 3b, c).'.

It is not clear whether their data actually show the synaptic branch was formed first, then lost, or simply never formed.

At the end of this section, 'While there is no example of rpm-1 suppression affecting synaptic branch defects and not influencing synapse formation, our results do not rule out this possibility. More importantly, our results indicate that UNC-51 and UNC-14 have different functional relationships with RPM-1 during synapse formation.'.
It is not clear why the last statement is 'more important' for what reason.

-pg 13: ‘Unfortunately due to overexpression phenotypes (Fig. 4d), we could not consistently visualize mScarlet::UNC-51 at normal axons termination sites when it was expressed in mechanosensory neurons. ‘.

This is rather difficult to understand, as the data in Fig 4d show about 30% PLM being abnormal. Can they observe the remaining 70% normal PLM?

-pg 14, figure 7e, f, the data on ATG-9 rescue seems to show partial activity of ATG-9. Is this the case? statistic analysis should be applied to the data for rpm-1; atg-9+ ATG-9 rescue relative to rpm-1.
Also, the authors wrote ‘Finally, suppression of defects caused by rpm-1 (lf) was not as strong in rpm-1; atg-9 or rpm-1; epg-6 double mutants as rpm-1; unc-51 animals. This is most likely because UNC-51 is the primary activator of autophagy, …’.

Can they rule out that this is not due to unc-51’s roles in transport?

- Minor points on the style of writing: it seems that the authors have a tendency to repeat a number of statements or state too soon.
e.g. the top of pg 7 ‘We observed axon guidance defects in PLM neurons of unc-51 mutants, but this did not prevent us from evaluating axon termination.’, which is already stated on pg 6.
Also, the conclusion paragraph on pg 7 ‘Overall, these genetic results support several conclusions. …’ . In this reviewer’ opinion, the three conclusions depend on the data in later Figures.

-generally, the authors should be consistent in writing whether they mean ‘unc-51’ or ‘rpm-1’ to be same as loss of function. e.g. pg 9. ‘Our studies demonstrate that unc-51 suppresses rpm-1 (Fig. 2, 3)’. Presumably, they mean to say loss of function in unc-51?

-Labels, i.e. words and numbers, on panels in Fig 1e, and many graphs in Fig 2e, f, and like are too small.

We thank all three reviewers for their valuable and helpful comments, which have resulted in a significantly improved manuscript. We appreciate all 3 reviewers noting the significance and quality of our study. We have addressed all reviewer comments with additional new experiments as well as textual revisions to our paper. Below we detail the most important new experimental additions.

1) We provide new data with cell-based experiments showing that human PAM is sufficient for ubiquitination of ULK, and PAM ubiquitination of ULK is increased when the proteasome is inhibited (Fig 5j).

2) We provide extensive new data showing that an UNC-51 dominant negative construct (UNC-51 DN) expressed as a Mos single copy transgene does not impact axon guidance (Supplemental Fig 4), but strongly suppresses axon termination and synapse maintenance defects caused by *rpm-1* (lf) (Fig 3d, e; S3). New results also indicate this reagent does not impair synapse maintenance on its own on a wt background (Supplemental Fig 4A, C). We also clarify that this is an established UNC-51 dominant negative construct (see responses below, and new text in Results section of paper). These new experimental results, along with findings in our original manuscript that are now presented more clearly, support our conclusion that effects of RPM-1 inhibition of UNC-51 on axon termination and synapse maintenance are molecularly distinct from effects UNC-51 has on axon guidance. Moreover, results that were in our original manuscript showing *unc-14* (which functions with *unc-51* to regulate axon guidance) does not suppress *rpm-1* provides further support for this conclusion. While these findings with *unc-14* were in our original manuscript, their implications for axon guidance were not presented clearly enough. This is now clarified throughout the paper.

To further address this issue, we also now include new data on behavioural habituation to repeated tap, which is mediated by the chemical synapses of mechanosensory neurons. We show that MosSCI UNC-51 DN expressed in wt animals does not affect habituation, but defects in habituation caused by *rpm-1* (lf) are strongly suppressed by UNC-51 DN (Fig 3f, g). While habituation experiments were not requested by the reviewers, we think they provide further controls and independent evidence showing RPM-1 inhibits UNC-51 in a behavioural context. Given that UNC-51 DN expressed as Mos single copy does not affect axon guidance (Supplementary Fig 4), these findings also reveal another functional context in which RPM-1 effects on UNC-51 can be separated from effects on axon guidance. Restoring habituation defects towards normal behaviour would not be compatible with generalised disruption of axon guidance and growth. Thus, these behavioural results further support our model of reduced *unc-51* function suppressing *rpm-1* (lf) defects.

3) We have tested further autophagy mutants including *epg-8/Atg14* (Fig 7c, e, f) and *bec-1* (Fig S10), which regulate phagophore nucleation. Our results indicate that *rpm-1; epg-8* double mutants show suppression of defects in PLM axon termination, ALM axon termination and PLM synapse maintenance caused by *rpm- 1* (lf) (Fig 7c, e, f). *rpm-1; bec-1* double mutants also showed suppression of ALM termination and PLM synapse maintenance defects, but did not suppress PLM termination defects (Fig S10). These results now increase the number of autophagy mutants evaluated from 2 to 4 (not including unc-51) and provide further experimental support for our conclusion that RPM-1 inhibits UNC-51 to affect autophagy rather than axon guidance. We also provide new text in the Results that are more thorough and clear regarding this point.

The editor also noted several points we now address. These include:

1) We appreciate the editor noting that we accidentally used the same image for wt and *rpm-1* in Figures 2, 4, and S3. This has now been corrected with new images for both genotypes in Figures 4 and S3. Similar corrections were made for S5.

2) We provide new plots showing genetic outcomes for axon termination and synapse maintenance, as histograms with dots representing average outcomes from a single counting session. Where this type of plot becomes very complicated and impairs presentation clarity (*e.g.* Fig 2e, f – stacked histograms), we keep our original style of presentation. We ask that the editor and reviewers note

that we do not show data for individual worms, but rather show averages from single counting sessions (25-35 worms per count) as a single data point. This is noted in the Figure legends and Supplemental Methods section on statistics.

3) As per the editor's request, we now include a source file with raw data for all quantitative experiments in our manuscript. We also now include expanded mass spectrometry results for individual experiments in Table S1.

>>>

Reviewer #1 (Remarks to the Author):

Crawley et al. address the question of "how is autophagy regulated in neurons"? They perform a proteomics screen combined with genetic analysis in C. elegans to identify that the RPM-1 ubiquitin ligase negatively regulates the autophagy initiator UNC-51/ULK kinase to control axonal and synaptic development. The model generated is that RPM-1 targets UNC-51 for proteasomal degradation specifically within axon terminals to locally downregulate autophagy and allow for proper termination of axons in the developing nervous system. These findings are very intriguing and will provide significant insights for the neuronal autophagy field. Further, the authors uncover a new mechanism that defines crosstalk between the UPS and autophagy to coordinate cellular degradation in neurons. The data are of high quality and presented well. The following points, however , need to be addressed before the paper can be acceptable for publication:

>>>>
We thank the reviewer for their time in evaluating our manuscript, and their thoughtful comments. We also appreciate them highlighting the significant advances our work brings to the field of neuronal autophagy, and noting the quality of both our experiments and presentation. Below we respond to the reviewer's specific comments.

Major points:
1. Can the authors clarify the temporal sequence of events in their model? The model indicates that low autophagy supports termination and high autophagy is correlated with axon overgrowth. Therefore, in WT worms, is RPM-1 enriched at the axon terminus only at the time of termination? Have the authors checked earlier time points in development to examine whether RPM-1 levels are low in growth cones during the elongation phase, upstream of termination, to allow for higher autophagy to promote axon growth? In other words, are autophagy levels in the growth cone suddenly decreased upon arrival at the target? Perhaps higher levels of autophagy are needed during axon extension, but then levels need to decrease for termination to occur. Discriminating between these points would illuminate different roles for autophagy in promoting axon growth but inhibiting termination. How do these results reconcile with previous literature that loss of autophagy proteins decreases axon outgrowth in
the CNS (Dragich et al., eLife 2016, and others)?

>>>
We understand the reviewer's request for clarification of developmental events regarding our proposed model of RPM-1 and UNC-51 effects on autophagy. The reviewer's point that higher levels of autophagy could be required during axon outgrowth, while lower levels are required for termination, fits with our previous observations as well as those described in this manuscript. We clarify this point below further.

1) In the current study, overexpression of UNC-51 (the autophagy initiator) in wt animals resulted in failed PLM axon termination and synapse maintenance (Fig 4e, S5c, d), which is similar to *rpm-1* (lf) mutant phenotypes.

In a prior study, we described RPM-1 localization to the terminated axon tip, as well as the growth cone when the axon has reached its target area and begun a protracted growth cone collapse process (Borgen, et al., Development 2017). Out study showed that *rpm-1* mutants fail to properly collapse PLM growth cones, which results in failed axon termination. Thus, RPM-1 is an established regulator of growth cone collapse and axon termination. This prior study included extensive developmental time course analysis on RPM-1 localization, as well as axon termination and growth cone phenotypes caused by *rpm-1* (lf).

With regard to synapse defects, our prior work used extensive developmental time course analysis to show that synapses assemble normally in *rpm-1* mutants, but fail to be maintained resulting in retraction of the synaptic branch (Borgen et al, *Elife*, 2019). As part of this study, we also evaluated RPM-1 localization at presynaptic terminals across developmental time course. Our results indicate that RPM-1 is present at presynaptic terminals as early as we can detect them and is present at this subcellular location into adulthood.

We cited both these prior studies in our original manuscript, but we now make these points about prior developmental analysis more clear for readers with textual changes. We also refer to synaptic defects as “synapse maintenance” defects throughout our paper given that this is now the established most accurate definition of this phenotype as it pertains to *rpm-1* (lf) mutants.

We hope the reviewer agrees that our extensive body of prior developmental work (which was extremely difficult and time-consuming) addresses where and when RPM-1 functions, and suggests that RPM-1 is likely to affect UNC-51 in similar developmental contexts.

2) Regardless of this prior work, we do agree with the reviewer that studying effects of RPM-1 on UNC-51 and autophagosomes earlier in development could be informative. However, we think such questions might be better addressed in a future study more specifically designed to consider such questions fully and carefully. Given the extensive scale of this initial paper (which addresses both axonal and synaptic readouts), we think developmental studies are beyond the scope of this manuscript. Moreover, multiple reviewers have asked for experiments to address effects of RPM-1 and UNC-51 on axon guidance, biochemical experiments to test if UNC-51 is ubiquitinated by RPM-1, and further evaluation of more autophagy mutants (requested by this reviewer and addressed below). Thus, we opted to focus our time and energy on addressing these other major concerns that required extensive experimental revisions.

3) Regarding the paper by Dragich et al (*Elife*, 2016) and similar observations (Coupe et al, *Cell Metabolism*, 2012) indicating autophagy affects axon outgrowth, the reviewer makes a good point. We think our results are consistent with this prior work. First, we show *unc-51* mutants have premature axon termination. We use the term “premature axon termination” for consistency with our use of the term “failed termination” for overgrowth. This terminology is used for historical reasons based on the *C. elegans* literature. Nonetheless, it is reasonable to consider premature axon termination similar to reduced axon outgrowth. Thus, our results are consistent with Dragich’s and Coupe’s prior work. Moreover, our results indicate that failed termination (again simply terminology and could be considered the same as “overgrowth”) results from excess UNC-51 activity. Here again, our results are consistent with Dragich’s findings namely that excess autophagy, which occurs in *rpm-1* mutants or when UNC-51 is overexpressed, results in overgrowth/failed termination. We agree with the reviewer that RPM-1 inhibition of UNC-51 and autophagy is likely to be part of the switching-off of the outgrowth process and thus the promotion of termination.

To further address this, we adjusted the text in our manuscript to make this point more clearly with the following statement in the Results:

"ALM axons in unc-51 mutants frequently displayed premature termination…. This result is consistent with prior studies in mice, which showed that impairing autophagy reduces axon outgrowth[24, 25].

We also thank the reviewer for noting the Dragich paper, which we accidentally forgot to cite in our initial version of the paper. We have now given this citation the proper representation it warrants at multiple points in our manuscript.

2. The failed termination phenotype with the rpm-1 mutant is not completely suppressed by mutations in atg-9 (or epg-6). These results indicate that either (1) alternative mechanisms are also at play, independent of autophagy, or (2) the atg-9 mutant does not completely block autophagy. Can the authors reconcile these points? Recent literature in mammalian cells indicates that loss of atg-9 from the TGN does not block basal autophagy, but only stress-induced autophagy (De Pace et al. PLOS Genetics 2018). Further, Atg9 may have roles in axon outgrowth independent of autophagy (Yamaguchi et al. Autophagy 2017). Thus, mutants of Atg9 may confound interpretations. Can the authors use atg5 or atg7 mutants to block autophagy or are there lethality concerns?

>>>
We agree that incomplete suppression of *rpm-1* loss of function phenotypes by *atg-9* and *epg-6* autophagy mutants could indicate potential contribution from alternative mechanisms. This is also possibly because these mutants could be hypomorphic, as many autophagy mutants in *C. elegans* are, due to lethality issues. Alternatively, because ATG-9 and EPG-6 act further down in the autophagy process than UNC-51/ULK milder suppressor effects might occur.

To further address this, we include new data for two more autophagy mutants, *epg-8/Atg14* (Fig 7c, e, f) and *bec-1* (Fig S10). Both EPG-8 and BEC-1 affect phagophore nucleation, a step in autophagy we did not test in our original manuscript (Fig 7a). *rpm-1; epg-8* double mutants show suppression of PLM and ALM axon termination defects and PLM synapse maintenance defects (Fig 7c, e, f). *rpm-1; bec-1* double mutants show suppression of ALM termination defects and PLM synapse maintenance defects, but do not suppress PLM termination defects (Fig S10). Overall, we now show that two autophagy mutants, *atg-9* and *epg-8*, suppress all defects caused by *rpm-1* (lf) we evaluate (Fig 7). Two other autophagy mutants, *bec-1* and *epg-6*, suppress 2 of the 3 defects evaluated (Fig S10). We point out that inability of *bec-1* and *epg-6* (lf) to suppress PLM termination defects caused by *rpm-1* (lf) might be due to maternal contribution for *bec-1* (could only be evaluated in the F1 generation due to lethality issues), and the nature of the *epg-6* allele which might be a weaker loss of function than *atg-9* or *epg-8* alleles. Regardless, this new data collectively provides extensive support for our conclusion that RPM-1 restrains UNC-51 to inhibit autophagy.

Other evidence for this conclusion stems from proteomic, biochemical and genetic effects of RPM-1 on UNC-51 (Fig1-5) and direct evidence that RPM-1 affects autophagosomes (Fig 7, 8).

We think this collective body of data argues strongly for RPM-1 inhibition of UNC-51 affecting autophagy. However, we also provide several other pieces of evidence we think rules out (with reasonable confidence) the possibility of the established UNC-51 role in axon guidance.

1) We show loss of function in *unc-14* (a known UNC-51 binding proteins that functions with UNC-51 in axon guidance (Ogura et al 1997, Lai & Garriga 2004)) does not suppress *rpm-1* (lf) phenotypes (Figures 2E, 2F and 3C).

2) Loss of function in the netrin receptors *unc-40/DCC* and *unc-5* did not suppress *rpm-1* (lf) phenotypes (Fig S11). UNC-5 is the netrin receptor identified as an axon guidance molecule whose normal localization in neurons is controlled by UNC-51 (Ogura & Goshima Development 2006).

3) Most importantly, we extensively deploy an UNC-51 dominant negative (UNC-51 DN) construct that is known to inhibit the autophagy functions of UNC-51 (Chan… Tooze, *Mol Cell Biol*, 2009; Ogura et al, Genes and Dev, 1994). We show Mos single copy expression of UNC-51 DN in mechanosensory neurons strongly suppressed axon termination, synapse maintenance and habituation (new data, see paper for details and reasons for including these new experiments) defects in *rpm-1* (lf) mutants (Fig3d-g; S3). New results show UNC-51 DN does not cause any discernible axon guidance defects (Supplementary Fig 4). We apologize to the reviewer that the way we presented our data in our original manuscript did not make it clear that this is a dominant negative UNC-51 construct.

Regarding the reviewer's concerns about using *atg-9* mutants. We draw the reviewer's attention to recent work in *C. elegans* (Stavoe et al, *Dev Cell* 2016) that convincingly shows ATG-9 affects neuronal autophagy and synapse development. Given the arguments above (in particular further evaluation of two more autophagy mutants) and this prior work, we think it is reasonable to include ATG-9 and consider it a relevant player in autophagy. Nonetheless, we appreciate the reviewer pointing out interesting recent literature showing non-autophagy related roles for ATG-9. We will certainly bear this in mind as we proceed with this line of research.

3. To clarify the identity of the structures in Fig. 7G, higher resolution images are needed.

>>>
We understand the reviewer's point. First, some clarification. In the original legend, we did not present this data are carefully as needed. Shown to the right of each image are zoomed in images of the regions that are highlighted (hatched boxes). Perhaps this addresses the reviewer's concern.

Second, we agree that higher magnification images could be valuable. However, these are the highest resolution images and magnification that were possible under the constraints of this confocal imaging set up and particular neuron morphology. We are visualizing puncta using a single copy GFP::mCherry::LGG-1 transgene which maximizes physiological relevance, but limits the level of signal. Moreover, we are imaging this marker in an intact live animal *in vivo*. Thus, there are some pragmatic trade-offs that were made between magnification and speed of imaging. For live imaging, the animals are anesthetized but not perfectly immobilized so shift and twitch during prolonged imaging can create issues. To capture the full PLM neuron morphology plus the ALM and AVM cell bodies (anatomical reference points) in a single stack there are considerable magnification and imaging speed constraints, particularly given the imperfect immobilization. While we have tried, this is also an issue that prevents us from using super-resolution imaging on live, anesthetized animals. Finally, because this is a single fusion protein, GFP::mCherry::LGG-1, it is possible FRET occurring between GFP and mCherry further limits the intensity of signals. Again because we use single copy insertion this might heighten the effects of FRET. We hope the reviewer agrees that we have done our best to deal with this issue given the limitations of our approach and system.

It is also noteworthy that this is the first time any group has imaged autophagosomes in mechanosensory neurons. Perhaps in the future with better optimization and more in-depth study, we might be able to use higher resolution imaging technologies to study these structures.

4. There is no direct evidence for direct proteasomal degradation in Fig. 5. Can the authors use MG132 in the SH-SY5Y model? Alternatively, can they exclude altered localization of UNC-51/Ulk1 in the absence of RPM-1 in the worm model?

The reviewer makes two good points.

To address this, we have turned to HEK 293 cell biochemistry to show that PAM (RPM-1 human ortholog) is sufficient for polyubiquitination of ULK1 and that inhibiting the proteasome with MG132

increases PAM ubiquitination of ULK1 (Fig 5j). This is further direct biochemical evidence that PAM ubiquitinates ULK1 in a proteasome-dependent manner. 293 cells are necessary because SH-SY5Y cells cannot be transfected with high enough efficiency for this type of complicated multi-component biochemistry.

We have not ruled out the possibility that RPM-1 affects UNC-51 trafficking, but our new cell-based biochemical data now provides further evidence indicating RPM-1 is likely to ubiquitinate UNC-51. This is also supported by all our proteomic, biochemical and genetic results from worms.

As the reviewer requested, we did try to see how the proteasome inhibitor MG132 affects ULK1 protein levels in SH-SY5Y and 293 cells. We also tried testing further doses and extended times for cycloheximide treatment. Unfortunately, ULK1 is very stable in cultured cells, and we were unable to observe effects of MG132 on ULK1 levels. We also could not increase turnover of ULK1 beyond what is shown in our existing data in Figure 5h, i.

We think our data overall makes it unlikely this is an effect of RPM-1 on UNC-51 trafficking. However, if the reviewer would like we can add this caveat as a sentence in the paper.

Minor points:

1. Figure 7: mCherry signal in the axon terminus is barely visible in WT images. Can the authors correct this?

>>>

The reviewer is correct that very little mCherry (and GFP) signal is present at the axon tip of wt animals in Figure 7. The primary reason the mCherry signal is very weak is because there is consistently very minimal accumulation of LGG-1 at the axon tip. The most likely reason this appears more pronounced for mCherry fluorescence than GFP (as both should be visible in low acidity autophagosomes where GFP fluoresces) is that mCherry has around 4 times weaker brightness per molecule than GFP under practical multichannel confocal imaging conditions using *C. elegans* (Heppert… Goldstein, Molec Bio Cell, 2016).

We chose not to manipulate the mCherry::LGG-1 image because the level of mCherry::LGG-1 signal in the ALM cell body is already quite high as shown (see Figure 7g; wt middle panel; upper left side of image).

2. Figure 1F: Authors should make a table listing the identity of proteins isolated in different detergent conditions.

>>>

We appreciate the reviewer's desire to see the identity of putative RPM-1 binding proteins identified by proteomics under different detergent conditions. However, we consider showing the entire proteomic dataset beyond the scope of this manuscript. This is because these proteomic hits have not been validated or verified in anyway. Some are also the focus on ongoing projects involving different researchers.

To address this, we have removed commentary on this data and the results (original Figure 1f, now coIP data).

Nonetheless, the reviewer's point is well taken. To further address the reviewer's comment, we now show a new Table (Table S1) with the detergent extraction conditions and primary proteomic data for all known RPM-1 binding proteins (*i.e.* GLO-4, PPM-2 and RAE-1), ubiquitin ligase complex components (*i.e.* FSN-1 and SKR-1), UNC-51 and UNC-14 which are all relevant to this manuscript.

3. Figure 7e: typo in y-axis.
>>>
The reviewer is correct we had a typo. We changed the axis label to be consistent with our axis label from earlier figures.

4. Figure 9: Model on the right side is unclear. Are levels of autolysosomes in the soma the same in WT vs rpm-1 mutant (as it is depicted in the model), even though more autophagosomes are generated? Is this model based on a quantitation of autolysosome density in the soma?

>>>
A good point. We did not directly test this question. However, it is unlikely we will be able to detect these differences as large amounts of autolysosomes are present even in the cell bodies of mechanosensory neurons in wt animals (see Fig7g; for wt extensive mCherry::LGG-1 signal, but very little GFP::LGG-1 signal in ALM cell body; similar for rpm-1).

Our paper is focused on autophagy and autophagosomes. Therefore, we have not attempted to address the question of autolysosomes beyond our qualitative assessment that there were not noticeable differences in autolysosome levels (mCherry::LGG-1 signal alone) in cell bodies of ALM (Figure 7g) and PLM neurons (not shown) for wt and *rpm-1* mutant animals. Presumably this is because changes in autophagosomes in axonal compartments might not impact gross autolysosome number or size due to the large number of autolysosomes already present in the cell bodies of mechanosensory neurons in wt animals.

Our observations are similar to observations in a prior *C. elegans* study, which showed that autolysosomes (inferred from mCherry::LGG-1) greatly outnumber autophagosomes in neuronal cell bodies of wt animals (Chang… Hansen, Elife, 2017).

Reviewer #2 (Remarks to the Author):

The manuscript entitled “Autophagy is Inhibited by Ubiquitin Ligase Activity in the Nervous System” by Crawley et al. highlights a previously undescribed relationship between the E3 ubiquitin ligase RPM-1 and the serine-threonine kinase UNC51 that affects axon growth. Although the data are intriguing, a few critical controls are missing and the data regarding the role of autophagy in the phenotype is preliminary.

>>>
We appreciate the reviewer’s thorough and thoughtful comments, as well as their time in evaluating our manuscript. We are glad that they think our findings are interesting, and hope revisions to the paper they requested will further increase their enthusiasm for our work. Below we respond to specific comments.

Major concerns are listed below:
1. Figure 1 nicely shows the trapping of UNC-51 by ligase-defective RPM-1 using an unbiased proteomics approach. The authors should provide biochemical evidence that UNC-51 is indeed a substrate whose turnover is regulated in an RPM-1 dependent manner. For example, based on the model, one would predict that proteasome-mediated inhibition should result in the accumulation of ubiquitinated forms of FLAG::UNC-51, and that rpm1 mutant worms would show elevated steady

state levels of FLAG::UNC-51 with loss of the proteasome inhibitor effect. Biochemical data would help to strengthen the conclusions drawn from the imaging based data presented in figure a-f. [Comment: the authors may consider presenting the data in Figure 5 and 6 after Figure 1 to better set up the rationale for the genetic experiments and improve the flow of the manuscript].

>>>
We agree with the reviewer that direct biochemical evidence showing RPM-1 can ubiquitinate UNC-51 would be valuable. We opted to address this concern using cell-based experiments with orthologous human proteins (i.e. PAM and ULK1) for 3 reasons. First, drugs that block the proteasome are more easily deployed and effective in cells than worms. Second, biochemically detecting direct ubiquitination is technically more straightforward in cell-based assays than *C. elegans*. Finally, by using human PAM and ULK1 (human orthologs of RPM-1 and UNC-51) we add further translation as well as mechanistic content to our paper.

Using cell-based biochemistry in HEK 293 cells, we now show that human PAM is sufficient for polyubiquitination of ULK1 (Figure 5j). Importantly, PAM ubiquitination of ULK1 is further heightened when the proteasome is inhibited with MG132.

These new data further support our model that PAM/RPM-1 directly ubiquitinates ULK1/UNC-51 to control its protein level via proteasome mediated degradation. Moreover, these biochemical results are consistent with our findings that local levels of UNC-51 (Figure 5b, c) and autophagosomes (Figure 7g, h) are increased in axonal compartments when RPM-1 ubiquitin ligase activity is absent. These changes occur in the same locations where RPM-1 is normally found (Figure 6).

We hope the reviewer agrees that these new experiments increase the scope of our study, provide further mechanistic depth, and significantly improve our manuscript.

2. The data in Figure 5h shows an increase in steady state levels of ULK1 upon shRNA-mediated silencing of PAM, but as the difference is eliminated after CHX treatment the results suggest that the difference stems from increased translation, not a defect in turnover. This experiment needs to be performed in a more rigorous manner – ideally with calculation of ULK1 half-life in NT control and shPAM cells. As presented, the data do not support the conclusions.

>>>
We understand the reviewer’s concern.

Traditionally, the translation inhibitor cycloheximide is used in studies with ubiquitin ligases to increase substrate turnover and draw out effects of ubiquitin ligase activity on substrate protein levels. However, altering ubiquitin ligase activity also often effects the levels of substrates without blocking translation, as we observed for ULK1 levels with PAM knockdown.

Our results indicate that ULK1 is a particularly stable protein that shows little turnover when translation is blocked with cycloheximide. For a comparison, please see Desbois et al (JBC 2018) where NMNAT2 (another PAM substrate) turns over to a much greater and faster extent than ULK1. SH-SY5Y cells have very slow turnover of ULK1 presumably because this is advantageous for this cell line.

However, the effects of PAM KD on levels of ULK1 do persist even with cycloheximide treatment for 1 and 2 hours. These results as well as effects in DMSO are all consistent with PAM affecting ULK1 protein turnover, and ULK1 being a particularly stable protein that is not turned over rapidly.

To further address this concern, we took two steps. First, we evaluated how different times and doses of cycloheximide treatment affect ULK1 in two cell types, HEK 293 cells and SH-SY5Y cells. Unfortunately, we were not able to increase turnover of ULK1 beyond what is shown already in wt SH-SY5Y cells (Fig 5h, i). As a result, it was technically difficult to accurately assess ULK1 protein

half-life in wt SY-SH5Y cells. Second, we adjusted the language in the text to handle this issue more carefully and to be less conclusive:

"Quantitation showed ULK1 protein levels were significantly increased in PAM knockdown cells (Fig. 5i). This effect could also be observed when translation was inhibited with cycloheximide (Fig. 5h, i). Cycloheximide was applied to facilitate turnover of ULK1 and possibly accentuate effects of PAM knockdown on ULK1 levels, but this did not occur. This suggests that ULK1 is quite a stable protein in these cells, and is notably different from the PAM substrate NMNAT2 which we showed turns over rapidly[34]. Nonetheless, these results are consistent with PAM restricting ULK1 proteins levels in human neuroblastoma cells."

We understand the Reviewer's concern that the effect of cycloheximide shows minimal decrease in ULK1 control cells while resulting in a larger decrease in ULK1 levels in the PAM knockdown cells after 4 hours cycloheximide compared to PAM KD in DMSO. We think this might arise from compensatory processes that might be initiated to affect ULK1 levels with prolonged lack of PAM activity. This is reasonable, as we used puromycin selection for 6 days (see Methods) after lentiviral infection of cells with PAM KD constructs. This ensures all cells have PAM KD, but is a lengthy period of time that could result in compensatory mechanisms.

We hope the reviewer agrees; rather than focus on this issue further, we thought it reasonable to invest our time and effort extensively in further cell-based assays to show that PAM is sufficient to ubiquitinate ULK1, and that PAM ubiquitination of ULK1 is increased when the proteasome is blocked with MG132. This new data provides a biochemical mechanism to explain why ULK1 levels are elevated in PAM KD SH-SY5Y cells, and provides further mechanistic insight into why UNC-51 levels are elevated in axonal compartments in *rpm-1* (lf) mutants and *rpm-1 LD CRISPR* animals (Fig 5a-f; S7).

3. The results of the genetic experiments presented in Figures 2-5 are intriguing, but in most cases the phenotype of rpm1 mutants reverts to that of the unc-51 mutant. Can the authors exclude the possibility that problems with axon growth and guidance in the unc-51 mutants are upstream of the defects caused by loss of rpm-1 and prevent the manifestation of the defects caused by loss of rpm-1 (and are unrelated to a failure to degrade UNC-51)? Perhaps crossing unc-51 mutants with another genetic mutant that causes failed termination or hooking (similar to rpm-1 mutants) to see if those phenotypes persist in double mutants would help to resolve the issue.

>>>
The reviewer makes an important point.

We address this with new written text to clarify this throughout the manuscript, and include new data on axon guidance in mechanosensory neurons (Supplementary Fig 4) to directly address this concern. As we discuss below, several independent results indicate UNC-51 functions via distinct molecular mechanism to affect axon guidance compared to its role downstream of RPM-1 in axon termination and synapse maintenance.

1) The possibility that effects of UNC-51 on axon termination and synapse maintenance might be complicated by UNC-51 effects on axon guidance was best addressed by MosSCI expression of an UNC-51 dominant negative (DN) point mutant in mechanosensory neurons of *rpm-1* mutants. UNC-51 DN strongly suppressed defects in axon termination (Fig S3) and synapse maintenance (Fig 3d,e) caused by *rpm-1* (lf). Our new results now show that UNC-51 DN does not affect axon guidance in mechanosensory neurons (Supplementary Fig 4). This indicates that UNC-51 DN can strongly suppress *rpm-1* phenotypes in both axon termination and synapse maintenance without causing any discernible axon guidance problems. Thus, the UNC-51 DN is an extremely informative reagent that allowed us to more cleanly dissect suppression of *rpm-1*, and segregate effects of

UNC-51 on synapse maintenance and axon termination from its well-established role in axon guidance.

We note that in our original manuscript, we did not ideally present these results, as we referred to the UNC-51 DN as UNC-51 kinase-dead. While this reagent is catalytically inactive, prior work has shown it functions as a dominant negative to affect UNC-51 function in autophagy (Chan.. Tooze, *Mol Cell Biol,* 2009; Ogura et al, *Genes Dev*, 1994). We have adjusted our presentation of this reagent throughout the paper to deal with this issue more clearly.

2) Data included in our original manuscript (Fig 2 and 3), which is now presented more clearly and thoroughly to address this issue, shows that axon termination defects caused by *rpm-1* (lf) are not suppressed in *rpm-1; unc-14* double mutants. UNC-14 is a known UNC-51 binding protein that functions with UNC-51 to regulate axon guidance (Ogura et al 1997; Lai & Garriga 2004). Thus, suppression of *rpm-1* (lf) by *unc-51* (lf) and UNC-51 DN, but not *unc-14* (lf), provides further evidence to separate the role of UNC-51 in axon termination and synapse maintenance downstream of RPM-1 from its known function in axon guidance.

3) Previously, UNC-51 and UNC-14 were shown to affect axon guidance by regulating Netrin receptor trafficking (Ogura & Goshima Development 2006). Therefore, we also tested the genetic relationship between *rpm-1* and two netrin receptors, *unc-5* and *unc-40/DCC*. Our results indicate that defects in ALM axon termination, PLM axon termination and PLM synapse maintenance caused by *rpm-1* (lf) are not suppressed in *rpm-1; unc-5* or *rpm-1; unc-40* double mutants (Fig S11). In fact, defects in synapse maintenance and ALM axon termination are increased in *rpm-1; unc-40* double mutants compared to single mutants (Fig S10B, C), which suggests RPM-1 acts in parallel to this Netrin receptor to regulate axon termination and synapse development.

4) The reviewer's valuable point about axon guidance highlighted our need to do more extensive experiments with UNC-51 DN. While not directly requested by the reviewer, we thought it valuable to include new data on behavioural habituation that is mediated by the glutamatergic chemical synapses of the mechanosensory neurons. Our new results show that expression of UNC-51 DN (which does not affect axon guidance in mechanosensory neurons (Fig S4)) does not impair habituation on a wt background, but strongly suppresses habituation defects caused by *rpm-1* (lf) (Fig 3f, g). These behavioural results are consistent with our findings on *rpm-1* and UNC-51 DN in PLM synapse maintenance (Fig 3). Indeed, restoring habituation defects towards normal behaviour would not be compatible with generalized disruption of axon guidance and growth. As a result, these behavioural results further support our model of reduced *unc-51* function suppressing *rpm-1* (lf) defects. Overall, this new data strengthens our conclusions, provides further experiments to highlight the value of the UNC-51 DN reagent, and increases the impact of our study by adding a whole animal behavioural readout.

Taken as a whole, we hope the reviewer agrees that our existing data, new data, and textual alterations carefully and thoroughly deal with this important consideration.

If the reviewer deems it necessary. We are happy to add further text stating that we cannot entirely rule out the possibility that effects of UNC-51 on axon guidance might be relevant in the context of axon termination and synapse maintenance.

4. What happens to the premature termination of ALM/PLM neurons, overextension of PLM neurons and PLM branching defects observed in rpm-1, unc-51 double mutants upon overexpression of WT UNC-51 or KD UNC-51? This is an important control.

>>>
We understand the reviewer's interest in knowing how all phenotypes for both PLM and ALM neurons are affected by wt UNC-51 and UNC-51 DN (previously referred to as UNC-51 KD) in *rpm-1; unc-51* double mutants. The reviewer's comments are valuable on several levels, and there is

some confusion that arose from our style of presentation that we have rectified in our revised manuscript. Simply put, UNC-51 KD is the same MosSCI construct in rescue experiments as when we look for suppression in *rpm-1* mutants. We now refer to this reagent as UNC-51 DN when expressed in *rpm-1* mutants and UNC-51 KD when we are testing *unc-51* rescue. We think this makes it clearer how we are using this reagent in different genetic contexts. Below, we respond further with several points.

1) A few points of clarification. First, we ask that the reviewer note that only those experiments annotated as UNC-51 overexpression (OE) involved extrachromosomal arrays. In these cases, UNC-51 is always expressed in wt animals (Fig 4d, e; Fig S5c, d). Second, in all other cases UNC-51 KD was only expressed using Mos single copy insertion. We now make sure to note whenever we used MosSCI expression throughout the Results. Importantly, Mos single copy expression of UNC-51 or UNC-51 KD would not be considered an overexpression experiment when done on an *unc-51* (lf) background. We have checked our manuscript to ensure we do not use the term 'overexpression' for MosSCI constructs.

2) In terms of presenting axon termination, axon overextension and premature termination defects for ALM and PLM data with rescue experiments, we originally considered doing this. However, we decided not to include this data, despite its clear support for our conclusions, because we felt it over complicated presentation of our data, and might labour the point. Rather we opted to present our data to best facilitate the reader digesting the most important observation: Rescue of suppression of primary *rpm-1* phenotypes in *rpm-1; unc-51* double mutants when UNC-51, but not UNC-51 KD, are expressed in these animals.

While we did not show further phenotypic data on axon overextension and premature termination for PLM neurons, in all cases we observed the expected patterns of rescue by UNC-51 MosSCI in the *rpm-1; unc-51* double mutant: presence of premature terminated axons was completely rescued for both ALM and PLM, and overextension of PLM neurons was strongly rescued. Conversely, expression of UNC-51 KD MosSCI in the *rpm-1; unc-51* double mutant did not suppress short axon phenotypes for ALM or PLM neurons and also did not significantly suppress overextension of the PLM. All of this data is now included in the source file that accompanies our manuscript, and was requested by the editor.

Although it might disrupt the flow and clarity of our presentation for readers, we can include this phenotypic data for rescue experiments as a supplemental figure if the reviewer thinks this is essential.

5. The authors demonstrate that overexpression of UNC-51 causes failed termination in PLM axons and branching defects – does it cause any overextension or premature termination? What is the effect of UNC-51 overexpression on ALM axons?

>>>
We understand the reviewer's interest in the effect of UNC-51 overexpression in wild-type animals on other phenotypes not shown. We did not show this data in order to focus the reader on the phenotype most important for understanding this story, *i.e.* failed termination (hook) defects and synapse maintenance/synaptic branch defects.

We did not observe any significant overextension of the PLM neuron with UNC-51 overexpression (not shown). For premature termination in PLM neurons, we did see significant but low levels of this phenotype with UNC-51 overexpression (not shown). This indicates, similar to phenotypes observed in *unc-51* (lf) mutants, that there is a fine balance of UNC-51 activity which is important for overall axon growth, which we note in the manuscript. The data presented support a model that excess UNC-51 activity can result in failed termination and failed synapse maintenance, similar to what is seen in the *rpm-1* (lf) mutants. However, it is conceivable that artificially high levels of UNC-51 early

in development of an axon could also sometimes cause catastrophic events to prevent correct outgrowth, via known effects on guidance mechanisms.

We did not show data for ALM neurons because we saw a trend towards failed termination defects with two out of five lines giving over 25% termination defects, but two lines giving negligible defects. This high variability between different arrays (which does sometime happen) caused us to leave this data out of the paper. Furthermore, multiple observations in our lab from rescue and overexpression experiments indicate that the *mec-7* promoter displays weaker expression in ALM neurons compared to PLM neurons (not shown).

Although it may slightly disrupt the flow and clarity of our results, we are happy to present the data on these findings in a Supplementary Figure, if the reviewer does not deem our response here sufficient. As noted above, this data is present in the source file we now provide for all quantitative data in this paper, as requested by the editor.

6. The authors claim that excessive autophagy in rpm-1 mutants accounts for the defects in axon termination. This is based on the reversal of the phenotype in atg9 mutants and the observation of increased LGG-GFP fluorescence in neurons. I have several concerns regarding the conclusion. First, a recent publication demonstrates that disrupting Atg9a in mice causes axon guidance defects that are not observed in other canonical autophagy knockouts (i.e. atg7-KO, atg16l1 KO), but are similar to those caused by ULK1/2-deficiency (Yamagichi et al. Autophagy 14:764-777, 2018). Therefore, the observation that disrupting atg9 but not epg6 reverses the phenotype in rpm-1 could suggest that the phenotype is unrelated to autophagy. Moreover,just because autophagy (or at least LGG-GFP) appears to be increased in rpm-1 mutant worms does not mean that this increase is responsible for the phenotype. Thus, the conclusion that the axon termination defects in rpm-1 mutants are caused by excessive UNC-51 dependent autophagy is premature. Additional studies would have to be performed (e.g. examining the effect of disrupting other autophagy genes on the rpm-1 mutant phenotype) to convince readers of the connection (or lack thereof) with autophagy. If other autophagy genes do not rescue, then the authors should tone down their conclusions regarding the regulation of autophagy by rpm-1 and instead focus on its regulation of UNC51 function (whether autophagy-related or unrelated), using the results of atg9 and other autophagy mutants to help delineate the pathway.

>>>
We recognise the reviewer's hesitation in our conclusion that increased autophagy is a downstream mechanism mediating axon termination defects in *rpm-1* mutants. However, we must point out that both PLM synapse maintenance defects and failed termination defects in ALM neurons were suppressed by both *epg-6* (Fig S10b, c) and *atg-9* (lf) (Fig 7e, f). Lack of *epg-6* suppression for failed termination defects in PLM neurons (Fig S10a) might reflect varying demands for autophagy in different neurons or phenotypic settings, or could indicate that this allele is a slightly weaker loss of function. Nonetheless, our data do indicate that *epg-6* (lf) is capable of suppressing *rpm-1* (lf) in at least 2 of 3 phenotypes evaluated.

To further address this concern, we tested two other autophagy mutants, *epg-8* (Fig 7c, e, f) and *bec-1* (Fig S10). Both EPG-8/ATG14 and BEC-1 function relatively early in the autophagy process by affecting phagophore nucleation (Fig 7a). We observed suppression of ALM termination defects, PLM termination defects and PLM synapse maintenance defects in *rpm-1; epg-8* double mutants (Fig 7c, e, f). For *rpm-1; bec-1* double mutants, we observed suppression of ALM termination defects and PLM synapse maintenance defects, but not PLM termination defects (Fig S10).

Overall, we now show that two autophagy mutants, *atg-9* and *epg-8*, suppress all defects caused by *rpm-1* (lf) evaluated (Fig 7c, e, f). Two other autophagy mutants, *bec-1* and *epg-6*, suppress 2 of the 3 defects evaluated (Fig S10). We point out that inability of *bec-1* and *epg-6* (lf) to suppress PLM termination defects caused by *rpm-1* (lf) might be due to maternal contribution for *bec-1* (could only be evaluated in the F1 generation due to lethality issues), and the nature of the *epg-6* hypomorphic

allele which might be a weaker loss of function than *atg-9* or *epg-8* alleles. PLM termination might also be more sensitive to particular autophagy mutants. Regardless, this new data collectively provides further support for our conclusion that RPM-1 restrains UNC-51 to inhibit autophagy. Other evidence for this conclusion stems from proteomic, biochemical and genetic effects of RPM-1 on UNC-51 (Fig1-5) and direct evidence that RPM-1 affects autophagosomes (Fig 7, 8).

In response to the reviewer's concern about using *atg-9* mutants to draw conclusions about autophagy, we bring their attention to research published with *C. elegans* (Stavoe et al. Mol. Cell 2016). This study convincingly linked ATG-9 with autophagic activity along with various other autophagy proteins, including UNC-51, in controlling synapse development in AIY interneurons. However, we also appreciate the interesting recent literature showing non-autophagy related roles for ATG-9 in axon outgrowth.

To further address this issue, we refer the reviewer back to our earlier statements that discuss why UNC-51 effects on axon termination and synapse maintenance that are regulated by RPM-1 are molecularly and genetically distinct from its roles in axon guidance. The same is likely to be the case for ATG-9, since we do not observe axon guidance defects in the mechanosensory neurons of *atg-9* mutants (data not shown).

Reviewer #3 (Remarks to the Author):

The manuscript by Crawley et al reports the identification of C. elegans ATG kinase UNC-51 as an in vivo substrate of the RPM-1 ubiquitin E3 ligase. Previous studies from the same lab have characterized a number of proteins that bind RPM-1, from an early proteomics analysis of wild type RPM-1. Here, the authors designed a ligase-dead RPM-1 to enrich candidate ubiquitination substrates. Together, with the use of a protein-G::streptavidin binding peptide purification tag, they obtained many more RPM-1 binding proteins. UNC-51 kinase is a top hit among those showing enrichment for ligase-dead RPM-1. They addressed the in vivo relevance of UNC-51 in neuronal development regulated by RPM-1, employing a set of well-established genetic assays on the axon termination and synapse pattern of the mechanosensory neurons. Most of the data support their conclusion that RPM-1 acts upstream of UNC-51 and inhibits UNC-51 abundance in axon ends. They also show that in human neuroblastoma cells ULK1 protein levels are regulated by the human protein PAM, the homolog of RPM-1. Given the role of ATG kinase in autophagy, they addressed whether UNC-51's role involved autophagy process. Through quite extensive genetic interaction and cellular marker analyses, they conclude that RPM-1 restricts autophagosome formation at axon termination site.

The strength of the paper includes the improved proteomics analyses that reveal new binding partners of RPM-1, the functional dissection of UNC-51 in RPM-1 mediated neuronal development and maintenance, and the implication of autophagy being downstream of UNC-51 and RPM-1 interaction. The amount of data is overwhelming, and the technical range is impressive. An obvious weakness is the lack of direct evidence that UNC-51 is ubiquitinated by RPM-1. The effects of RPM-1 on autophagosomes are subtle. Additionally, there are some over-stated conclusions throughout the manuscript, and some statements need further clarification.

>>>
We appreciate the reviewer noting the significance and quality of our study. We also appreciate their time in carefully evaluating our work.

We now include new data showing that PAM (the RPM-1 human ortholog) is sufficient to polyubiquitinate ULK1 (human UNC-51 ortholog) and that this is increased with pharmacological inhibition of the proteasome (Fig 5j).

The effects of RPM-1 on autophagosomes might appear subtle for several reasons. 1) We opted to use single copy transgenes for all our autophagy reporters. In one case, ATG-9::GFP, we use a CRISPR reagent where endogenous ATG-9 is tagged. While these reagents increase physiological relevance, they also undoubtedly make our imaging less sensitive. 2) In the case of the GFP::mCherry::LGG-1 reagents, FRET could occur between the GFP and mCherry tags thereby further reducing signals. 3) It is reasonable that effects of RPM-1 on autophagosomes could be affected by other mechanisms that might compensate for loss of RPM-1. 4) Furthermore, it is important to note that our quantitative methodology did not involve background normalization for autofluorescence which is quite high at green wavelengths in *C. elegans* tissue. This background reduces the strength of relative changes. We chose to quantify our data as absolute raw intensities thereby minimizing image processing and keeping our assessment as close to direct measurement visualized by eye as possible.

While the reviewer's point is extremely well taken, we hope they agree that these are reasonable compromises given our goal of using *C. elegans* to its maximum potential to provide the most physiologically relevant, *in vivo* findings we can.

Specific comments:

-Fig 1d , Table 1 and the text on pg 4-5: the authors state 'More importantly, our LC-MS/MS analysis identified a striking number of novel RPM-1 interactors, and putative ubiquitination targets that were enriched in RPM-1 LD samples compared to wt RPM-1 (Fig. 1d).'

However, the complete list of proteins in Fig 1d. is not included in the manuscript. This data omission may be an oversight, and certainly reduces the level of enthusiasm for their claimed success of their proteomics.

>>>
The reviewer makes a valuable point.

This was not an oversight. We agree with the reviewer that our language overstated the amount of proteomic data we actually show in our manuscript. Therefore, we have adjusted our language to be more accurate. We have also removed the data shown in our prior Figure 1f summarizing outcomes and including information on different detergent conditions used for extraction. Given that this manuscript is about RPM-1 inhibition of UNC-51 and autophagy, we think our overall proteomic dataset is better left to future manuscripts.

To further address this issue, we provide a new Table (Table S1) with the detergent extraction conditions used in each experiment and all primary proteomic data for all known RPM-1 binding proteins (*i.e.* GLO-4, PPM-2, RAE-1), ubiquitin ligase complex components (*i.e.* FSN-1 and SKR-1) and the new proteins we identified enriched in RPM-1 LD samples: UNC-51 and UNC-14.
We do not disclose the complete lists of RPM-1 binding proteins identified in our samples because they are the basis for multiple ongoing studies that are projects being performed by different authors. Moreover, none of this proteomic data has been validated.

-pg 6-7: the authors drew a strong conclusion'Thus, in rpm-1; unc-51 double mutants the unc-51 (lf) phenotype fully dominates, which indicates RPM-1 functions as an upstream inhibitor of UNC-51.'.

Given the known role of unc-51 in axon guidance, presumably early development, a caveat to this interpretation is that the two genes may function in different phases of axon development and

maintenance. This might be a difficult issue to resolve solely based on snap-shot observations on axon morphology.

>>>
The reviewer makes an important point.

We address this concern with new text to clarify this issue throughout the manuscript, and include new data on axon guidance in mechanosensory neurons (Supplementary Fig 4) to directly address this point. As we discuss below, several independent results indicate UNC-51 functions via distinct molecular mechanism to affect axon guidance compared to its role downstream of RPM-1 in axon termination and synapse maintenance.

We refer the reviewer to two of our prior studies for complete developmental analysis of *rpm-1* function and RPM-1 subcellular localization with regard to axon termination and growth cone collapse (Borgen et al, *Development*, 2017), and synapse maintenance (Borgen et al, *Elife*, 2019).

1) The possibility that effects of UNC-51 on axon termination and synapse maintenance might be complicated by UNC-51 effects on axon guidance was best addressed by MosSCI expression of an UNC-51 DN reagent in mechanosensory neurons of *rpm-1* mutants. We apologize that this was previously annotated as UNC-51 KD, when in fact it is known to function as a dominant negative reagent that can affect autophagy (Chan… Tooze, *Mol Cell Biol*, 2009; Ogura et al, Genes and Dev, 1994) and we use it in this capacity. UNC-51 DN strongly suppressed defects in synapse maintenance (Fig 3d,e) and axon termination (Fig S3) caused by *rpm-1* (lf).

We now provide new data showing that UNC-51 DN does not affect axon guidance in mechanosensory neurons (Supplementary Fig 4). This indicates that UNC-51 DN can strongly suppress *rpm-1* phenotypes in both axon termination and synapse maintenance without causing any discernible axon guidance problems. Thus, the UNC-51 DN is an informative reagent that allowed us to more cleanly dissect suppression of *rpm-1*, and segregate these effects from UNC-51 effects on axon guidance. We emphasize that it is still plausible, in fact quite likely, that UNC-51 has an earlier role in axon guidance. However, our results suggest this is molecularly distinct from UNC-51 effects on axon termination and synapse maintenance. We discuss this further below.

2) Data included in our original manuscript (Fig 2 and 3), which we now present more clearly, shows that axon termination defects caused by *rpm-1* (lf) are not suppressed in *rpm-1; unc-14* double mutants. UNC-14 is a well-defined UNC-51 binding protein that functions with UNC-51 to regulate axon guidance (Ogura et al 1997; Lai & Garriga 2004). Thus, suppression of *rpm-1* (lf) by *unc-51* (lf) but not *unc-14* (lf) provides further molecular evidence to separate the role of UNC-51 in axon termination and synapse maintenance (downstream of RPM-1) from its known function in axon guidance.

3) Previously, UNC-51 and UNC-14 were shown to affect axon guidance by regulating Netrin receptor trafficking (Ogura & Goshima Development 2006). Therefore, we also tested the genetic relationship between *rpm-1* and two netrin receptors, *unc-5* and *unc-40/DCC*. Our results indicate that defects in ALM axon termination, PLM axon termination and PLM synapse maintenance caused by *rpm-1* (lf) are not suppressed in *rpm-1; unc-5* or *rpm-1; unc-40* double mutants (Fig S11). In fact, defects in synapse maintenance and ALM axon termination are increased in *rpm-1; unc-40* double mutants compared to single mutants (Fig S11B, C), which suggests RPM-1 acts in parallel to the UNC-40 Netrin receptor to regulate axon termination and synapse development.

4) The reviewer's comment about axon guidance further highlighted our need to do more extensive experiments with UNC-51 DN. While not directly requested by the reviewer, we thought it valuable to include new data on behavioural habituation that is mediated by the glutamatergic chemical synapses of the mechanosensory neurons. Our new results show that MosSCI expression of UNC-51 DN alone had no effect on habituation, but UNC-51 DN strongly suppresses habituation defects

in *rpm-1* mutants (Fig 3f, g). These behavioural results are consistent with our findings on *rpm-1* and UNC-51 DN in PLM synapse maintenance. These new results, taken in the context of our entire body of work, suggest that RPM-1 inhibition of UNC-51 and autophagy not only influences synapse maintenance, but also behavioural habituation which is mediated by these synapses. This new data strengthens our conclusions, and provides further experiments to highlight the value of the UNC-51 DN reagent, which as noted above does not impair axon guidance in mechanosensory neurons (Supplemental Fig 4).

We hope the reviewer agrees that our existing data, new data, and better written presentation carefully and thoroughly deal with this important consideration.

- pg 8: 'In unc-51 mutants, we observed synaptic branch loss (Fig. 3b, c).'.

It is not clear whether their data actually show the synaptic branch was formed first, then lost, or simply never formed.

>>>
We refer the reviewer to our prior study for complete developmental analysis of *rpm-1* function and RPM-1 subcellular localization during synapse maintenance (Borgen et al, *Elife*, 2019). This study shows that RPM-1 localizes to presynaptic terminals when they are first formed at 5 hours post-hatch and is present there into adulthood. Using a range of presynaptic markers, as well as analysis of bouton morphology and size, we showed that presynaptic terminals have slightly delayed, but normal assembly in *rpm-1* mutants. The primary defect in these animals is pronounced failure of synapse maintenance, which occurs between 16 and 24 hours post-hatch.

To further address this, we point to results with the UNC-51 DN. MosSCI expression of UNC-51 DN does not impair synaptic branches on its own (Supplemental Figure 4a, c). As a result, UNC-51 DN shows much stronger suppression of synapse maintenance defects in *rpm-1* mutants than what occurs in *rpm-1; unc-51* double mutants. We think single copy expression could be important here, as it allows UNC-51 DN to probably produce a milder loss of function for UNC-51 that allows suppression of RPM-1 without yielding axon guidance defects. Alternatively, UNC-51 DN might affect axon termination and synapse maintenance because it can preferentially impair autophagy (Chan… Tooze, *Mol Cell Biol*, 2009), but perhaps does not affect UNC-51 function in axon guidance that occurs via binding to UNC-14.

Thus, UNC-51 DN allows us to separate suppression of *rpm-1* (lf) effects on synapse maintenance from possible effects on synaptic branch extension. As a result, we have removed our qualifying statement at the end of the results section which the reviewer notes in their next point. We have also rewritten the final paragraph in this results section for better clarity and presentation. To further address this, we use "synapse maintenance" throughout the paper to more accurately and clearly describe this phenotype, and more clearly emphasize our prior work on this phenotype in *rpm-1* mutants.

At the end of this section, 'While there is no example of rpm-1 suppression affecting synaptic branch defects and not influencing synapse formation, our results do not rule out this possibility. More importantly, our results indicate that UNC-51 and UNC-14 have different functional relationships with RPM-1 during synapse formation.'.
It is not clear why the last statement is 'more important' for what reason.

>>>
We agree. We removed this statement, and also rewrote this paragraph of the Results to more clearly address the reviewer's point about UNC-51 effects on axon guidance.

-pg 13: ‘Unfortunately due to overexpression phenotypes (Fig. 4d), we could not consistently visualize mScarlet::UNC-51 at normal axon termination sites when it was expressed in mechanosensory neurons. ‘.

This is rather difficult to understand, as the data in Fig 4d show about 30% PLM being abnormal. Can they observe the remaining 70% normal PLM?

>>>
The reviewer’s point is reasonable. In fact, when wt UNC-51 is overexpressed it often leads to various axon guidance defects. Despite being able to assess the overall position of axon termination along the body axis and relative to other neurons for quantification parameters, the termination sites were often not wild type at the finer level, resulting in abnormal RPM-1::GFP puncta. As a result, we turned to UNC-51 KD which allowed us to more frequently and easily visualize normal axon termination sites.

To address this, we now include the following revised statement:
“Because of overexpression phenotypes (Fig. 4d, e) and effects on axon guidance (not shown), it was challenging to consistently visualize mScarlet::UNC-51 at normal axon termination sites when it was expressed in mechanosensory neurons. Therefore, we expressed UNC-51 KD in mechanosensory neurons.”

-pg 14, figure 7e, f, the data on ATG-9 rescue seems to show partial activity of ATG-9. Is this the case? statistic analysis should be applied to the data for rpm-1; atg-9+ ATG-9 rescue relative to rpm-1.

>>>
The reviewer is correct that this is only a partial rescue. We did use Mos single copy insertion for ATG-9 rescue, but still only observed partial rescue. Presumably this is because our promoter (P*mec-7*) might not be ideal for levels or timing of ATG-9 expression to rescue this phenotype. Because we used an *atg-9* cDNA for rescue, the lack of splicing could be an issue. Additionally, the transgene does not use the native *atg-9* 3’UTR, which is standard for MosSCI transgenes.

An alternative explanation for partial rescue might stem from our observation that overexpression of UNC-51 can affect axon termination and synapse maintenance. Thus, it is plausible that rescue experiments for ATG-9 also need to strike a balance between rescue and overexpression effects that could impair termination and synapse maintenance.

Also, the authors wrote ‘Finally, suppression of defects caused by rpm-1 (lf) was not as strong in rpm-1; atg-9 or rpm-1; epg-6 double mutants as rpm-1; unc-51 animals. This is most likely because UNC-51 is the primary activator of autophagy, …’.

Can they rule out that this is not due to unc-51’s roles in transport?

>>>
The reviewer’s comment is reasonable.

All our data across systems (including new data showing the human RPM-1 ortholog is sufficient to ubiquitinate ULK1, Fig 5j) suggests RPM-1 would locally regulate UNC-51 to influence autophagy. We also provide multiple layers of evidence that RPM-1 effects on UNC-51 differ from UNC-51 function in axon guidance and trafficking of Netrin receptors (see response above). Therefore, we think it most likely that *unc-51* (lf) suppresses *rpm-1* (lf) in axon termination and synapse maintenance due to effects on autophagy rather than its role in axon guidance.

To further address this issue, we also now provide new data with two more autophagy mutants, *epg-8/Atg14* (Fig 7c, e, f) and *bec-1* (Fig S10). Both EPG-8 and BEC-1 affect phagophore nucleation, a more upstream step in autophagy than events affected by ATG-9 and EPG-6/Wipi (Fig 7a). Results indicate that ALM and PLM axon termination defects and PLM synapse maintenance defects are all suppressed in *rpm-1; epg-8* (Fig 7c, e, f). *rpm-1; bec-1* double mutants show suppression of PLM synapse maintenance defects (Fig S10b) and ALM termination defects (Fig S10c), but not PLM termination defects (Fig S10a).

As the reviewer is likely aware, many autophagy mutants are not viable. Thus, we opted to work with mutants we were able to propagate. However, these alleles could be hypomorphic. This might also explain why suppressor effects are partial.

- Minor points on the style of writing: it seems that the authors have a tendency to repeat a number of statements or state too soon.
e.g. the top of pg 7 'We observed axon guidance defects in PLM neurons of unc-51 mutants, but this did not prevent us from evaluating axon termination.', which is already stated on pg 6.

>>>
We agree that these two sentences were too similar, though they were referring to outcomes in different neurons (ALM and PLM neurons). We removed this second sentence as per the reviewer's request.

Also, the conclusion paragraph on pg 7 'Overall, these genetic results support several conclusions. …' . In this reviewer' opinion, the three conclusions depend on the data in later Figures.

>>>

We have adjusted this paragraph to the following, which we think is reasonably supported by data in this and prior sections of the Results already presented.

"Taken as a whole, our genetic results support several conclusions. 1) UNC-51 is required to prevent premature termination. 2) UNC-51 is negatively regulated by RPM-1. 3) RPM-1 inhibition of UNC-51 is required for proper axon termination, but does not pertain to UNC-51/UNC-14 effects on axon guidance. These genetic findings are consistent with our proteomic and biochemical results suggesting UNC-51 is a putative RPM-1 ubiquitination substrate."

-generally, the authors should be consistent in writing whether they mean 'unc-51' or 'rpm-1' to be same as loss of function. e.g. pg 9. 'Our studies demonstrate that unc-51 suppresses rpm-1 (Fig. 2, 3)'. Presumably, they mean to say loss of function in unc-51?

>>>
We appreciate the reviewer pointing this out. We have corrected this throughout the manuscript.

-Labels, i.e. words and numbers, on panels in Fig 1e, and many graphs in Fig 2e, f, and like are too small.

>>>
We agree and have corrected throughout paper.

REVIEWERS' COMMENTS:

Reviewer #1 (Remarks to the Author):

The authors have addressed my main concerns. The authors should, however, include Borgen et al. (Development 2017) in the main text (it appears only in the supplement to reference the methods), and include an expanded description of this work that reflects the clarification provided in the response to reviews.

Reviewer #2 (Remarks to the Author):

The revised manuscript is significantly improved. The authors have addressed my major concerns.

Mondira Kundu

Reviewer #3 (Remarks to the Author):

The new data and text revision have significantly improved the manuscript. This reviewer would support its acceptance.

We are delighted the editors at *Nature Communciations* and all three reviewers are in support of publishing our work. Below we respond to a single remaining comment from Reviewer 1.

Reviewer #1 (Remarks to the Author):

The authors have addressed my main concerns. The authors should, however, include Borgen et al. (Development 2017) in the main text (it appears only in the supplement to reference the methods), and include an expanded description of this work that reflects the clarification provided in the response to reviews.

>>>
The reviewer makes an important point. We have included this citation and updated the text as follows:

"Prior work established that RPM-1 regulates axon termination in C. elegans mechanosensory neurons[39, 43]. Developmental time-course and in vivo live imaging studies showed that RPM-1 is initially localized to the growth cone where it regulates a protracted growth cone collapse process that results in axon termination[44]. Following termination of axon outgrowth, RPM-1 remains localized at the terminated axon tip."
